# *Salmonella* SopB suppresses post-transcriptionally regulated cytokine release to reduce early tissue inflammation and delay disease progression

**DOI:** 10.1038/s41467-026-74942-9

**Published:** 2026-07-06

**Authors:** Nour Diab, Chiun Huei Yong, Eva-Lena Stange, Marlène S. Birk, Matthias A. Schmitz, Stefan Düsterhöft, Jonas Pes, Kira Noemi Ferle, Isabel Karkossa, Kristin Schubert, Jörg Deiwick, Mihael Vucur, Tom Luedde, Natalia Torow, Andreas Ludwig, Aline Dupont, Joel Selkrig, Martin von Bergen, Michael Hensel, Kaiyi Zhang, Mathias W. Hornef

**Affiliations:** 1 Institute of Medical Microbiology, RWTH Aachen University Hospital, Aachen, Germany; 2 Institute of Molecular Pharmacology, RWTH Aachen University Hospital, Aachen, Germany; 3 Department Molecular Toxicology, Helmholtz Centre for Environmental Research GmbH - UFZ, Leipzig, Germany; 4 Division of Microbiology, University of Osnabrück, Osnabrück, Germany; 5 Department of Gastroenterology, Hepatology, and Infectious Diseases, Medical Faculty, Heinrich-Heine-University, Düsseldorf, Germany; 6 Helmholtz Centre for Infection Research, Braunschweig, Germany; 7 German Centre for Integrative Biodiversity Research (iDiv) Halle-Jena-Leipzig, Leipzig, Germany; 8 Institute of Biochemistry, University of Leipzig, Leipzig, Germany; 9 Euregional Microbiome Center, RWTH Aachen University Hospital, Aachen, Germany; 10 Present Address: HMU Health and Medical University Düsseldorf-Krefeld, Düsseldorf, Germany; 11 Present Address: Brandenburg University of Technology Cottbus-Senftenberg, Senftenberg, Germany

**Keywords:** Pathogens, Cell death and immune response, Experimental models of disease

## Abstract

*Salmonella enterica* subsp. *enterica* serovar Typhimurium (*S*. Typhimurium) manipulates cellular processes through the translocation of effector molecules into the host cell cytosol. Using a recently established neonatal *S*. Typhimurium infection model, we provide functional insights into how *Salmonella* outer protein B (SopB) suppresses early mucosal tissue inflammation and prolongs host survival. Mechanistically, SopB prevents a disintegrin and metalloprotease 17 (ADAM17) activation, plasma membrane translocation and the release of membrane-bound TNFα from enterocytes and reduces epithelial secretion of IL-18 via mTOR-controlled secretory autophagy. This abolishes the early epithelial transcriptional response and reduces immune cell recruitment and programmed cell death-mediated mucosal barrier disruption delaying disease progression. The immunosuppressive effect of SopB is independent of the C-terminally encoded phosphatidylinositol phosphatase and phosphotransferase activity but requires an intact N-terminal domain. Also, it is restricted to the neonatal mouse model characterised by *Salmonella* pathogenicity island (SPI)1 type 3 secretion system (T3SS)-dependent enterocyte invasion-driven mucosal translocation. Thus, here we demonstrate that SopB suppresses the early, post-transcriptional regulation of epithelial cytokine release in an inositol phosphatase-independent manner likely promoting pathogen transmission.

## Abstract (web summary)

*Salmonella enterica* serovar Typhimurium is a major bacterial pathogen that causes intestinal disease by invading host tissues and manipulating cellular functions. In this study, researchers investigated how one bacterial effector protein, *Salmonella* outer protein B (SopB), helps the pathogen evade early immune defences during infection.

## Introduction

*Salmonella enterica* subsp. *enterica* sv. Typhimurium (*S*. Typhimurium) is an important human enteropathogen with a high disease burden in children and neonates worldwide^1^. It uses the *Salmonella* pathogenicity island (SPI)1-encoded type three secretion system (T3SS) to translocate effector molecules into intestinal epithelial cells. These effector molecules induce bacterial internalisation, shape formation of the *Salmonella*-containing vacuole (SCV) and manipulate host cell processes to promote intracellular survival and proliferation^2–4^. Among the effector molecules translocated by the SPI1-T3SS is SopB, a phosphatidylinositol phosphate 4 and 5 (PtdIns(4,5)P_2_) phosphatase and phospho-transferase/isomerase that generates PtdIns(3,4,5)P_3_ and PtdIns(3,4)P_2_ in a non-canonical phosphoinositide 3-kinase (PI3K)-independent manner^5–7^.

Through its C-terminal phosphatase domain (aa 357–561), SopB together with SopE/SopE_2_ activates the Rho GTPases Cdc42^8^, enriches RhoJ, RhoB, RhoH and R-Ras1^9^, PtdIns(3,4)P_2_ and PtdIns(3,4,5)P_3_^10^, Arf GEF ARNO^11^, annexin A2^12,13^, Myo6^14^, as well as SNX9^15^ and SNX18^16^ at the entry site, leading to actin remodelling, ruffling, membrane fission and uptake by intestinal epithelial cells, the first step in invasive infection^6,17^. SopB is then multi-mono-ubiquitinated and recruited to the endosomal membrane, where it reduces the negative surface charge of the *Salmonella-*containing vacuole (SCV) by removing PtdIns(4,5)P_2_ and phosphatidylserine, thereby inhibiting Rab recruitment and lysosomal fusion^18–21^. In addition, SopB induces a sustained phosphatase-dependent Akt phosphorylation protecting infected cells from cell death^20,22–27^. SopB expression persists for many hours in vivo^20,28^. More recently, the N-terminal GTPase binding domain (residues 117-168) of SopB has been shown to facilitate phosphatase-independent interaction with Cdc42 leading to actin fibre disruption, cell cycle arrest and MAP kinase signalling in yeast cells^29–33^. However, the functional role of SopB during in vivo infection and, in particular, the influence of the N-terminal domain has not been investigated.

Using our oral neonatal mouse infection model that requires SPI1-dependent enterocyte invasion^3^, we have previously shown that SopB is not required for enterocyte invasion and SCV formation^4^. Here, we analysed the role of SopB during the early course of the disease using isogenic mutant strains, co-culture experiments with polarised m-IC_cl2_ cells and intestinal epithelial stem cell organoids, and oral infection of mice deficient in innate immune signalling and cell death pathways in combination with flow cytometry, global phosphoproteome and transcriptome analyses, affinity enrichment of SopB-associated host proteins by mass spectrometry as well as AlphaFold-Multimer (AFM) protein-protein interaction prediction. Unexpectedly, we observed an accelerated disease progression and increased mortality after infection with SopB-deficient *S*. Typhimurium. A detailed analysis revealed a suppressive effect of SopB on a disintegrin and metalloprotease 17 (ADAM17)/TNF converting enzyme (TACE) activity and secretory autophagy, reducing early cytokine release with subsequent reduction of chemokine expression and immune cell recruitment, decreased enterocyte cell death and prolonged host survival.

## Results

### SopB delays disease progression and immune stimulation

**Fig. 1:**
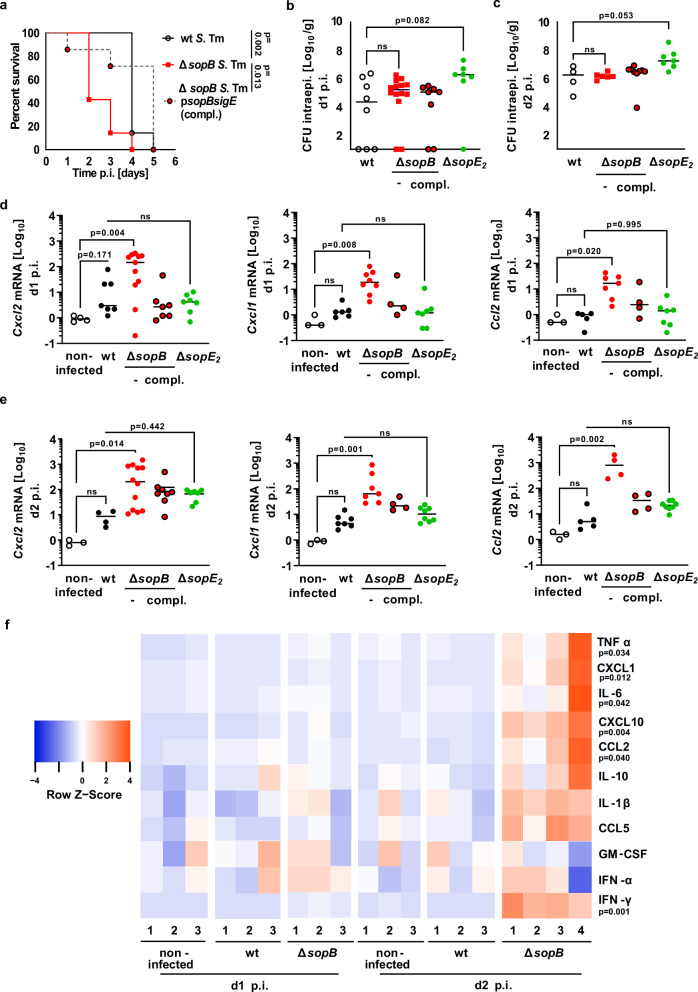
Host survival and infection-induced chemokine and cytokine expression. **a** Kaplan-Meier curve of 1-day-old C57BL/6 wildtype mice orally infected with 100 CFU wt (*n* = 7), *sopB*-deficient (Δ*sopB*, *n* = 7), or *sopB*-deficient *S*. Typhimurium complemented in trans with *sopB* and its chaperon *sigE* (Δ*sopB* p*sopB sigE*, compl., *n* = 7). **b**–**e** 1-day-old mice were left non-infected (**d**, *n* = 3- 4; **e**, *n* = 3) or orally infected with wt (**b**, *n* = 8; **c**, *n* = 4; **d**, *n* = 5–10; **e**, *n* = 4–7), *sopB*-deficient (Δ*sopB*, **b**, *n* = 14; **c**, *n* = 6; **d**, *n* = 7–11; **e**, *n* = 4–12), *sopB*-deficient *S*. Typhimurium complemented in trans with *sopB* and its chaperon *sigE* (Δ*sopB* p*sopBsigE*, compl., **b**, *n* = 8; **c**, *n* = 9; **d**, *n* = 4–7; **e**, *n* = 4–8) or *sopE*_*2*_ deficient *S*. Typhimurium (Δ*sopE*_*2*_,** b**–**d**, *n* = 7; **e**, *n* = 8). Number of intracellular *S*. Typhimurium in isolated intestinal epithelial cells at day 1 (**b**) and 2 (**c**) post infection (p.i.). One data point represents one animal, at least two independent experiments. Mean. *Cxcl1*, *Cxcl2* and *Ccl2* mRNA expression in total isolated intestinal epithelial cells at day 1 (**d**) and day 2 (**e**) p.i. Values were normalised to the house keeping gene *Hprt* and are showed as fold expression over uninfected age-matched control animals. One data point represents one animal from at least two independent experiments, the median. **f** Colour-scaled heat map (z-score) showing the concentration of the indicated cytokines and chemokines in sera of uninfected age-matched animals (*n* = 3), or mice infected at day 1 after birth with wt (*n* = 3) or Δ*sopB S*. Typhimurium (*n* = 3-4) at day 1 and 2 p.i. Each column represents one animal. Statistical analysis by log-rank (Mantel-Cox) test (**a**), Kruskal-Wallis test combined with Dunn’s multiple comparison test (**b**–**e**); two-way ANOVA with Sidak’s multiple comparison test (**f**). ns, non-significant; *, *p* < 0.05; **, *p* < 0.01, ***, *p* < 0.001.

In most cases, pathogens with genetic deletions in important virulence factors exhibit an attenuated phenotype. In contrast, infection of newborn mice with a *Salmonella enterica* subsp. *enterica* sv. Typhimurium (*S*. Typhimurium) strain lacking the *Salmonella* pathogenicity island (SPI)1-type 3 secretion system (T3SS) translocated phosphatidyl-inositol phosphatase SopB (Δ*sopB*) resulted in significantly accelerated disease progression and earlier mortality as compared to infection with wildtype (wt) *S*. Typhimurium (Fig. 1a). Complementation (compl.) with *sopB* together with its chaperone *sigE* reversed this phenotype. Despite reports and own in vitro evidence for a role of SopB in enterocyte invasion (Supplementary Fig. 1a, b), no difference was observed between the number of intraepithelial Δ*sopB* and wt *S*. Typhimurium bacteria in vivo (Fig. 1b, c) or bacterial counts in total mesenteric lymph node, liver and spleen tissue at day 1 and 2 p.i. (Supplementary Fig. 1c, d)^8,11,15,21^. In contrast, intestinal epithelial expression of the chemokines *Cxcl2*, *Cxcl1* and *Ccl2* as well as the antimicrobial protein *Reg3g* was strongly increased at day 1 p.i. with Δ*sopB S*. Typhimurium, whereas it remained unaltered after infection with wt *S*. Typhimurium or with *S*. Typhimurium deficient in the important SPI1-T3SS translocated effector molecule SopE_2_ (Δ*sopE*_2_) (Fig. 1d, Supplementary Fig. 1e). At day 2 p.i. *Cxcl2*, *Cxcl1, Ccl2, Reg3g* and *Cxcl5* expression also started to increase after wt or Δ*sopE*_2_ *S*. Typhimurium infection, but was still significantly higher expressed in animals infected with Δ*sopB S*. Typhimurium (Fig. 1e and Supplementary Fig. 1f). Global transcriptome analysis at day 1 p.i. confirmed the increased expression of innate immune genes after Δ*sopB S*. Typhimurium infection (Supplementary Fig. 1g). Also, the systemic immune response was affected. Serum cytokine levels of IFN-γ, TNF-α, and IL-6, as well as chemokine levels of CXCL1, CCL2, and CXCL10, were significantly higher in Δ*sopB* as compared to wt *S*. Typhimurium-infected mice at day 2 p.i. (Fig. 1f and Supplementary Fig. 1h). These results suggest that the SPI1 effector SopB controls the early local immune stimulation, but also the subsequent systemic cytokine response in the neonatal host in vivo, reducing disease progression and delaying infection-induced mortality.

### SopB abolishes the early recruitment of inflammatory cells and mucosal tissue damage

**Fig. 2:**
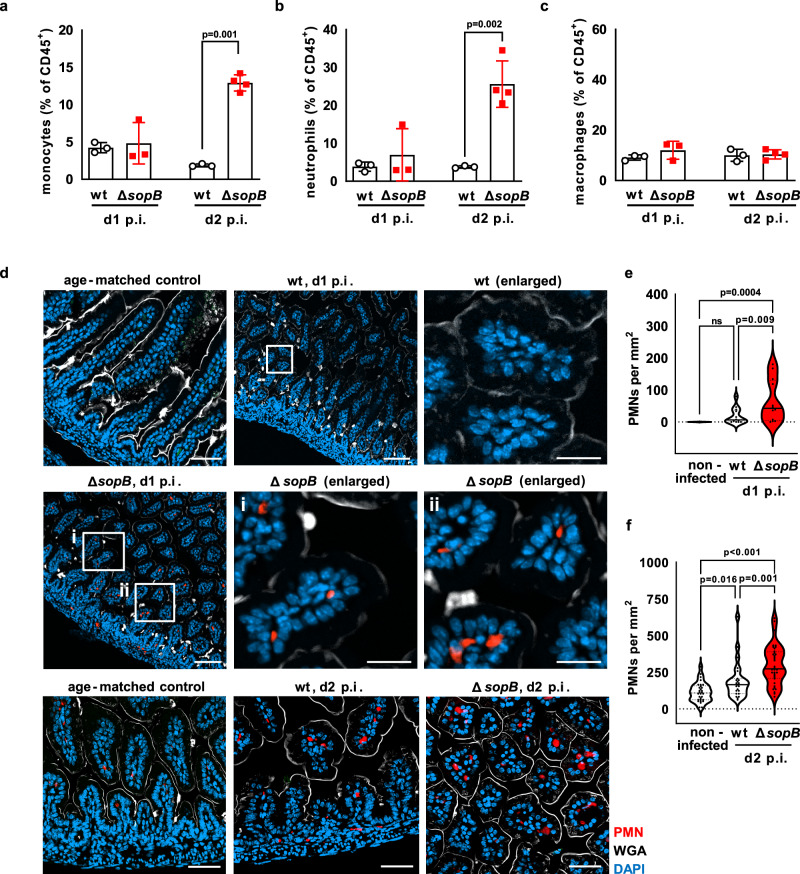
Immune cell infiltration in mucosal tissue. **a**–**c** 1-day-old C57BL/6 wildtype neonates were orally infected with 100 CFU wt (*n *= 3 at day 1 and 2 p.i.) or *sopB*-deficient (Δ*sopB*, *n* = 3 and *n *= 4 at day 1 and 2 p.i., respectively) *S*. Typhimurium. Flow cytometric analysis of *lamina propria* Ly6C^hi^Ly6G^-^CD11b^+^ MHCII^lo/-^CD45^+^DAPI^-^ monocytes (**a**), Ly6G^+^Ly6C^int^CD11b^+^ MHCII^lo/-^CD45^+^DAPI^-^ neutrophils (**b**) and CD64^+^MHCII^+^CD45^+^DAPI^-^ macrophages (**c**). Mean ± SD.** d**–**f** Immunostaining (**d**) of small intestine tissue sections of age-matched uninfected animals and neonate mice infected with wt or *sopB*-deficient (Δ*sopB*) *S*. Typhimurium at day 1 and 2 p.i. for Ly6G^+^ neutrophils (PMN, red). WGA (white) and DAPI (blue). Bar = 50 µm, white boxes indicate the position of the enlarged images, insert i and ii, 20 µm. Representative images are shown. **e**,**f** Quantification of the number of Ly6G^+^ neutrophils (PMNs) per mm^2^ of small intestinal tissue at day 1 p.i. analysing a total area of 0.38 mm^2^, and at day 2 p.i. analysing a total area of 0.10 mm^2^. Uninfected, age-matched neonates (4 or 8–11 images per animal at day 1 or 2 p.i., respectively, both *n* = 3) or neonates infected with wt (4 or 4–12 images per animal at day 1 or 2 p.i., respectively, both *n* = 3) or Δ*sopB S*. Typhimurium (4 or 5–17 images per animal at day 1 or 2 p.i., respectively, both *n* = 3). Statistical analysis by two-way ANOVA with Tukey’s multiple comparison test (**a**–**c**) and Kruskal-Wallis combined with Dunn’s multiple comparison test (**e**,** f**). ns, non-significant; *, *p* < 0.05; **, *p* < 0.01; ****, *p* < 0.0001.

**Fig. 3:**
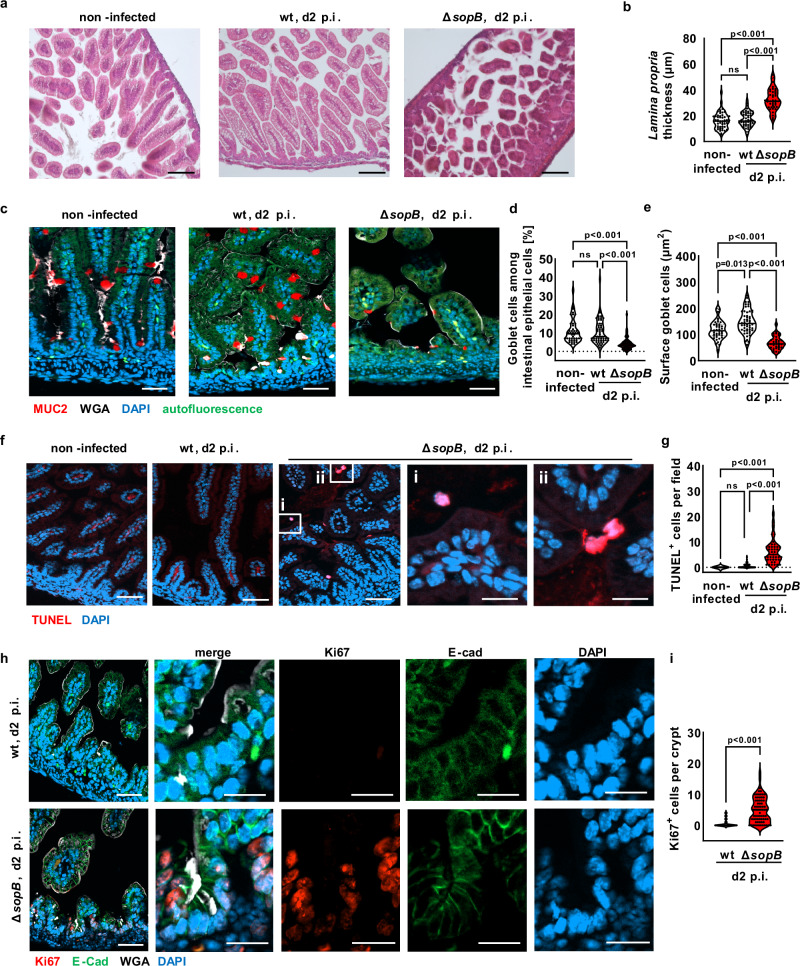
Histological characterisation of the neonatal small intestine following infection. **a** H&E stained tissue sections of non-infected, wt or Δ*sopB S*. Typhimurium infected neonates at day 2 p.i. Bar = 100 µm. **b** *Lamina propria* depth in 15-20 areas per non-infected (*n* = 3), wt (*n* = 3), or Δ*sopB*-infected (*n* = 3) animal. **c** MUC2 immunostaining (red) in tissue sections of non-infected (*n* = 3), wt (*n* = 3), or Δ*sopB S*. Typhimurium-infected (*n* = 3) animals at day 2 p.i. Counterstaining with WGA (white), DAPI (blue); autofluorescence (green). Bar = 50 µm. **d** Percentage of goblet cells among intestinal epithelial cells in non-infected (*n* = 3), wt (*n* = 3), or Δ*sopB* (*n* = 3) *S*. Typhimurium-infected animals at day 2 p.i. 10–20 images with a size of 312 µm x 250 µm were evaluated per animal. **e** MUC2^+^ goblet cell size in tissue sections of uninfected (*n* = 3), wt (*n* = 3) or Δ*sopB* (*n* = 3) *S*. Typhimurium-infected animals at day 2 p.i. 11–21 goblet cells (µm^2^) were analysed on each section. **f** TUNEL staining (red) of tissue sections of non-infected (*n* = 2), wt (*n* = 5), or Δ*sopB* (*n* = 4) *S*. Typhimurium-infected animals at day 2 p.i. White boxes indicate enlarged images (i, ii). Counterstaining with DAPI (blue). Bar=50 µm; insert, 20 µm. **g** Number of TUNEL^+^ cells in tissue sections of uninfected (*n *= 2), wt (*n* = 5), or Δ*sopB* (*n* = 4) *S*. Typhimurium-infected animals at day 2 p.i. 9 –12 images with the size of 624 µm x 501 µm were evaluated per animal. **h** Ki67 (red) immunostaining on tissue sections of neonates infected with wt (*n* = 3) or Δ*sopB* (*n* = 4) *S*. Typhimurium at day 2 p.i. Counterstaining with E-cadherin (green), WGA (white), and DAPI (blue). Bar, 50 µm, insert, 20 µm. **i** Number of Ki67^+^ cells in tissue sections of uninfected (*n* = 2), wt (*n* = 3), or Δ*sopB* (*n* = 4) *S*. Typhimurium-infected animals at day 2 p.i. 4–29 intervillus junctions (early crypts) were analysed per section. Representative images are shown. Quantified data are shown as individual points in violin plots, and solid lines represent the median. Statistical analysis by Kruskal-Wallis combined with Dunn’s multiple comparison test (**b**, **d**,** and g**), one-way ANOVA with Tukey’s multiple comparison test (**e**) and Mann-Whitney test (**i**). ns, non-significant; *,* p* < 0.05; ****, *p* < 0.0001.

Consistent with the observed increase in early chemokine expression, the number of monocytes and neutrophilic granulocytes recruited to the mucosal small intestinal tissue and detected by flow cytometry was strongly enhanced at day 2 p.i. with Δ*sopB* as compared to wt *S*. Typhimurium (Fig. 2a, b and Supplementary Fig. 2a), whereas the number of tissue resident macrophages was not altered (Fig. 2c). Consistently, immunostaining of the small intestine revealed enhanced numbers of neutrophilic granulocytes (PMNs) already at day 1 p.i. and even more pronounced at day 2 p.i. (Fig. 2d–f). Histological analysis further demonstrated significant differences in the mucosal tissue architecture between Δ*sopB* and wt *S*. Typhimurium-infected mice. Infection with Δ*sopB* as compared to wt *S*. Typhimurium led to enhanced thickening of the submucosal tissue as a sign for oedema formation (Fig. 3a, b), a reduced MUC2^+^ signal that in the context of enhanced tissue inflammation most likely is caused by goblet cell depletion and incomplete differentiation of new replacements from progenitors (Fig. 3c–e)^34^, and elevated numbers of mostly exfoliating TUNEL^+^ enterocytes suggesting increased epithelial cell death (Fig. 3f, g). In addition, Δ*sopB S*. Typhimurium-infected mice exhibited increased epithelial cell proliferation, illustrated by elevated numbers of Ki67^+^ cells, suggesting epithelial repair (Fig. 3h, i). Thus, SopB suppresses early tissue inflammation upon *S*. Typhimurium infection, maintaining mucosal tissue integrity and function. Notably, this phenotype was only observed in the neonatal mouse infection model. No increase in early cytokine and chemokine expression, inflammatory cell recruitment, or disease severity was observed after infection of adult C57BL/6 mice with Δ*sopB S*. Typhimurium ATCC14028 (Supplementary Fig. 3a–f).

### SopB inhibits programmed cell death and TNFα-mediated disease progression

**Fig. 4:**
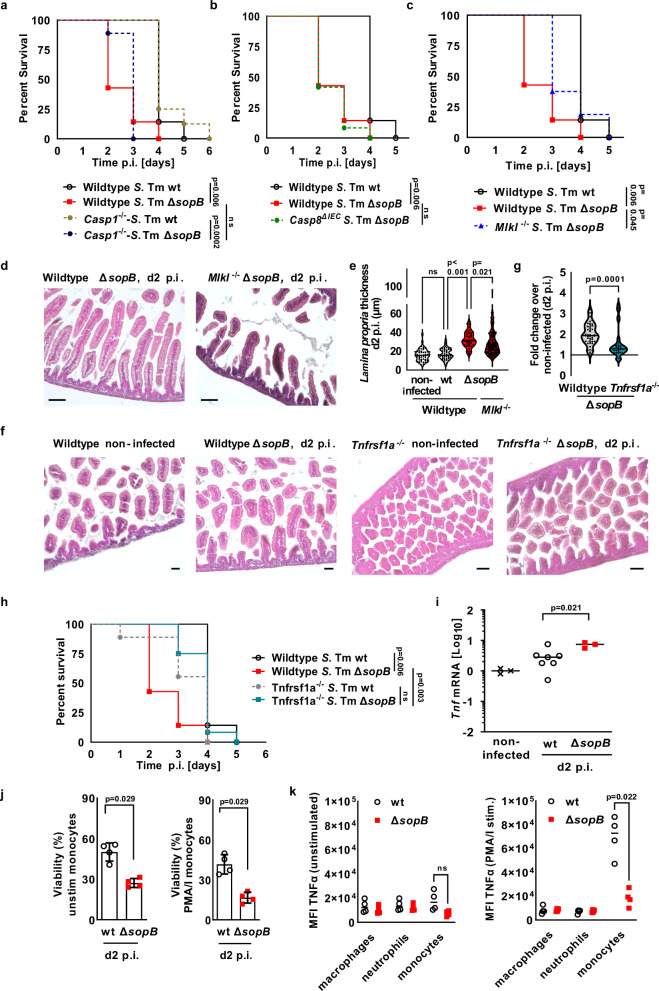
Mechanisms of disease progression at day 2 p.i. **a**–**c** Kaplan-Meier curve of wildtype (*n* = 7, both groups) and *Casp1*^-/-^ (*n* = 8-9) (**a**), wildtype and intestinal epithelium-specific caspase 8-deficient (*Casp8*^ΔIEC^, *n* = 12) (**b**), and wildtype and *Mlkl*^-/-^ (*n* = 16) (**c**) neonates infected with wt or Δ*sopB S*. Typhimurium. **d** H&E staining of wildtype (*n* = 3) and *Mlkl*^-/-^ (*n* = 3) Δ*sopB S*. Typhimurium infected neonates at day 2 p.i. Bar = 50 µm. **e** Depth of the *lamina propria* in 4–12 areas per wildtype or *Mlkl*^-/-^ mouse infected with wt or Δ*sopB S*. Typhimurium at day 2 p.i. Individual data points and median. **f** H&E staining of neonatal wildtype (*n* = 3) and *Tnfrsf1a*^-/-^ (*n* = 5) mice infected with Δ*sopB S*. Typhimurium at day 2 p.i. Bar = 50 µm. **g** Fold change of the depth of the *lamina propria* in Δ*sopB S*. Typhimurium-infected over non-infected age-matched wildtype (*n* = 3 and 3) and *Tnfrsf1a*^-/-^ mice (*n* = 3 and 3) at day 2 p.i. 3–9 fields were analysed per animal. Individual data points and median. **h** Kaplan-Meier curve of wildtype (*n *= 7) and *Tnfrsf1a*^-/-^ (*n* = 9 and 12) neonates infected with wt or Δ*sopB S*. Typhimurium. **i** *Tnf* mRNA in total gut tissue from age-matched non-infected (*n* = 3), wt (*n* = 7), or Δ*sopB* (*n* = 3) *S*. Typhimurium-infected animals at day 2 p.i. Fold increase, values are normalised to *Hprt*. At least two independent experiments. One data point represents one animal, Median. **j** Viability of unstimulated (left panel) or PMA/ionomycin stimulated (right panel) *lamina propria* monocytes of wt (circles, *n* = 4) or Δ*sopB* (red squares, *n* = 4) *S*. Typhimurium infected animals. Mean ± SD. **k** TNFα mean fluorescence intensity (MFI) after intracellular cytokine staining at day 2 p.i. from wt (circles, *n* = 4) or Δ*sopB* (red squares, *n* = 4) *S*. Typhimurium*-*infected animals without (left panel) or after stimulation with PMA/ionomycin (right panel). Median. (**a**–**c**,** h**) The groups of infected wildtype mice are identical to Fig. 1a. Log-rank (Mantel-Cox) test (**a**–**c**,** h**), Kruskal–Wallis test with Dunn’s multiple comparisons post-test (**e**), one-way ANOVA with Tukey’s multiple comparison test (**i**), Mann-Whitney test (**g**,** j**), and two-way ANOVA with Sidak’s multiple comparison test (**k**). ns, non-significant; *, *p* < 0.05; **, *p* < 0.01; ***, *p* < 0.001; ****, *p* < 0.0001.

SopB has been shown to induce pro-survival signalling, suggesting that increased programmed cell death may occur in the absence of SopB with downstream effects on mucosal tissue integrity and inflammation^20,22–25,35^. Therefore, we next infected mice impaired in the different cell death pathways, and monitored their survival, intestinal tissue morphology, bacterial organ counts, and epithelial gene expression. The accelerated disease progression and earlier mortality after Δ*sopB versus* wt *S*. Typhimurium infection was less pronounced but still significant in caspase 1 (Casp1)- and ASC-deficient mice, both impaired in the proinflammatory cell death form pyroptosis (Fig. 4a and Supplementary Fig. 4a). Early mortality after Δ*sopB S*. Typhimurium infection was preserved in intestinal epithelial cell-specific caspase 8-deficient (*Casp8*^ΔIEC^) animals, impaired in extrinsic apoptosis (Fig. 4b) and no significant increase in the expression of the endogenous apoptosis regulator *Bcl2* and the nitric oxide generating protein iNOS (*Nos2*) was observed although a tendency of elevated *Nos2* expression was noted in Δ*sopB S*. Typhimurium-infected wildtype animals (Supplementary Fig. 4b, c) in contrast to what has previously been reported after infection of adult mice^20,26^. Only *Mlkl*^-/-^ animals impaired in necroptosis infected with Δ*sopB S*. Typhimurium exhibited a significantly prolonged survival (Fig. 4c), and the histological analysis of *Mlkl*^-/-^ mice revealed reduced thickening of the submucosal tissue in Δ*sopB S*. Typhimurium-infected mice (Fig. 4d, e). Importantly, the number of intraepithelial Δ*sopB S*. Typhimurium, the bacterial organ load in the mesenteric lymph nodes and liver and the epithelial expression of *Cxcl2* mRNA were not significantly altered in the absence of MLKL, ASC or Caspase 1 at day 2 p.i. (Supplementary Fig. 4d–g). Together, these results suggest that necroptosis and to some degree pyroptosis contribute to the accelerated disease progression and reduced survival after Δ*sopB S*. Typhimurium infection but likely act downstream of the observed early inflammatory response at day 1 p.i^36^.

Necroptosis is induced by TNFα, and this cytokine has previously been shown to promote *Salmonella*-induced mucosal inflammation, prompting us to test the phenotype of TNF receptor 1 (TNFR1) deficient (*Tnfrsf1a*^*-/-*^) mice after Δ*sopB S*. Typhimurium infection^37^. The Δ*sopB S*. Typhimurium infection-induced tissue thickening of the *lamina propria* at 2 days p.i. was abolished in the absence of TNFR1 (Fig. 4f, g). More importantly, early mortality observed after Δ*sopB S*. Typhimurium infection was absent in *Tnfrsf1a*^-/-^ animals (Fig. 4h). Notably, the bacterial organ load in the intestinal epithelium, mesenteric lymph node and liver tissue was not significantly altered in the absence of TNFR1 at day 2 p.i. (Supplementary Fig. 4d–f). Consistent with a functional role of TNFα, we found that the *Tnf* mRNA concentration was moderately but significantly enhanced in total small intestinal tissue at day 2 p.i. with Δ*sopB S*. Typhimurium (Fig. 4i). Among immune cells, tissue monocytes exhibited a significantly reduced viability both under non-stimulated and PMA/ionomycin stimulated conditions when isolated at day 2 p.i. from Δ*sopB* as compared wt *S*. Typhimurium-infected animals (Fig. 4j and Supplementary Fig. 5a–c). TNFα may therefore be released from monocytes that were pushed to cell death and cell lysis in the inflamed Δ*sopB S*. Typhimurium-infected tissue environment. Consistently, we observed a significantly lower mean fluorescent intensity (MFI) for intracellular TNFα in stimulated monocytes isolated from Δ*sopB* as compared to wt *S*. Typhimurium-infected animals (Fig. 4k and Supplementary Fig. 5d). In addition to the increased *Tnf* mRNA levels in Δ*sopB S*. Typhimurium-infected intestinal tissue, these findings are consistent with an enhanced cytokine release in the absence of SopB. The released TNFα may, in turn, induce enterocyte programmed cell death and contribute to the accelerated systemic disease progression after Δ*sopB S*. Typhimurium infection of neonatal mice^36^.

### SopB-mediated suppression of ADAM17 activity and TNFα secretion

**Fig. 5:**
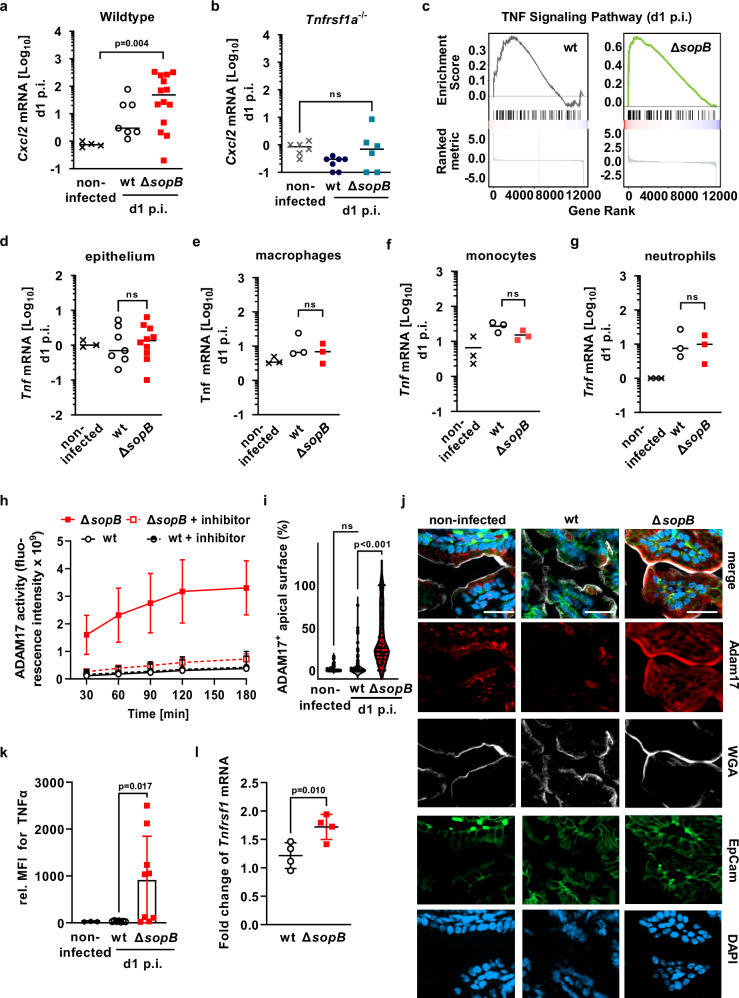
Influence of SopB on ADAM17 activation and TNF secretion at day 1 p.i. **a**,** b** Epithelial *Cxcl2* mRNA expression in non-infected, wt or Δ*sopB S*. Typhimurium-infected wildtype (**a**) and *Tnfrsf1a*^-/-^ (**b**) mice. Wildtype, *n* = 4, 7, 14 (identical to Fig. 1d) and *Tnfrsf1a*^-/-^, *n* = 6, 7, 6 animals, respectively. Normalised to *Hprt*; fold expression over non-infected animals. ≥ 2 independent experiments, median. **c** Gene set enrichment analysis (GSEA) using bulk RNA Seq data from epithelial cells isolated at day 1 p.i. from wt (*n* = 4) and Δ*sopB* (*n* = 4) *S*. Typhimurium-infected wildtype neonates (see Supplementary Fig. 1g). **d**–**g** *Tnf* mRNA expression in epithelial cells (**d**), CD64^+^MHCII^+^CD45^+^DAPI^-^ macrophages (**e**), Ly6C^hi^Ly6G^-^CD11b^+^ MHCII^lo/-^CD45^+^DAPI^-^ monocytes (**f**), and Ly6G^+^Ly6C^int^CD11b^+^ MHCII^lo/-^CD45^+^DAPI^-^ neutrophils (**g**) from non-infected (*n* = 3), wt (*n* = 7), or Δ*sopB* (*n* = 10) *S*. Typhimurium-infected mice at day 1 p.i. **d** or sorted from non-infected (*n* = 3), wt (*n* = 3), or Δ*sopB* (*n* = 3) *S*. Typhimurium-infected neonates at day 1 p.i. **e**–**g** Normalised to *Hprt*; median. **h** Epithelial cells from neonates isolated at day 1 p.i. with wt (*n* = 3) or Δs*opB S*. Typhimurium (*n* = 3) incubated with ADAM17 substrate +/– a specific ADAM17 inhibitor. Activity was quantified at Ex/Em = 320 nm/420 nm. Mean ± SD. **i** ADAM17^+^ relative to the total surface [%]. 86-88 villi per tissue from wt (*n* = 2), Δ*sopB S*. Typhimurium (*n* = 5) infected and non-infected animals (*n* = 2). **j** ADAM17 immunostaining (red) in non-infected, wt or Δ*sopB S*. Typhimurium-infected neonates. EpCam (green), wheat germ agglutinin (WGA, white) and DAPI. Bar = 25 µm. **k** TNFα (MFI) released from tissue segments from non-infected (*n* = 3), wt (*n* = 7), or Δ*sopB* (*n *= 9) *S*. Typhimurium-infected neonates. Normalised to non-infected animals. Mean ± SD of two independent experiments. **l** *Tnfrsf1a* mRNA expression in epithelial cells at day 1 p.i. with wt or Δ*sopB S*. Typhimurium (wt, *n *= 4; Δ*sopB*, *n* = 4). Values from Supplementary Fig. 1g. Fold change over the mean of non-infected animals (*n* = 4). Median. Statistical analysis by Kruskal-Wallis combined with Dunn’s multiple comparison test (**a**,** b**,** e**,** g**,** i**), one-way ANOVA with Tukey’s multiple comparison test (**d**,** k**,** f**), and Student’s *t* test (**l**). ns, non-significant; *,* p* < 0.05; **, *p* < 0.01; ***, *p* < 0.001; ****, *p* < 0.0001.

Mice infected with Δ*sopB S*. Typhimurium exhibited a strong, up to 100-fold increase in epithelial expression of the chemokine *Cxcl2* as early as at day 1 p.i., whereas no significant difference was noted between uninfected and wt *S*. Typhimurium-infected animals at this early stage of the infection (Fig. 5a). The difference between Δ*sopB* and wt *S*. Typhimurium-infected mice appeared surprisingly strong given the described requirement of enterocyte invasion for immune stimulation and the low number of infected and thus potentially SopB-manipulated enterocytes at this early stage of the infection^3,38^. A possible explanation could be the existence of a soluble mediator amplifying the cellular response upon Δ*sopB S*. Typhimurium infection. Indeed, analysis of *Tnfrsf1a*^-/-^ mice revealed that the early increase in epithelial *Cxcl2* expression in Δ*sopB S*. Typhimurium-infected mice at day 1 p.i. was completely dependent on intact TNFα receptor signalling (Fig. 5b). Similarly, almost all genes induced in wildtype mice by Δ*sopB* but not wt *S*. Typhimurium-infected mice (Supplementary Fig. 1g) remained unaltered in *Tnfrsf1a* ^-/-^ mice (Supplementary Fig. 6a). Consistently, gene set enrichment analysis (GSEA) of the small intestinal epithelial transcriptome at day 1 p.i. identified an enrichment of genes associated with TNF receptor signalling after Δ*sopB* but not wt *S*. Typhimurium infection (Fig. 5c). These findings suggested the existence of an early wave of TNFα secretion during the first 24 h of infection in addition to the TNFα-mediated tissue destructive effects described at later stages (day 2) of the Δ*sopB S*. Typhimurium infection (Fig. 4). Intestinal epithelial cells and various immune cells have been shown to increase their TNFα expression in response to *Salmonella* infection^37,39^. However, neither enterocytes nor immune cells such as monocytes, macrophages, or neutrophilic granulocytes isolated from the small intestine exhibited enhanced *Tnfa* mRNA levels at day 1 p.i. with Δ*sopB* as compared to wt *S*. Typhimurium (Fig. 5d–g).

In addition to the transcriptional regulation, TNFα is regulated on the posttranscriptional level by cleavage and release of membrane bound cytokine molecules by the TNFα converting enzyme (TACE), also called a disintegrin and metalloprotease 17 (ADAM17). ADAM17-mediated cleavage is activated by MAPK p38 and ERK^40^. Moreover, ADAM17-mediated TNFα release is influenced by Cdc42, a known interaction partner of SopB^41,42^. Using a fluorescent ADAM17 reporter in combination with a highly ADAM17-specific pharmacological inhibitor, we were able to detect ADAM17 activity in total intestinal epithelial cells isolated from mice at 1 day p.i. with Δ*sopB* but not wt *S*. Typhimurium (Fig. 5h and Supplementary Fig. 6b). In accordance, we also detected a significantly increased ADAM17^+^ area of the apical plasma membrane of the small intestinal epithelium in mice infected with Δ*sopB* but not wt *S*. Typhimurium by immunostaining (Fig. 5i, j). Consistently, the release of TNFα protein from total small intestinal tissue obtained from mice infected for 1 day with Δ*sopB S*. Typhimurium and incubated for 2 h in vitro was significantly higher than the TNFα release from tissue of mice infected for 1 day with wt *S*. Typhimurium (Fig. 5k). Finally, epithelial *Tnfrsf1a* mRNA expression encoding the TNFα receptor 1 (TNFR1) was increased at day 1 p.i. after Δ*sopB* but not wt *S*. Typhimurium infection likely amplifying TNFα-mediated signals (Fig. 5l). Thus, SopB inhibits ADAM17 activation and plasma membrane translocation, the release of TNFα and TNFR1-mediated cell signalling in neonatal mice.

### Analysis of the interaction of SopB with host cell processes

**Fig. 6:**
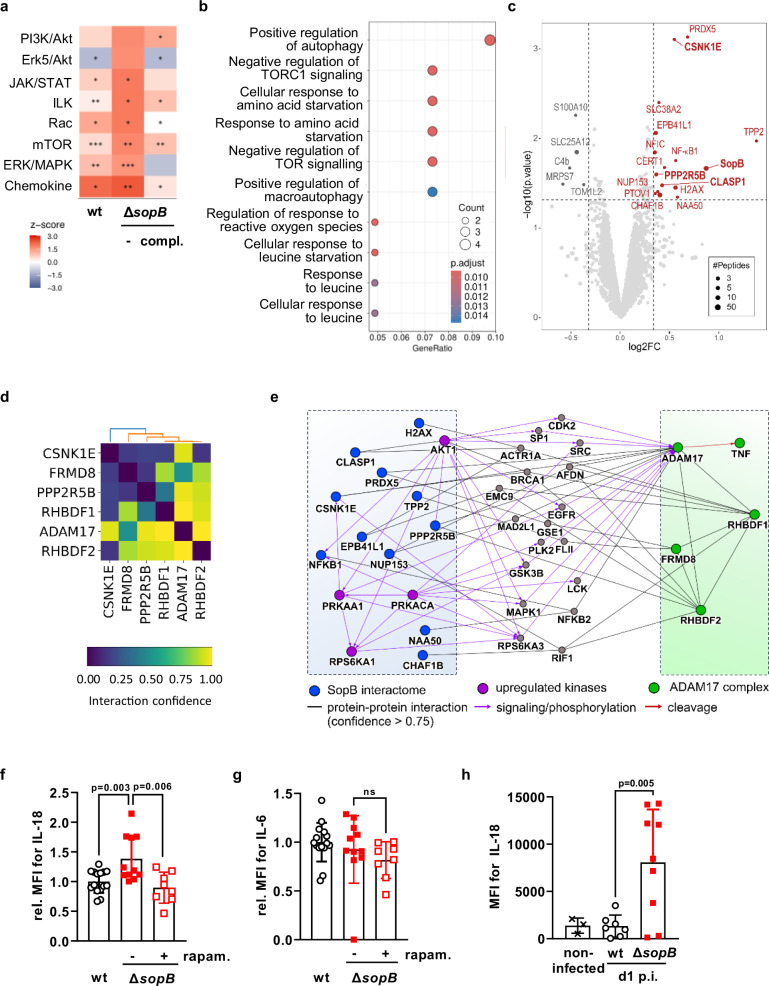
Analysis of the interaction of SopB with host cell processes. **a** Phosphoproteome of m-IC_cl2_ cells left untreated (*n* = 4) or infected with wt (*n* = 4), Δ*sopB* (*n *= 4) or Δ*sopB* p*sopBsigE S*. Typhimurium (compl., *n* = 4) for 1 h. Enriched pathways (Qiagen Ingenuity Pathway Analysis) based on significantly altered phosphosites using the IPA Z-score activation scaling. Right-tailed Fisher’s Exact Test, adjusted by Benjamini-Hochberg correction. *, adj.*p* < 0.05; **, adj.*p* < 0.01; ***, adj.*p* < 0.001 indicate differences in the abundance from non-infected cells. **b** Top ten GO terms based on genes significantly upregulated in wt (*n* = 4) relative to Δ*sopB* (*n* = 4) *S*. Typhimurium-infected neonates at day 1 p.i. **c** Affinity purification and mass spectrometric analysis of SopB-associated host cell proteins (red). x-axis, log₂ fold changes (log₂FC) relative to control; y-axis, −log₁₀(p-value) for differential abundance; dashed lines, thresholds for differential enrichment (|log₂FC | ≥ 0.33) and nominal significance (two-sided *p* < 0.05). Protein intensities were variance-stabilising normalised (VSN), differential abundance analysis was performed with linear modelling and empirical Bayes moderation. Two-sided moderated t-tests, Benjamini–Hochberg correction for multiple comparisons. No proteins passed the threshold for multiple-testing correction (adj.*p* < 0.05). **d**,** e** Interaction-confidence landscape and connectivity between the SopB interactome (**c**) and the ADAM17 protein complex. **d** Heatmap of AlphaFold-Multimer–derived interaction confidence for selected pairwise combinations (see Supplementary Fig. 6b). **e** Integrated network connecting SopB interactome and ADAM17 complex using AFM-augmented PPIs (interaction confidence > 0.75) and curated kinase-substrate links from databases. Filtered to retain only direct links or connections via a single intermediate node. **f**,** g** IL-18 (**f**) and IL-6 (**g**) concentration (MFI over non-infected control) in supernatants of stem cell organoid cultures infected with wt or Δ*sopB S*. Typhimurium in the presence or absence of rapamycin, measured by cytometric bead array. Mean ± SD, four independent experiments. **h** IL-18 (MFI over non-infected control) released by intestinal tissue segments from non-infected (*n* = 3), wt (*n* = 7), or Δ*sopB* (*n* = 9) *S*. Typhimurium-infected neonates cultured for 2 h. Cytokine bead array. Mean ± SD, two independent experiments. Statistical analysis by Kruskal-Wallis combined with Dunn’s multiple comparison test (**f**,** g**) and one-way ANOVA Kruskal–Wallis test with Dunn’s post-test (**h**). ns, non-significant.

Our results demonstrated that SopB manipulates cellular processes that promote early innate immune activation, thus explaining the markedly enhanced inflammatory phenotype of Δ*sopB S*. Typhimurium-infected animals. To obtain a more comprehensive view on SopB-manipulated cellular processes in enterocytes, we next performed a phosphoproteome analysis of polarised intestinal epithelial m-IC_cl2_ cells infected for 1 h at a MOI of 10 with wt, Δ*sopB* or Δ*sopB* p*sopB sigE S*. Typhimurium. The full phosphoproteome (5242 protein groups, 44225 peptide isoforms, 15018 phosphosites) was corrected for changes in protein abundance based on a full proteome (4682 protein groups) and analysed for differentially activated core cell signalling pathways. Beside previously described pathways such as PI3K and Akt signalling, we identified activated ERK/MAPK, JAK/STAT, chemokine and mechanistic target of rapamycin (mTOR) pathways in enterocytes upon infection with *S*. Typhimurium representing potential targets for SopB (Fig. 6a). In addition, we analysed the transcriptome of total primary intestinal epithelial cells isolated at day 1 p.i., identifying genes with an increased expression in the presence of SopB as compared to its absence (Supplementary Fig. 1g, blue dots). Enriched GO terms representing significantly upregulated genes in animals infected with wt *S*. Typhimurium compared to animals infected with Δ*sopB S*. Typhimurium suggested an influence of SopB on autophagy and signalling by the mTOR-containing protein complex TORC1 (Fig. 6b). Notably, three differentially expressed genes, *Rnf152* (ring finger protein 152), *Sesn1* (Sestrin 1) and *Sesn3* (Sestrin 3) control the TORC1 signalling pathway consistent with alterations in the autophagocytotic process in the presence of SopB. Finally, we performed an affinity enrichment screen to identify host proteins directly or indirectly interacting with SopB. Here, we used a strain of *S*. Typhimurium chromosomally expressing a triple FLAG-tagged SopB in combination with polarised intestinal epithelial m-IC_cl2_ cells. SopB was purified after 1 h of co-culture to allow SPI1-T3SS-mediated translocation of the tagged SopB into the cell and interaction with host proteins, and co-purified proteins were identified by mass spectrometry. Among other enriched proteins, this screen identified the cytoplasmic linker associated protein (CLASP)1, previously associated with autophagosome trafficking along microtubules^43^, the cytoplasmic casein kinase 1 epsilon (CSNK1E), and the serine/threonine phosphatase 2 A (PP2A) 56 kDa regulatory subunit beta isoform (PPP2R5B), involved in regulation of the major serine/threonine phosphatase PP2A^44^ (Fig. 6c).

Next, we used the AlphaFold Multimer (AFM) pipeline to predict potential interactions between proteins identified in our SopB affinity enrichment screen (SopB interactome, Fig. 6c) and known proteins of the ADAM17 complex, ADAM17, iRhom1/RHBDF1, iRhom2/RHBDF2, and FRMD8. Interestingly, this analysis attributed a high confidence score to interactions between the identified SopB interactome protein CSNK1E and ADAM17 as well as between the identified SopB interactome protein PP2R5B and ADAM17 or iRhom2/RHBDF2 (Fig. 6d, results of all tested pairwise combinations shown in Supplementary Fig. 6c). No direct interaction was detected between SopB and ADAM17 (Supplementary Fig. 6d). Therefore, we next generated an integrated network connecting proteins of the SopB interactome (Fig. 6c) and kinases identified in the phosphoproteome approach (Fig. 6a) together with ADAM17 complex members using an AFM-augmented protein-protein interaction (PPIs) approach with an interaction confidence of > 0.75 and curated kinase-substrate links from public databases. This network visualised some direct and many indirect interactions, consistent with the observed influence of SopB on ADAM17 activity (Fig. 6e).

Besides its effect on ADAM17 activity, the phosphoproteome and transcriptome analyses indicated an influence of SopB on mTOR-regulated autophagy. Secretory autophagy represents a non-canonical secretion pathway and uses the autophagy machinery and autophagosomal transport to release cytokines without a signal peptide, such as IL-18^45,46^. Consistent with enhanced secretory autophagy in intestinal epithelial stem cell organoids infected with Δ*sopB* as compared to wt *S*. Typhimurium, we found that the release of IL-18 but not IL-6 protein was increased (Fig. 6f, g). Importantly, this IL-18 release was reduced to levels observed after wt *S*. Typhimurium infection by pretreatment with the mTOR inhibitor rapamycin (Fig. 6f). Also, the release of IL-18 protein from small intestinal tissue obtained from mice infected with Δ*sopB S*. Typhimurium for 1 day and incubated in vitro for 2 h was significantly higher than the IL-18 release from intestinal tissue of mice infected with wt *S*. Typhimurium (Fig. 6h). In contrast, no influence of SopB on the secretion of the chemokine CXCL2 or release of the innate immune stimulus lipopolysaccharide (LPS) from *S*. Typhimurium-infected epithelial cells was observed (Supplementary Fig. 6e, f). Thus, SopB interferes with cellular processes such as ADAM17-mediated TNFα secretion and the release of cytokines such as IL-18 by secretory autophagy to suppress the early inflammatory response in the neonatal host.

### The N-terminal domain of SopB mediates early immune suppression

**Fig. 7:**
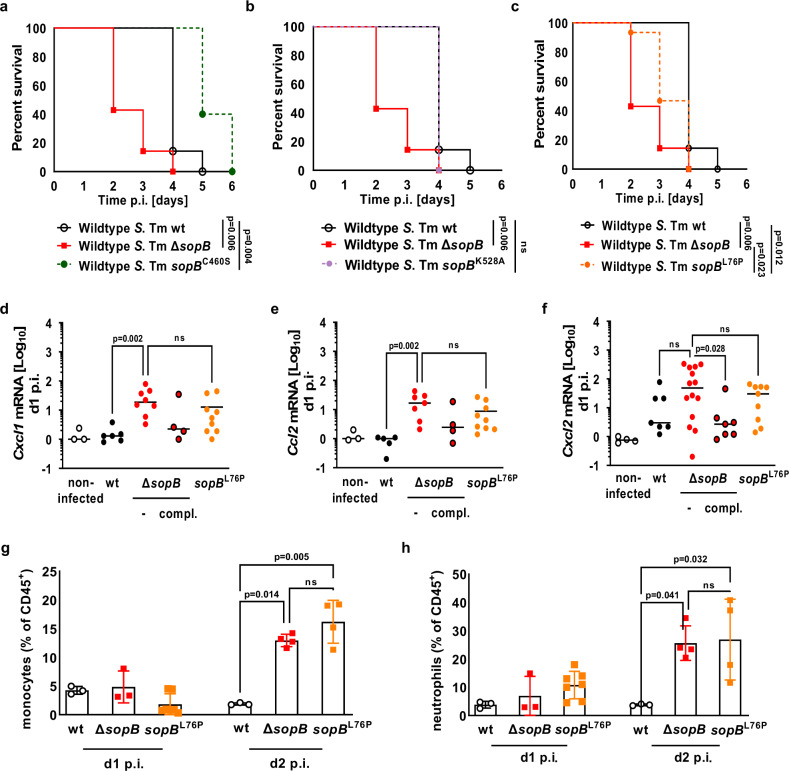
Functional influence of the C- and N-terminal domain on the immune-suppressive phenotype of SopB. **a**–**c** Kaplan-Meier curve of 1-day-old C57BL/6 wildtype mice orally infected with 100 CFU wt (*n* = 7), *sopB* deficient (Δ*sopB*, *n *= 7) *S*. Typhimurium, or S. Typhimurium strains carrying a point mutation in the C-terminal domain of SopB (*sopB*^C460S^, *n* = 5, (**a**); and *sopB*^K528A^, *n* = 4, (**b**)) or N-terminal domain of SopB (*sopB*^L76P^, *n* = 15, (**c**)). Note that the groups of wt and Δ*sopB S*. Typhimurium-infected wildtype mice are identical to Fig. 1A. log-rank (Mantel-Cox) test. **d**–**f** *Cxcl1* (**d**), *Ccl2* (**e**) and *Cxcl2* (**f**) expression in total isolated intestinal epithelial cells isolated at day 1 p.i. from age-matched non-infected mice or mice orally infected with wt (**d**, *n* = 6; **e**, *n* = 5; **f**, *n* = 7), Δ*sopB* (**d**, *n* = 8; **e**, *n* = 7; **f**, *n* = 14), Δ*sopB* p*sopBsigE* (compl., **d**, *n* = 4; **e**, *n* = 4; **f**, *n *= 7) or chromosomally *sopB*^L76P^ carrying *S*. Typhimurium (**d**, **e**, *n* = 3; **f**, *n* = 4). Values were normalised to the house keeping gene *Hprt* and are showed as fold expression over uninfected age-matched control animals (*n* = 3-4). One data point represents one animal from at least two independent experiments, the median. **g**,** h** Flow cytometric analysis of *lamina propria* Ly6C^hi^Ly6G^-^CD11b^+^ MHCII^lo/-^CD45^+^DAPI^-^ monocytes and Ly6G^+^Ly6C^int^CD11b^+^ MHCII^lo/-^CD45^+^DAPI^-^ neutrophils in 1-day-old C57BL/6 wildtype neonates orally infected with 100 CFU wt (*n* = 3 at day 1 and 2 p.i.), *sopB* deficient (Δ*sopB*, *n *= 3 and *n* = 4 at day 1 and 2 p.i., respectively) or *sopB*^L76P^ carrying *S*. Typhimurium (*n* = 7 and *n* = 4 at day 1 and 2 p.i., respectively). **g**, **h** The groups of wt and Δ*sopB*-infected mice are identical to Fig. 2a, b. Mean ± SD. Statistical analysis by Kruskal-Wallis combined with Dunn’s multiple comparison test (**d**–**f**) and two-way ANOVA with Tukey’s multiple comparison test (**g**,** h**). ns, non-significant; *, *p* < 0.05; **, *p* < 0.01.

SopB features functionally distinct N- and C-terminal domains. The C-terminal domain is well known to exert phosphatidylinositol phosphatase and phosphotransferase activity^5,15,23^. Mice infected with *S*. Typhimurium mutants with a chromosomal C460S or K528A mutation in SopB that abolish the C-terminally encoded phosphatase activity exhibited a wildtype-like or even protracted disease course and reduced early mortality (Fig. 7a, b). The N-terminal CRIB like motif (aa117-168) interacts with the small GTPase of the Rho family Cdc42^32,33^, and a L76P point mutation in SopB was shown to ablate this interaction, while preserving the phosphatase activity^31^. Mice infected with *S*. Typhimurium carrying this chromosomal L76P point mutation in the N-terminal domain of SopB exhibited a significantly accelerated disease course and earlier mortality reminiscent of Δ*sopB S*. Typhimurium infected animals (Fig. 7c). The bacterial load in total intestinal epithelial cells and liver tissue did not differ between mice infected with Δ*sopB* and *sopB*^L76P^ *S*. Typhimurium (Supplementary Fig. 7a and b). In addition, infection of neonate mice with either L76P mutant *S*. Typhimurium or Δ*sopB S*. Typhimurium resulted in enhanced expression of the chemokines *Cxcl1, Cxcl2* and *Ccl2* at day 1 p.i. and strongly increased tissue infiltration by neutrophils and monocytes at day 2 p.i. (Fig. 7d–h and Supplementary Fig. 7c). Thus, the observed suppression of early immune activation and tissue inflammation in the neonatal host is mainly mediated by functions of the N-terminal domain of SopB.

## Discussion

Using our neonatal mouse infection model, we here describe the potent immunosuppressive effect of the *S*. Typhimurium SPI1 T3SS translocated effector SopB that completely blunts early chemokine expression and tissue inflammation in vivo. Whereas SopB has been shown to synergise with SopE/SopE_2,_ contributing to actin remodelling, ruffle formation, cell invasion, inhibition of lysosomal fusion, and intracellular growth, our findings show no influence on enterocyte invasion and intraepithelial survival but suggest that its primary role in vivo is the suppression of the early inflammatory host response^4–6,8,9,11,12,14–17,19,21,47,48^. Suppression of the early host response is generally consistent with the previously described cell survival-promoting activity of SopB by Akt stimulation^22–25,27,32,35^. The SopB-induced prolonged host survival most likely increases pathogen shedding, enhancing the likelihood of transmission in accordance with the presence of the *sopB* gene in all clinical isolates^36,49^.

SopB blunted epithelial chemokine expression and immune cell recruitment at day 1 and markedly reduced it on day 2 after oral infection. This is consistent with the early and sustained detection of SopB and its activity in mucosal tissue in vivo^20,24,28^. Previous reports on the immunosuppressive role of SopB using the *S*. Typhimurium strain SL1344 in the adult mouse model have revealed somewhat contradictory results. One study showed no difference in the degree of colonic tissue edema and PMN infiltration comparing Δ*sopB* with wt *S*. Typhimurium infection, whereas another study demonstrated enhanced colitis severity, goblet cell loss and bacterial translocation after infection with Δ*sopB S*. Typhimurium^36,50,51^. Infection of bovine ileal loops with a Δ*sopB* mutant *S*. Dublin strain led to lower tissue infiltration of PMNs^52^. Our own analysis of adult mice after infection with Δ*sopB S*. Typhimurium ATCC14028 did not reveal an immunosuppressive role of SopB. Thus, the immunomodulatory phenotype of SopB appears to be particularly strong in the neonatal infection model, possibly due to age-dependent differences in the mucosal immune homoeostasis or the small intestine as primary target organ in the neonatal infection model^3,4^.

SopB has been well characterised as phosphatidyl-inositol phosphatase mediated by its C-terminal domain^5,8,23^. This activity alters the phosphorylation status of phosphatidyl-inositol residues at the plasma membrane entry site to activate N-WASP and F-actin polymerisation^10,15,53,54^. It has been linked to actin reorganisation and invasion^8,9,15^, membrane fission^6^, inhibition of lysosomal fusion^19^, ion flux alterations and fluid loss^54^, as well as early Akt signalling, and host cell survival^20,22–25,35^. We did not observe impaired enterocyte invasion and, consistently, two point mutations (C460S and K528A) within the C-terminal domain of SopB, known to abolish the phosphatase activity, had no significant influence on the course of the disease in neonatal mice^5,15,23^. More recently, the CRIB-like motif of the N-terminal domain of SopB was shown to interact with Cdc42^30–33^. SopB here acts like a guanosine dissociation inhibitor (GDI), maintaining the small Rho GTPase Cdc42 in an inactive state^33^.

Cdc42 has been shown to control ADAM17 activity in endothelial cells^42^. Consistent with an inhibitory effect of SopB on Cdc42, ADAM17 activity and TNFα release were increased in primary epithelial cells and intestinal tissue from Δ*sopB* but not wt *S*. Typhimurium-infected animals. The potent effect of ADAM17 on TNFα release and local and systemic inflammation in vivo was illustrated using ADAM17-deficient animals^55^. Our affinity enrichment screen of SopB interacting host proteins suggested that SopB does not directly interact with ADAM17 but binds for example, the host protein phosphatase cofactor PPP2R5B and the kinase CSNK1E to act on ADAM17 or the inflammatory ADAM17 co-factor iRhom2/RHBDF2, influencing TNFα release. Clearly, the suggested direct and indirect molecular interactions between molecules of the SopB interactome and the ADAM17 complex warrant further investigations. Nevertheless, this interaction and the early TNFα release in the absence of SopB could explain the requirement of TNFR1 signalling on epithelial chemokine expression at day 1 p.i. with Δ*sopB S*. Typhimurium. In addition, Cdc42 influences transcriptional cytokine regulation and thus the SopB-Cdc42 interaction may also contribute to the lower chemokine transcription at day 2 p.i^56^. Other soluble mediators, such as IL-18, may synergise with TNFα and amplify the early inflammatory reaction in the absence of SopB.

Despite a moderate signal strength, both our phosphoproteome analysis and affinity enrichment screen of SopB interacting host proteins in vitro and the global transcriptome analysis of the intestinal epithelium in vivo indicated a direct or indirect influence of SopB on mTOR, autophagosome function and trafficking. This influence could at least partly be explained by the known interaction of SopB with Cdc42 and the reported stimulatory effect of Cdc42 on mTOR^33,41^. mTOR is a component of the TORC1 complex that inhibits the initiation of autophagy through direct phosphorylation of the Unc-51-like autophagy activating kinase (ULK)1^57,58^. Secretory autophagy is an unconventional protein secretion (UPS) pathway allowing the release of leaderless mediators such as IL-18 and IL-1β^45^. Consistent with a role of mTOR-induced secretory autophagy during early tissue inflammation, we found rapamycin-dependent IL-18 secretion by intestinal epithelial stem cell organoids and enhanced IL-18 release from primary intestinal tissue after Δ*sopB* but not wt *S*. Typhimurium infection.

Together, our findings reveal a pronounced immunosuppressive effect of SopB in the neonatal *S*. Typhimurium infection model in vivo. This activity was independent of the phosphatidyl-inositol phosphatase activity of the C-terminal domain of SopB but required an intact N-terminal domain. Mechanistically, SopB abolished early local tissue inflammation by reducing ADAM17-mediated TNFα release and by inhibiting IL-18 secretion by mTOR-dependent secretory autophagy. SopB thereby delayed disease progression and inflammation-induced mucosal tissue disturbance, prolonging host survival.

## Methods

### Bacterial strains

In this study, wild-type (wt) *Salmonella enterica* subsp. *enterica* serovar Typhimurium (ATCC14028, *S*. Typhimurium), an isogenic Δ*sopB* mutant strain, Δ*sopB* mutant strains complemented with *sopB* (Δ*sopB*, p*sopB*) or *sopB* and its chaperon *sigE* (Δ*sopB*, p*sopBsigE*), an isogenic Δ*sopE*_*2*_ mutant strain^4^, as well as strains carrying chromosomal alleles with point mutations encoding SopB with exchanges in the C-terminal domain at position 460 (C460S, SopB^*C460S*^) or 528 (K528A, SopB^*K528A*^) or in the N-terminal domain in position 76 (L76P, SopB^L76P^) were used (Suppl. Table 1). Low copy number plasmid p4042 has been introduced before^4^ and was shown to restore the function of deleted sopB. pP4042 was used as a template for site-directed mutagenesis using Q5 site-directed mutagenesis kit (NEB) according to the manufacturer’s instructions and primers listed in Supplementary Table 2. The resulting plasmids listed in Supplementary Table 3 were confirmed by DNA sequencing and by Western blot analyses for synthesis of SopB-HA by STM harbouring respective plasmids after subculture in LB for induction of SPI1. Strains expressing HA-tagged mutant *sopB* alleles were generated by λ Red-mediated allelic exchange as described^59^. Briefly, strain MvP2726 was generated by replacing *sopB* by a targeting DNA cassette TC1 generated from pWRG717 using primers sopB In717 For and sopB In717 Rev2 (see Suppl. Table 1, 2). Insertion of the cassette was controlled by check PCR with sopB DelCheck Rev and k1 RedDel. MvP2726 was used as a parental strain for a second λ Red-mediated recombination with TC2 for exchange of the *sopB* locus against wt or mutant alleles. TC2 DNA was generated from plasmids (Supplementary Table 3) with wt or mutant alleles of *sopB*::HA using primers SeqFor and sopB-HA RedIn Rev (Supplementary Table 2). Mutant strains with successful allelic exchange of the *sopB* locus were cured from helper plasmid pWRG730, and synthesis of SopB was controlled by Western blot alleles of *S*. Typhimurium strains after culture under SPI1-inducing conditions. In Western blots, the HA tag was detected using rat anti-HA monoclonal antibodies (clone 3F10, Roche 11867423001). For the generation of a 3xFLAG-tagged allele of *sopB*, the *sopB* locus of strain MD1163 was transferred to ATCC14028 by P22 transduction (Supplementary Table 1).

### Ethics statement

All animal experiments were performed in compliance with the German animal protection law (TierSchG) and approved by the local animal welfare committee (Niedersachsische Landesamt für Verbraucherschutz und Lebensmittelsicherheit Oldenburg, Germany; Landesamt für Natur, Umwelt und Verbraucherschutz, North Rhine Westfalia) under the code 84-02.04.2017.A397 and 84–02.04.2021.A043 including all approved changes.

### In vivo infection experiments

Adult C57BL/6 J wild type mice, *Casp1*^-/-^ (B6. 129S2-Casp1^tm1Flv^/J, stock no.016621), *Asc*^-/-^ mice (B6. 129-Pycard^tm1Vmd^), and *Tnfrsf1a*^-/-^ (Tnfrsf1a^tm1MAK^; stock 002818) were obtained from Jackson Laboratory (Bar Harbour, USA) and bred locally at University Hospital RWTH Aachen under SPF conditions. *Mlkl*^-/-^ (BV6. 129-Mlkl^tm1/J^), and *Casp8*^*ΔIEC*^ (B6. 129-Casp8^tm1Hed^/J; stock 027002) mice were provided by James Murphy (Walter and Eliza Hall Institute of Medical Research, Australia) and Claudia Günther (University Hospital Erlangen, Germany) and bred locally at University Hospital RWTH Aachen under SPF conditions. Overnight bacterial cultures grown on a shaker in Luria Bertani (LB) were diluted 1:10 and incubated at 37 °C on a wheel (22 rpm) under mild aeration to induce SPI1 T3SS activity until reaching the logarithmic phase (OD_600_: 0.5) as described^3,60^. Bacteria were washed and diluted to obtain the appropriate inoculum in PBS. One-day-old animals were orally infected with 100 CFU *S*. Typhimurium. 9-week-old adult female mice were pretreated with streptomycin (20 mg) administered by intragastric gavage one day prior to oral infection with 10^7^ CFU *S*. Typhimurium, as previously described^60^. At the indicated time point postinfection (p.i.), liver, spleen and mesenteric lymph nodes (MLN), small intestine, as well as blood samples were collected. Viable counts were obtained by serial dilution and plating of homogenised tissue on LB agar plates supplemented with the appropriate antibiotic(s). Small intestinal tissues were collected, fixed in 4% paraformaldehyde (PFA) for histological analysis or processed for total tissue expression, respectively. For ex vivo tissue cytokine secretion, small intestines were longitudinally opened and sectioned into smaller pieces. Tissue pieces were incubated in 100 µL RPMI medium supplemented with 10% fetal calf serum (FCS) at 37 °C in a 5% CO_2_ humidified atmosphere. After 2 h, 50 µl supernatant was collected and analysed.

### Gene expression analysis

RNA was isolated from the epithelial cell pellet or homogenised intestinal tissue using TRIzol® according to the manufacturer’s recommendations. The RNA concentration was determined using a NanoDrop 1000 spectrophotometer (Thermo Scientific). First-strand complementary DNA (cDNA) was synthesised from 5 µg total RNA using Oligo-dT primers, 5X PCR buffer, dNTP, RevertAid reverse transcriptase and RiboLock RNase inhibitor (ThermoFisher Scientific). RT-PCR was performed using Taqman technology with an absolute QPCR ROX mix (Thermo Scientific). Taqman probes *Hprt* (house-keeping gene, Mm00446968_m1), *Cxcl1* (Mm04207460_m1), *Cxcl2* (Mm00436450_m1), *Cxcl5* (Mm00436451_g1), *Ccl2* (Mm00441242_m1), *Tnf* (Mm00443258_m1), *Reg3g* (Mm00441128_g1), *Bcl2* (Mm01302952_g1) or *Nos2* (Mm00440502_m1) from ThermoFisher Scientific were used. Results were calculated by the 2^−ΔΔCt^ method. Values were normalised to the *Hprt* housekeeping gene and are presented as fold induction over age-matched healthy controls.

### RNA Seq and transcriptome analysis

RNA was prepared from primary, freshly isolated intestinal epithelial cells obtained from age-matched uninfected mice, or mice infected with wt or Δ*sopB S*. Typhimurium at day 1 p.i., using TRIzol®. Libraries were prepared with the QuantSeq 3’mRNA-Seq v2 Library Prep Kit FWD with UDIs (Lexogen), using an input of 125 ng, and were sequenced in single end mode (read 1: 75 cycles, index 1: 12 cycles, index 2: 12 cycles, read 2: 0 cycles) on a NovaSeq 6000 (Illumina), using a NovaSeq 6000 SP Reagent Kit v1.5 (100 cycles) (Illumina). Raw sequencing reads were trimmed using Cutadapt v4.9 with the following parameters: -j 16 -a “poly A = A(20)” --quality-cutoff 20 -m 20 -u 12, to remove poly-A tails, low-quality bases, and adaptor sequences. A genome index was generated using STAR v2.7.11b in genome generation mode (--sjdbOverhang 63) based on the GRCm39.112 mouse reference genome. Trimmed reads were then aligned to the genome index using STAR with parameters --outSAMtype BAM SortedByCoordinate and --quantMode GeneCounts to produce BAM files. Gene-level count matrices were generated using the featureCounts function from the Rsubread package (v2.14.2). Differential gene expression analysis was performed using DESeq2 v1.44.0, including only genes with a total count of at least 60 across all samples. GO enrichment analysis was conducted using the clusterProfiler package (v4.12.6) with the org.Mm.eg.db database. Multiple testing correction was applied using the Benjamini-Hochberg method. GSEA was performed with the Python gseapy package (v1.1.9) using genes from the mmu04668 KEGG pathway.

### Cytokine, chemokine and endotoxin quantification

Cytokine and chemokine levels, including the concentrations of TNF and IL-18 in the medium supernatants and serum samples, were measured using the LEGENDplex^TM^ Mouse Virus Response Panel (BioLegend, Cat No 740622) and LEGENDplex™ Mouse M1 Macrophage Panel (BioLegend, Cat. Nr 740848) according to the manufacturer’s protocol. Samples were measured using a BD FACS Canto II and analysed with the LEGENDplex™ Data Analysis Software Suite (Qonit). Results are expressed as picograms per millilitre. The heat map was generated using Heatmapper (http://www.heatmapper.ca)^61^. CXCL2 secretion by m-IC_cl2_ cells was quantified using a CXCL2 ELISA from Biosite (Cat. No.: PPE 21335). The endotoxin concentration in cell culture medium was measured using the Kinetic-QCL^TM^ Kinetic chromogenic LAL assay (Lonza, Cat. No.: 50-650).

### Intestinal epithelial and immune cell isolation and analysis

Primary small intestinal epithelial cells were isolated as previously described^3^. Briefly, epithelial cells were detached from the underlying tissue in 30 mM EDTA/PBS with strong shaking. To analyse the number of intraepithelial *S*. Typhimurium, one fraction of epithelial cells was treated with 100 μg/mL gentamicin for 1 h at room temperature, washed in PBS and plated in serial dilutions on selective LB agar plates. The other fraction of epithelial cells was stored in Trizol at − 80 °C for subsequent gene expression analysis. For the isolation of immune cells, Peyers patches and feces were removed, the intestine was opened longitudinally and transferred into 20 ml of HBSS/3% FBS with 2 mM EDTA. The intestine was shaken twice at 150 rpm at 37 °C for 20 min. After the second incubation, the intestine was rinsed with PBS to remove the EDTA. The remaining intestine was enzymatically digested in RPMI (Gibco) containing 30 µg/ml Liberase^TM^ (Roche) and 100 µg/ml DNase (Roche) for 45 min shaking at 37 °C. Tissue pieces were filtered through a 100 µm nylon cell strainer (BD) to obtain a single cell suspension. Immune cells were separated using a Percoll gradient by centrifugation at 700 x *g* for 20 min. at room temperature. Cells were stained using the following antibodies CD45-FITC (Clone 30-F11), Ly6C-PerCPCy5.5 (Clone HK1.4), Ly6G-PE (Clone 1A8), Ly6G-Spark NIR 685 (Clone 1A8), Ly6C-BV711 (HK1.4), CD11b-APC Cy7 (Clone N418), CD11b-BUV 395 (Clone M1/70), CD64-APC (Clone X54-5/7.1), CD64-PE Dazzle (Clone X54-5/7.1), MHCII-AF488 (Clone M5/114.15.2), MHCII-BV510 (Clone M5/114.15.2), PDL1-PE (Clone 10 F.952), SiglecF-APCR700 (Clone 90/CD38; BD), Epcam-BV421 (Clone G8.8), CD3-FITC (Clone 17A2), CD19-FITC (Clone 6D5), (Biolegend) and DAPI (Roth) for subsequent analytical flow cytometry. Data were acquired with a BD FACS Canto II and analysed with FlowJo X. For FACS sorting, approximately 3-6 million monocytes, macrophages, and neutrophils were sorted by BD Biosciences FACS Aria Fusion Sorter and directly collected into RNA lysis Buffer (QIAGEN RNeasy Micro Kit QIAGEN). Total RNA of each immune cell population was extracted using QIAGEN RNeasy Micro Kit following the manufacturer’s instructions.

### Ex vivo stimulation of isolated immune cells

Immune cells were collected, washed in 3% FCS/PBS and counted. 10^6^ cells were cultured in 500 µL Iscove’s modified Dulbecco’s medium (Thermo Fisher Scientific) supplemented with 10% FBS, with/without 1 µL of the cell activation cocktail (phorbol myristate acetate (PMA) and ionomycin (I), Biolegend, Cat. No.: 423302). After incubating the cells at 37 °C in a 5% CO_2_ humidified atmosphere for 1 h, 5 µg/ml brefeldin A (BFA, Biolegend, Cat. No.: 420601) was added. After 3 more hours of re-stimulation, cells were collected, washed once with 3% FCS/PBS, and resuspended in 3% FCS/PBS. Cells were then harvested and stained with the following antibodies (Biolegend): CD45-APCR700 (Clone 30-F11; BD Biosciences), CD3-APCFire750 (Clone 17A2), PDL1-APC (Clone 10 F.952), SiglecF-BB515 (Clone E50-2440; BD Biosciences), CD11c-BUV737 (Clone N418: BD Biosciences), CD64-PEDazzle (Clone 90/CD38), CD11b-BV786 (Clone M1/70), F4/80-PECy5 (Clone BM8), Epcam-BV421 (Clone G8.8), MHCII-BV510 (Clone M5/114.15.2), Ly6C-PerCP-Cy5.5 (Clone HK1.4), Ly6G-BV711 (Clone 1A8), CD80-BUV 805 (Clone 16-10A1; BD Biosciences), CD19-PECy7 (Clone 6D5) for 20 min at 4 °C and 30 min with Zombie UV at 4 °C (Cat. No.: 423107, BioLegend). Stained cells were fixed and permeabilised (BD Cytofix/Cytoperm™, Cat. No. 554722 according to the manufacturer’s instructions) prior to intracellular cytokine staining with a TNFα-PE (Clone MP6-XT22) antibody overnight at a 1:500 dilution (Biolegend). Data were acquired on a Cytek Aurora flow cytometer and analysed with FlowJo X.

### Immunostaining

Fixed small intestinal tissues were embedded in paraffin or OCT. 4 μm thick sections were deparaffinised and rehydrated. Slides were stained with haematoxylin and eosin Y solution for H & E staining and observed under a Zeiss Axio Imager M2 light microscope. The thickness of the *lamina propria* as a measure of the tissue oedema was measured using the ZEN 3.4 imaging software. For immunofluorescence staining, tissue sections were blocked with 10% normal donkey serum/5% BSA/PBS. Rabbit anti-Ki67 (Ab15580, Abcam), mouse anti-E-cadherin (610182, BD Transduction Laboratories), rat anti-PMN (Ly6-6B2, SeroTec), rabbit anti-Muc2 (GTX100664, BIOZOL), and rabbit monoclonal anti-ADAM17 (JM10-35, Invitrogen) were used at the appropriate dilution, followed by the fluorophore-conjugated donkey secondary antibody (Jackson ImmunoResearch). AF647-conjugated wheat germ agglutinin (WGA, Vector Laboratories, FL1021) was used to visualise the mucus layer. The in situ cell death detection Kit (Roche) was used following the manufacturer’s instructions to detect TUNEL-positive cells. Slides were counterstained with DAPI (Vector Laboratories) and images were taken using a Zeiss ApoTome.2 system microscope connected to an Axiocam 506 digital camera (Zeiss). The thickness of the *lamina propria* and the fraction of the ADAM17^+^ intestinal epithelial apical surface were quantified using the ZEN 3.4 imaging software.

### Neonatal intestinal epithelial stem cell organoid culture

Neonatal intestinal epithelial stem cell organoids (spheroids) were prepared according to established protocols and grown as cell monolayers^39,62^. Briefly, small intestinal crypts were isolated by incubation at 4 °C in PBS containing 2 mM EDTA for 5 min. from total neonatal small intestine tissue seeded in Matrigel (356231; BD Biosciences) into 48-well plates (20 μl of Matrigel per well). Matrigel was polymerised at 37 °C for 15 min and 250 μl of ENR basal culture medium (advanced DMEM/F12 medium [12634-028; Gibco] supplemented with penicillin/ streptomycin [15140-122; Gibco], 0.01 M HEPES [15630-056; Gibco], 1 × Glutamax [35050-038; Gibco], 1 × N2 [17502-048; Gibco], 1 × B27 [17504-044; Gibco], 500 mM N-acetylcysteine [A9165; Sigma-Aldrich], 50 µg/ml mouse EGF [PMG8045; Gibco], 100 µg/ml mouse noggin [250-38; PeproTech], and 10% of R-spondin conditioned medium purified from the supernatant of stably transfected HEK293T cells) was added to each well. Medium change was performed every 3 days, and organoids were passaged 1:5 after 7 days. To obtain cell monolayers, 4-day-old spherical organoids were trypsinised with TrypLE Express (12605-010; Gibco), filtered and washed by centrifugation. Cells were resuspended in ENRWY medium (ENR medium containing 50% Wnt3a conditioned medium) purified from the supernatant of stably L-Wnt-3A expressing HEK cells and 10 µM RhoK inhibitor Y-27632 (M20999; AbMole Bioscience). 200 μl of the cell suspension was added to each well of a 48-well cell culture plates followed by a 1 min centrifugation step to promote attachment to the Matrigel layer. After 16–18 h, non-adherent cells were removed, and the cells were incubated again at 37 °C for 24 h. Dead cells were removed by washing with prewarmed PBS. R-spondin-producing and Wnt3a-producing HEK293T cells were kindly provided by Calvin Kuo (Stanford University, Stanford, CA, USA) and Sina Bartfeld (Berlin Technical University, Berlin, Germany), respectively. Confluent cell monolayers were infected with *S*. Typhimurium at a multiplicity of infection (MOI) of 10:1 for 1 h. Monolayers were washed three times with warm PBS and supplemented with pre-warmed ENRWY media containing 100 μg/mL gentamicin (Sigma) for 1 h at 37 °C to remove extracellular bacteria. Infected monolayers were washed again three times in warm PBS and lysed. The number of intracellular bacteria was determined by serial dilution and plating on selective LB agar plates. The invasion rate was calculated as (number of intracellular bacteria/number of administered bacteria) X 100[%].

To evaluate the role of secretory autophagy for cytokine release by intestinal stem cell organoids, confluent 2D organoids were washed once with warm PBS prior to infection with *S*. Typhimurium, to remove dead cells. Cells were left untreated or treated with 200 nM rapamycin for 1 h. The supernatant was then removed, and the fresh ENRWY media containing 20 nM rapamycin was added for subsequent steps. Wt or Δ*sopB S*. Typhimurium was added to stem cell organoid cells grown to confluency at a multiplicity of infection (MOI) of 10:1. After 1 h of infection, infected monolayers were washed three times with warm PBS and supplemented with pre-warmed ENRWY media containing 100 μg/mL gentamicin (Sigma) for 1 h at 37 °C to remove extracellular bacteria. Supernatants were analysed using a cytometric bead array (Cytometric Bead Array Kit, BioLegend) according to the instructions of the manufacturer.

### ADAM17 activity assay

Isolated intestinal epithelial cells were washed twice with PBS by centrifugation at 300 x *g* for 5 min at 4 °C. The pellets were resuspended in PBS and transferred to a black 96-well plate suitable for fluorescence measurements. The pellets were incubated at 37 °C in a humidified 5% CO_2_ incubator for 30, 60, 90, 120 min and 180 min in the presence of 10 µM ADAM17/TACE substrate (Sigma, Cat. Nr: 616407), with or without 10 µM ADAM17/TACE inhibitor (Sigma, GW-3333), in a total volume of 50 µl PBS. ADAM17 enzymatic activity was quantified at the indicated time points by measuring fluorescence intensity at Ex/Em = 320 nm/420 nm using a fluorescence microplate reader (SpectraMax i3, ROM v1.4 b18). At the end of the incubation period, IEC pellets were lysed using 0.1% Triton X-100 (Cayman, item: 601172) and total protein concentrations of the lysates were determined using the Bradford assay (Bio-Rad) following the manufacturer’s instructions.

### m-IC_cl2_ co-culture experiments

m-IC_cl2_ cells were seeded onto polyethylene terephthalate (PET) ThinCert™ transwell inserts with a pore size of 3μm (Greiner Bio-One, Kremsmünster, Austria) and grown to a confluent monolayer of polarised epithelial cells in m-IC_cl2_ medium supplemented with 2% heat-inactivated FCS for 10–12 days with medium changes three times per week^63^. The integrity of the epithelial monolayer was assessed by monitoring the transepithelial electrical resistance (TEER). Wild-type (wt) and Δ*sopB S*. Typhimurium were added to the apical compartment at a multiplicity of infection (MOI) of 10:1. To promote host cell contact, plates were centrifuged at 300 x *g* for 5 min. After 1 h incubation at 37 °C, cell monolayers were washed with warm PBS and incubated in fresh cell culture medium supplemented with 100 μg/ml gentamicin (Sigma, Cat. No.: 1405-41-0) for 1 h to remove extracellular bacteria. After 1 h, the medium was replaced by fresh medium supplemented with 20 μg/ml gentamicin. Non-infected m-IC_cl2_ cells were treated similarly.

### Proteomics and phosphoproteomics

m-IC_cl2_ cells were grown to confluency and polarised for 7 days. Cells were infected at a MOI of 10:1 for 1 h, non-infected cells served as a control. Cells were lysed in 1% Triton X-100, 150 mM NaCl, 50 mM Tris-HCl (pH 7.4), 0.5% sodium deoxycholate, and 0.1% SDS, including Roche’s complete proteinase and phosphatase inhibitors. Experiments were conducted in 4 biological replicates. For the full proteome, 30 µg protein from each replicate was used and prepared by protein clean up and enzymatic cleavage using a paramagnetic bead approach as described previously^64^. Briefly, the volume of protein samples was adjusted to 50 μL with 100 mM TEAB (Tetraethylammonium tetrahydroborate, Sigma-Aldrich, USA), followed by reduction with 5 μL 200 mM TCEP (Tris(2-carboxyethyl)phosphine hydrochloride, Sigma-Aldrich, USA) in 100 mM TEAB for 1 h at 55 °C. Subsequently, 5 μL 375 mM iodoacetamide (Merck KGaA, Germany) in 100 mM TEAB was added and incubated for 30 min at room temperature in the dark. 2 μL SP3 beads per sample were washed with water three times, with subsequent addition of the sample. After protein binding to the beads, the supernatant was discarded. Then, the beads were washed twice with 200 μL 70 % (v/v) ethanol, and once with 200 pure ACN. Finally, the proteins were digested with trypsin (Promega, Germany) in a ratio of 1:50 for 16 h at 37 °C. Subsequently, a peptide clean-up was conducted. Therefore, ACN was added to each sample to reach a final organic content higher than 95 % (v/v). After peptide binding to the beads, the samples were washed with pure ACN on the magnetic rack.

Peptides were eluted in two fractions, the first one with 87% acetonitrile in 10 mM ammonium formate (pH 10, Sigma Aldrich), and the second one with 2% dimethylsulfoxide (DMSO, Sigma Aldrich). Both fractions were analysed using liquid chromatography (LC) coupled to a mass spectrometer (MS). In detail, the peptides were separated on a nano-UPLC system (Ultimate 3000, Dionex, USA) with a trapping column (flow rate 5 µl/min, Acclaim PepMap 100 C18, 3 µm, nanoViper, 75 µm × 5 cm, Thermo Fisher, Germany) and an analytical column (flow rate 0.3 µl/min, Acclaim PepMap 100 C18, 3 µm, nanoViper, 75 µm × 25 cm, Thermo Fisher, Germany) using a 160 min non-linear gradient as described in ref. ^64^. The nano-UPLC system was coupled to the MS (QExactive HF, Thermo Scientific, USA) via a chip-based ESI source (Nanomate, Advion, USA). The only difference compared to the previously described workflow^64^ was that not the top 10 but the top 15 precursor ions were subjected to MS/MS analysis. The obtained raw data were processed against the UniProtKB reference proteome of *Mus musculus* (March, 18, 2023), using Proteome Discoverer 2.5 and the following parameters: carbamidomethylation as fixed modifications, oxidation of methionine and acetylation of the protein N-terminus as variable modifications. This workflow resulted in information on 4682 proteins.

For the phosphoproteome, 600 µg protein were used, followed by protein clean up and enzymatic cleavage using a paramagnetic bead approach as described above and previously^65^. Peptides were eluted after the peptide clean-up in water, resulting in one fraction. After elution, a two-step enrichment of phosphorylated peptides using the HighSelect™ TiO2 Phosphopeptide Enrichment Kit (Thermo Scientific, USA) and the High-Select™ Fe-NTA Phosphopeptide Enrichment Kit (Thermo Scientific, USA) was performed as described before^65^. Enriched phosphorylated peptide samples were analysed using the same LC-MS/MS system as the full proteome samples with a 160 min non-linear gradient and with adjusted MS parameters: precursors between 350 m/z and 1550 m/z were detected at a resolution of 120 K. MS1 automatic gain control (AGC) target was set to 3e6, with a maximum injection time of 150 ms. The top 15 precursors were isolated using a window of 0.7 Th, with MS2 AGC target 2e5 and a maximum injection time 150 ms. The normalised collision energy (NCE) was 34, fixed first mass 120 m/z, and MS2 resolution 60 K. A dynamic exclusion of 45 s was used. The obtained raw data were processed against the same UniProtKB reference proteome as the proteome, using Proteome Discoverer 2.5 and the following parameters: phosphorylation on serine, threonine, or tyrosine, oxidation of methionine, and acetylation of the protein N-terminus as variable modifications. This workflow resulted in information on 5242 proteins, 44225 peptide isoforms, and 15018 phosphosites.

For the identification of regulated proteins/phosphosites and enrichment analysis, the data were first filtered for proteins and phosphosites identified at least in three replicates, followed by log2-transformation and median-normalisation. The average Log2(FCs) were calculated, and regulated proteins and phosphosites were determined using the Student’s *t* test conducted in R Studio 3.6.1. Obtained *p*-values were adjusted for multiple testing, according to Benjamini & Hochberg. Proteins and sites were considered significantly regulated with FDR ≤ 0.05. Enrichment analyses were conducted with regulated proteins or regulated phosphosites (FDR ≤ 0.05) using Ingenuity Pathway Analysis (IPA, Qiagen). Enrichment p-values were calculated with the right-tailored Fisher’s exact test and adjusted for multiple testing, according to Benjamin & Hochberg. Pathways were considered significantly enriched with FDR ≤ 0.05.

### Affinity enrichment of SopB-associated host proteins

Polarised and confluent cell layers of m-IC_cl2_ cells were infected with *S*. Typhimurium chromosomally carrying a triple *Flag*-tagged *sopB* construct at a multiplicity of infection (MOI) of 10:1, or an untagged wildtype strain as a background control. Plates were centrifuged at 1,200 rpm for 5 min to initiate host cell contact. After 1 h incubation at 37 °C, cell monolayers were washed with cold PBS and lysed in Pierce RIPA buffer (Thermo Scientific) supplemented with cOmpleteTM protease inhibitor tablet (Roche) and PhosSTOP (Roche). The cell lysate was harvested by centrifugation at 13,000 rpm for 20 min at 4 °C and mixed with 30 µL of washed anti-FLAG® M2 Affinity Gel (Sigma, A2220). The mixture was rotated at 4 °C for 4 h to allow binding. Unbound proteins were washed away with 0.01% PBS-Triton X-100 buffer at 5000 × *g* for 5 min. at 4 °C. The bound proteins were eluted using 150 µg/mL FLAG peptide (Waters) prepared in 0.05% RapiGest (Waters). The eluted proteins were resuspended in 50 mM HEPES (pH 8) containing 1% SDS, 40 mM 2-chloroacetamide, and 10 mM TCEP, then incubated at 95 °C for 5 min to facilitate reduction and alkylation. Nucleic acids were digested with Benzonase (2.8 U/Sample) at 37 °C for 30 min. Samples were processed for mass spectrometry using a modified SP3 protocol^66^. Proteins were digested with Trypsin and LysC at 37 °C for 14 h. Peptides were labelled using 6plex TMT (Thermo Fisher) following Zecha et al.^67^. A total of 6 samples (3 test samples and 3 background controls from three biologically independent experiments performed on separate days) were pooled and desalted using a Waters OASIS HLB μElution Plate. LC-MS/MS was performed on an UltiMate 3000 RSLCnano coupled to an Orbitrap Exploris 480 mass spectrometer (Thermo Fisher). Peptides were separated on a C18 analytical column (IonOpticks) over 160 min (1–40% B, 0.25 μl/min). MS1 spectra (400–1,600 m/z) were acquired at 120,000 resolution; the top N precursors (charge 2–5, cycle time 3 s) were fragmented (NCE 32) and analysed at 15,000 resolution.

Raw files were converted to mzML (MSConvert v3.0.23129) format prior to database searching. Peptide and protein identification was performed using the TMT10 workflow implemented in MSFragger (v3.8) through FragPipe (v20.0). Spectra were searched against the UniProt Mus musculus reference proteome (UP000000589, downloaded April 8, 2025) and *Salmonella enterica* serovar Typhimurium strain 14028S proteome (UP000002695, downloaded April 8, 2025), supplemented with common contaminant proteins and reverse decoy sequences. Searches were performed using strict trypsin specificity, allowing up to two missed cleavages and a minimum peptide length of seven amino acids. Precursor and fragment mass tolerances were set to − 20 to + 20 ppm and 20 ppm, respectively. Carbamidomethylation of cysteine and TMT labelling of lysine residues were specified as fixed modifications. Oxidation of methionine (maximum three occurrences per peptide), protein N-terminal acetylation, and TMT labelling of peptide N-termini were included as variable modifications. Peptide-spectrum matches, peptides, and proteins were filtered at a 1% false discovery rate. Proteins identified by at least one unique peptide were retained for downstream analysis.

Downstream analyses, including normalisation (VSN) and differential expression, were conducted in R (RStudio v2021.09.2) using limma (v3.54.2), vsn (v3.66.0), and the tidyverse suite (dplyr v1.1.1, ggplot2 v3.4.2). Differential abundance was assessed using linear modelling with empirical Bayes moderation implemented in limma. Statistical significance was determined using two-sided moderated *t* tests, and *p*-values were adjusted for multiple testing using the method of Benjamini–Hochberg.

### Immunoprecipitation

Affinity purification of SopB-interacting proteins was performed as described above. For immunoblotting of ADAM17, protein eluted from the anti-FLAG® M2 Affinity Gel, total m-IC_cl2_ cell lysate, and *S*. Typhimurium *sopB*::3xFLAG lysate were incubated at 95 °C for 10 min with 4 x SDS loading dye, loaded on a 10% SDS-PAGE, and run at 120 V for 60 min. Proteins were transferred to a nitrocellulose membrane at 250 mA for 90 min. The membrane was blocked with 5% milk-TBS-T for 1 h at room temperature and incubated with anti-FLAG® M2 antibody diluted 1:2000 (F1804, Sigma) or anti-ADAM17 antibody diluted 1:2000 (JM10-35, Invitrogen) overnight at 4 °C. After washing three times with TBS-T, the membrane was incubated with the secondary antibody conjugated to HRP for 1 h at room temperature in 5% milk-TBS-T. Finally, the washed membrane was incubated with SuperSignal West Femto Maximum Sensitivity Substrate (Thermo Scientific) and scanned using a C-DiGit Blot Scanner (LICORbio).

### Network analysis

To identify potential interactions between SopB affinity enriched host proteins and the ADAM17 complex, consisting of ADAM17, iRhom1/RHBDF1, iRhom2/RHBDF2, and FRMD8, the curated interactomics database BioGRID was queried. To infer candidate–complex connectivity via direct interactions and shared interactors, we performed high-throughput AlphaFold-Multimer (AFM) prediction and interaction confidence scoring^68,69^. Protein sequences were retrieved via the UniProt API^70^ using custom Python scripts (Python v3.11.0). Proteins larger than 650 residues were segmented into prediction units using AlphaFold DB–derived pLDDT and PAE profiles to place boundaries in low-confidence regions between structural domains. All pairwise combinations were subjected to AFM prediction^71^ using MSAs generated with MMseqs2^72^. For each protein pair, AFM produced five models, which were ranked using the actifpTM score^73^. A custom pipeline extracted Cα coordinates from both chains and defined interface residue pairs as positions within 10 Å. For each model, the mean interface PAE (iPAE) across all interface residues was computed and transformed to a normalised score by mapping iPAE ≤ 5 Å to 1, iPAE > 15 Å to 0, and linearly scaling intermediate values (5–15 Å). The model interaction score was calculated as the arithmetic mean of actifpTM and iPAE. Pairwise confidence was reported as the mean across the five AFM models (reflecting consensus across AFM weight sets), and high-confidence PPIs for network inclusion were defined as mean interaction confidence > 0.75. In addition, connectivity was augmented using a directed kinase-to-substrate reference database assembled by integrating curated resources: OmniPath^74^, HPRD^75^, PhosphoSitePlus^76^, Phospho.ELM^77^, Reactome^78^ and DEPOP^79^. Network construction and visualisation were performed with custom Python scripts; scripts are available upon reasonable request or, after publication, via https://github.com/Clusterbiology.

### Statistics

Measurements were taken from distinct samples. Survival was analysed by log-rank (Mantel -Cox) test. The non-parametric Mann-Whitney test was used for the comparative analysis of two groups. The Kruskal-Wallis, combined with Dunn’s multiple comparison test, was employed for the statistical analysis of more than two groups. If data were normally distributed as confirmed using the Shapiro-Wilk test, the student’s *t* test (two groups) or the one-way ANOVA test with Tukey’s posttest (more than two groups) was used. Two-way ANOVA with Sidak or Tukey’s multiple comparison test was employed for the statistical analysis of two groups that have been split on two independent variables. Graphpad Prism Software 10 was used for statistical evaluation. Differences were considered significant at *p* < 0.05, *; *p* < 0.01, **; *p* < 0.001, ***; and *p* < 0.0001, ****.

### Reporting summary

Further information on research design is available in the Nature Portfolio Reporting Summary linked to this article.

## Supplementary information

Supplementary information

Reporting Summary

Transparent Peer Review file

## Source data

Source data file

## Data Availability

The mass spectrometry proteomics data have been deposited to the ProteomeXchange Consortium (https://www.ebi.ac.uk/pride/archive) via the PRIDE partner repository with the dataset identifier PXD046081 (phosphoproteome approach) and the dataset identifier PXD079152 (affinity enrichment approach)^80^. Transcriptomic data have been deposited at GEO with the accession number GSE300992 (GSM9073322-GSM9073332) and GSE326693. Both datasets are openly accessible. Source data are provided in this paper.

## Code availability

The network construction and visualisation Python scripts are available via https://github.com/Clusterbiology, repository name: aiPAE_calculator, 10.5281/zenodo.20417157 (2026).
