# Supplementary material for: Salmonella SopB suppresses post-transcriptionally regulated cytokine release to reduce early tissue inflammation and delay disease progression
Source: Nat Commun. 2026 Jul 6;17:5884. doi: 10.1038/s41467-026-74942-9 (PMC13338139; doi:10.1038/s41467-026-74942-9)
Supplement: Supplementary file 1 — Supplementary information [file 41467_2026_74942_MOESM1_ESM.pdf]

Supplementary Information

Salmonella SopB suppresses post-transcriptionally regulated cytokine release to reduce early tissue inflammation and delay disease progression

Nour Diab[1], Chiun Huei Yong[1], Eva-Lena Stange[1], Marlène Birk[1], Matthias Schmitz[1], Stefan Düsterhöft[2§], Jonas Pes[1], Kira Noemi Ferle[1], Isabel Karkossa[3], Kristin Schubert[3], Jörg Deiwick[4], Mihael Vucur[5], Tom Luedde[5], Natalia Torow[1,6], Andreas Ludwig[2], Aline Dupont[1], Joel Selkrig[1], Martin von Bergen[3], Michael Hensel[4], Kaiyi Zhang[1*], Mathias W. Hornef[1,7*]

Supplementary Figures

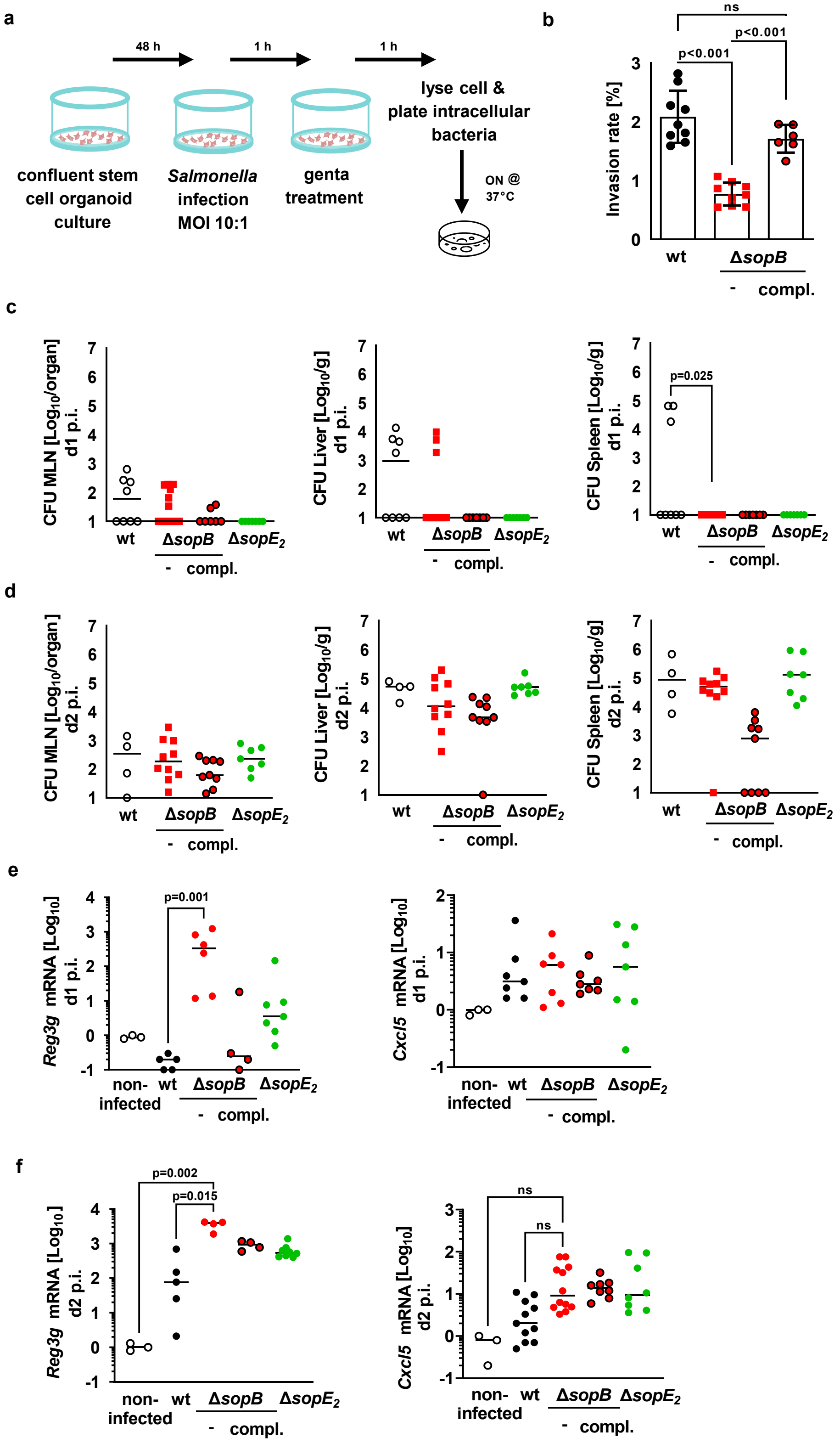

a
48 h
1 h
1 h
confluent stem cell organoid culture
Salmonella infection MOI 10:1
genta treatment
lyse cell & plate intracellular bacteria
ON @ 37°C
b
Invasion rate [%]
ns
p<0.001
p<0.001
wt
ΔsopB
-
compl.
c
CFU MLN [Log10/organ] d1 p.i.
CFU Liver [Log10/g] d1 p.i.
CFU Spleen [Log10/g] d1 p.i.
p=0.025
wt
ΔsopB
ΔsopE2
-
compl.
d
CFU MLN [Log10/organ] d2 p.i.
CFU Liver [Log10/g] d2 p.i.
CFU Spleen [Log10/g] d2 p.i.
e
Reg3g mRNA [Log10] d1 p.i.
p=0.001
Cxcl5 mRNA [Log10] d1 p.i.
non-infected
f
Reg3g mRNA [Log10] d2 p.i.
p=0.002
p=0.015
Cxcl5 mRNA [Log10] d2 p.i.
ns
ns

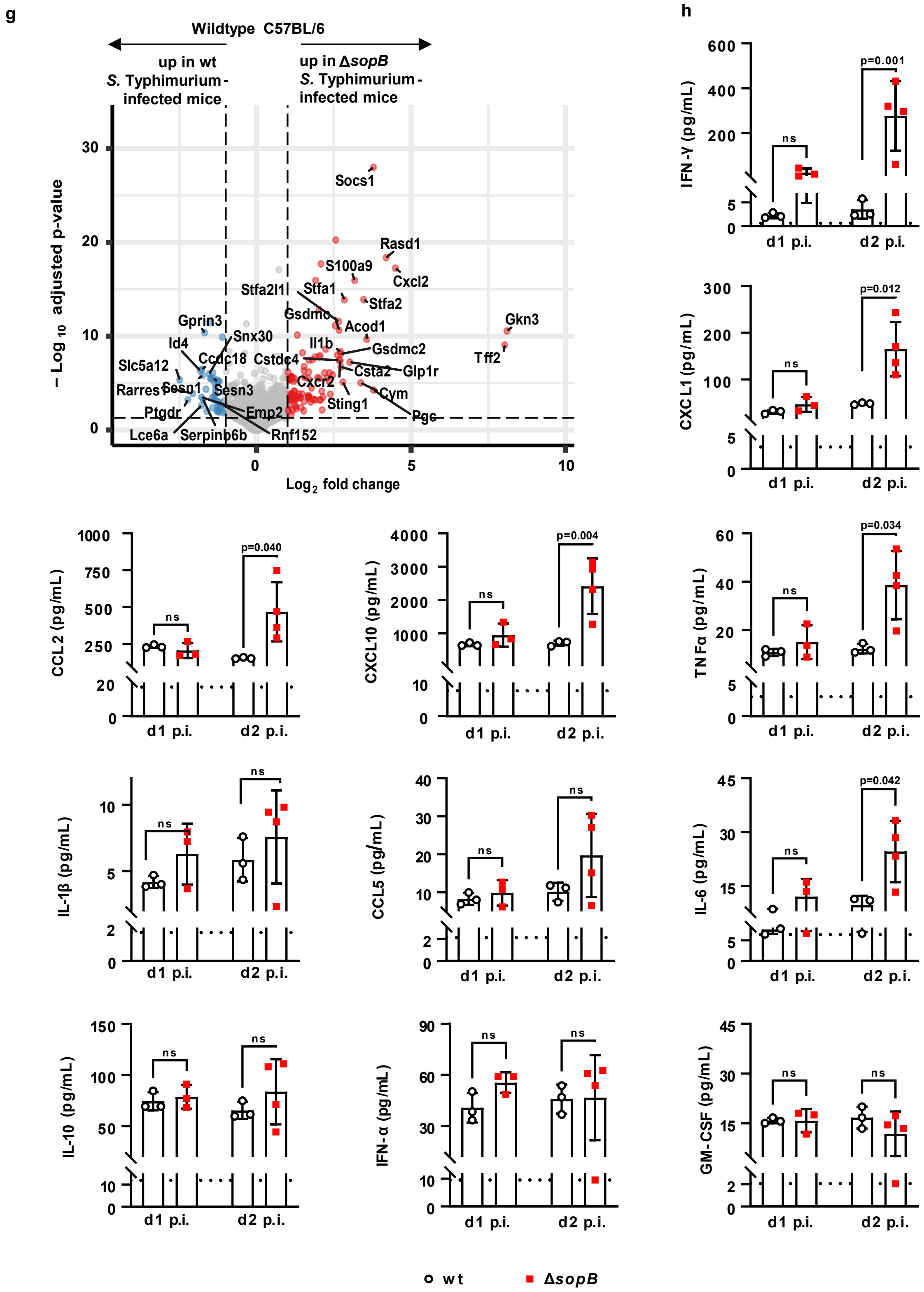

g
Wildtype C57BL/6
up in wt
S. Typhimurium-
infected mice
up in ΔsopB
S. Typhimurium-
infected mice
– Log 10 adjusted p-value
Log2 fold change
Socs1
Rasd1
S100a9
Cxcl2
Stfa2l1
Stfa1
Stfa2
Gsdmc
Acod1
Gkn3
Gprin3
Snx30
Id4
Il1b
Gsdmc2
Tff2
Ccdc18
Cstdc4
Slc5a12
Csta2
Glp1r
Cxcr2
Rarres1
Sesn1
Sesn3
Cym
Sting1
Ptgdr
Emp2
Pgc
Lce6a
Serpinb6b
Rnf152
h
IFN-γ (pg/mL)
p=0.001
CXCL1 (pg/mL)
p=0.012
CCL2 (pg/mL)
p=0.040
CXCL10 (pg/mL)
p=0.004
TNFα (pg/mL)
p=0.034
IL-1β (pg/mL)
CCL5 (pg/mL)
IL-6 (pg/mL)
p=0.042
IL-10 (pg/mL)
IFN-α (pg/mL)
GM-CSF (pg/mL)
ns
d1 p.i.
d2 p.i.
wt
ΔsopB

Supplementary Figure Legends

Supplementary Figure 1. The influence of SopB on enterocyte invasion, organ load, and gene expression. (a) Schematic diagram of the neonatal small intestinal epithelial stem cell organoid co-culture. Stem cell organoid cells grown as monolayers were infected for 1 h with wt, Δ*sopB*, or Δ*sopB* p*sopBsigE* (compl.) *S.* Typhimruium followed by a 1h incubation in 100 µg/mL gentamicin at 37°C. The number of viable bacteria analysed by serial dilution and plating. **(b)** Invasion rate expressed as the number of detected intracellular bacteria relative to the number of administered bacteria x 100 (in %). One data point represents one infected well, mean ± SD, three independent experiments. **(c and d)** Total bacterial count in mesenteric lymph node (MLN), liver and spleen tissue at day 1 **(c)** and 2 **(d)** p.i. **(e and f)** *Reg3g* and *Cxcl5* mRNA expression in total isolated intestinal epithelial cells at day 1 **(e)** and 2 **(f)** p.i. 1-day-old mice were left untreated (n=3) or infected with wt (n=5-11), Δ*sopB* (n=4-14), Δ*sopB* p*sopBsigE* (compl., n=4-9), or Δ$sopE_2$ (n=7-8, only for *Cxcl5*) *S.* Typhimurium. Values normalised to *Hprt;* shown as fold expression over non-infected controls. One data point represents one animal from at least two independent experiments, median. **(g)** Volcano plot of genes significantly differentially expressed (p adjusted<0.05 and |log2FC|>1) by intestinal epithelial cells isolated from wt (n=4) *versus* Δ*sopB S.* Typhimurium-infected wildtype neonates (n=4) at d1 p.i.. Genes with increased expression in Δ*sopB S.* Typhimurium-infected mice are shown in red; genes with increased expression in wt *S.* Typhimurium-infected mice in blue. **(h)** Data from the color-scaled heat map (z-score) shown in Fig. 1f. Serum concentration of the indicated cytokines and chemokines in pg/mL in the serum of mice infected with wt (n=3, black circles) or Δ*sopB* (n=3-4, red circles) *S.* Typhimurium at day 1 and 2 p.i. Mean ± SD. One data point represents one animal. The dashed line indicates the detection limit of the assay. One-way ANOVA **(b)**, Kruskal-Wallis combined with Dunn's multiple comparison test (**c-f**); two-way ANOVA with Sidak's multiple comparison test (**h**). ns, non-significant.

a

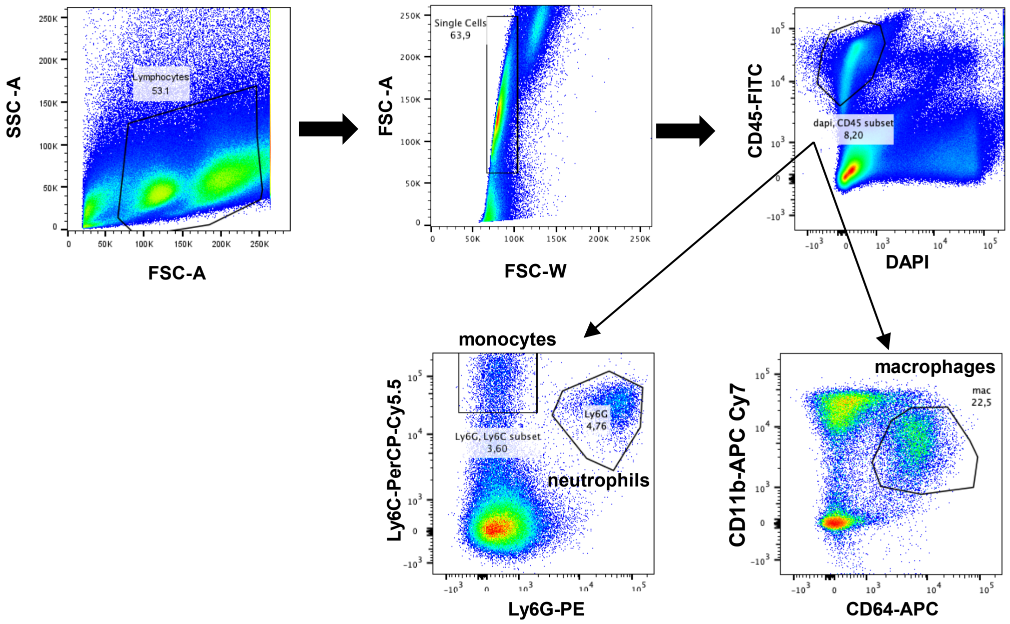

SSC-A
FSC-A
Lymphocytes
53,1
FSC-A
FSC-W
Single Cells
63,9
CD45-FITC
DAPI
dapi, CD45 subset
8,20
monocytes
Ly6C-PerCP-Cy5.5
Ly6G-PE
Ly6G, Ly6C subset
3,60
Ly6G
4,76
neutrophils
macrophages
mac
22,5
CD11b-APC Cy7
CD64-APC

Supplementary Figure 2. Flow cytometry gating strategy for immune cell quantification. **(a)** Gating strategy to identify *lamina propria* $Ly6C^{hi}Ly6G^{-}CD11b^{+}$ $MHCII^{lo/-}CD45^{+}DAPI^{-}$ monocytes, $Ly6G^{+}Ly6C^{int}CD11b^{+}$ $MHCII^{lo/-}CD45^{+}DAPI^{-}$ neutrophils and $CD64^{+}MHCII^{+}CD45^{+}DAPI^{-}$ macrophages by flow cytometric analysis (see Fig. 2).

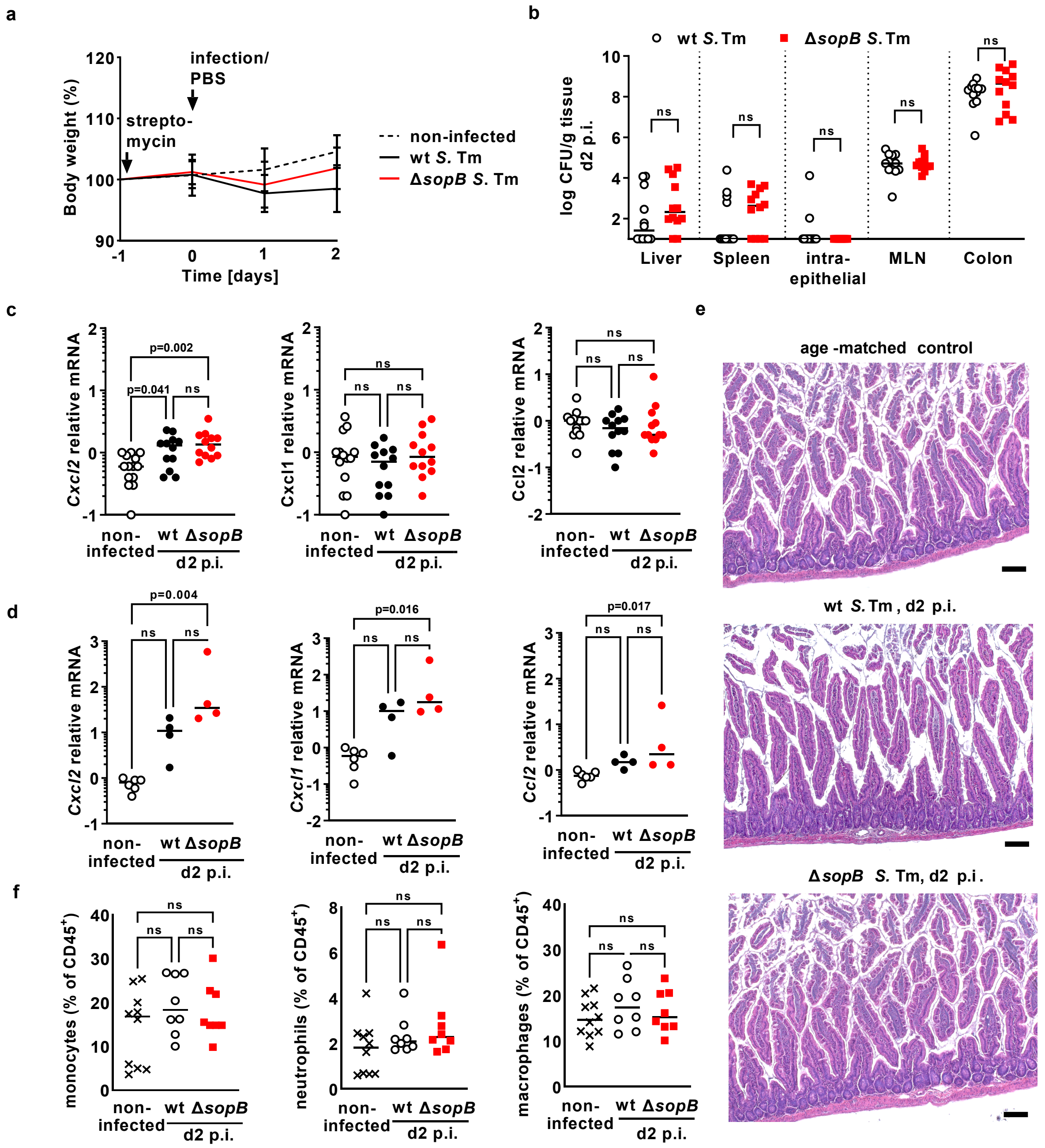
a
Body weight (%)
120
110
100
90
strepto-
mycin
infection/
PBS
-1
0
1
2
Time [days]
non-infected
wt S. Tm
ΔsopB S. Tm
b
wt S.Tm
ΔsopB S.Tm
log CFU/g tissue
d2 p.i.
10
8
6
4
2
ns
Liver
Spleen
intra-
epithelial
MLN
Colon
c
Cxcl2 relative mRNA
Cxcl1 relative mRNA
Ccl2 relative mRNA
p=0.002
p=0.041
ns
non-
infected
wt
ΔsopB
d2 p.i.
d
p=0.004
p=0.016
p=0.017
Cxcl2 relative mRNA
Cxcl1 relative mRNA
Ccl2 relative mRNA
e
age -matched control
wt S.Tm , d2 p.i.
ΔsopB S. Tm, d2 p.i.
f
monocytes (% of CD45+)
neutrophils (% of CD45+)
macrophages (% of CD45+)
non-
infected
wt
ΔsopB
d2 p.i.

Supplementary Figure 3. The role of SopB in the adult infection model. 9-week-old adult female mice were pretreated with streptomycin (20 mg) and 1 day later orally infected with wt (n=12) or Δ*sopB S*. Typhimurium (n=12) in PBS. Age-matched adult female mice treated with streptomycin and PBS were used as control (Co., n=14). **(a)** Total body weight was monitored daily from one day prior to infection until 2 days p.i. **(b)** The bacterial load in liver, spleen, mesenteric lymph nodes (MLN) and colon and the intraepithelial CFU determined by dilution and plating. **(c-d)** *Cxcl2, Cxcl1 and Ccl2* mRNA expression in **(c)** total isolated intestinal epithelial cells and **(d)** colon tissue at day 2 p.i. (wt, n=4-12, Δ*sopB*, n=4-12**,** co., n=6-14**).** Values normalised to *Hprt* and are shown as fold expression over non-infected controls. **(e)** Representative image of small intestinal tissue sections stained with H&E of non-infected age-matched control animals or adult mice infected with wt or Δ*sopB S*. Typhimurium at day 2 p.i. Bar=100 µm. **(f)** Flow cytometric analysis of *lamina propria* Ly6C^{hi}Ly6G^{-}CD11b^{+}MHCII$^{lo/-}$CD45^{+}DAPI^{-} monocytes, Ly6G^{+}Ly6C^{int}CD11b^{+}MHCII$^{lo/-}$CD45^{+}DAPI^{-} neutrophils and CD64^{+}MHCII^{+}CD45^{+}DAPI^{-} macrophages isolated at 2 days p.i. from wt (n=8) or Δ*sopB S*. Typhimurium (n=8) infected adult mice or healthy age-matched control animals (Co., n=10). One data point represents one animal. 3 independent experiments, median. Kruskal-Wallis with Dunn's posttest (**b-f**). ns, non-significant.

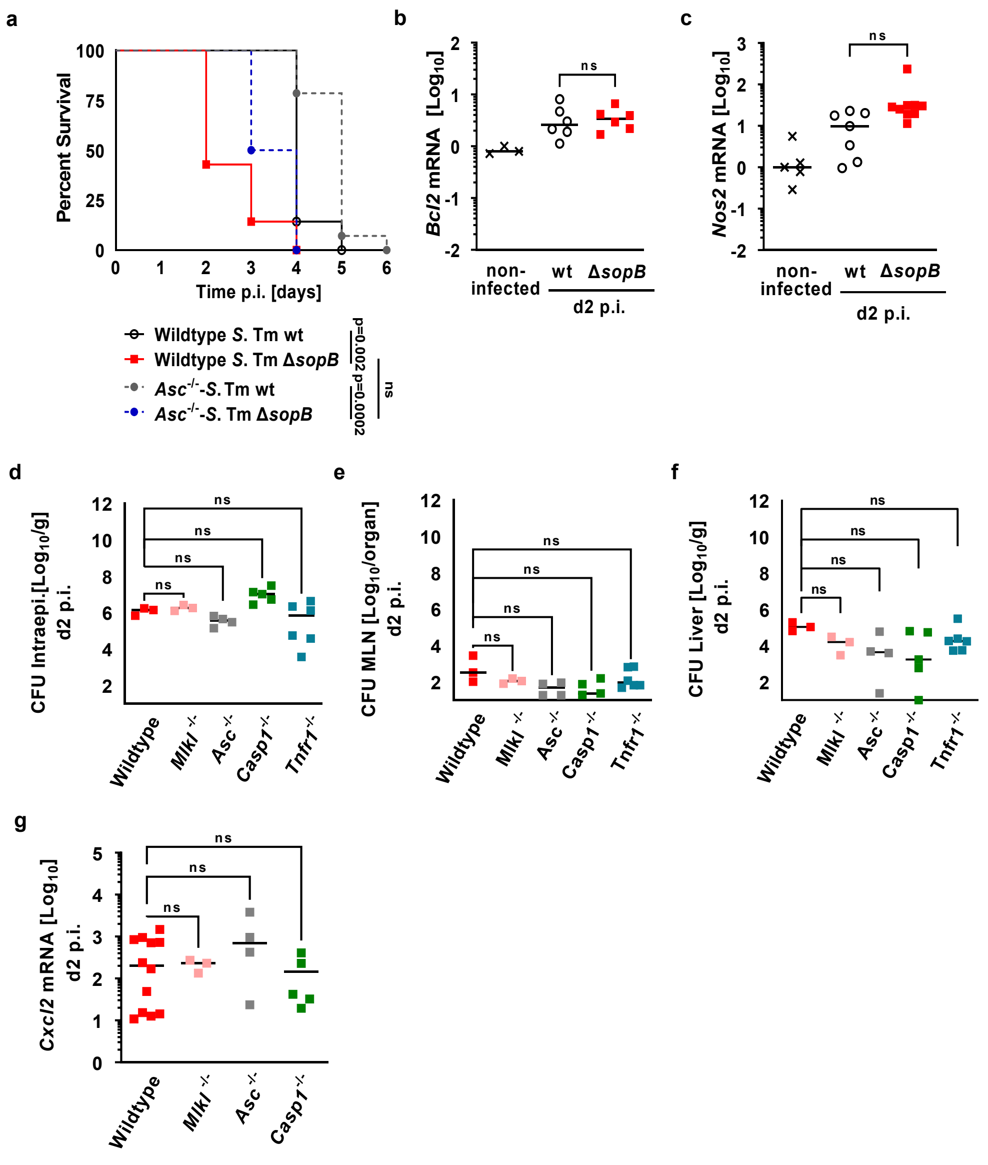
a
100
75
50
25
0
Percent Survival
0 1 2 3 4 5 6
Time p.i. [days]
Wildtype S. Tm wt
Wildtype S. Tm ΔsopB
Asc-/--S.Tm wt
Asc-/--S. Tm ΔsopB
p=0.002
p=0.0002
ns
b
Bcl2 mRNA [Log10]
ns
non-infected
wt
ΔsopB
d2 p.i.
c
Nos2 mRNA [Log10]
ns
non-infected
wt
ΔsopB
d2 p.i.
d
CFU Intraepi.[Log10/g] d2 p.i.
ns
Wildtype
Mlkl -/-
Asc -/-
Casp1 -/-
Tnfr1 -/-
e
CFU MLN [Log10/organ] d2 p.i.
ns
f
CFU Liver [Log10/g] d2 p.i.
ns
g
Cxcl2 mRNA [Log10] d2 p.i.
ns

Supplementary Figure 4. **Mechanisms of disease progression**. **(a)** Kaplan-Meier curve of 1-day-old wildtype (B6JWT, n=7 for both groups) and *Asc*$^{-/-}$ (n=6-14) neonates infected with wt or Δ*sopB S.* Typhimurium. Note that the groups of wt and Δ*sopB S.* Typhimurium-infected wildtype mice are identical to Figure 1a. **(b and c)** Quantitative RT-PCR analysis for *Bcl2* **(b)** and *Nos2* **(c)** mRNA expression in total intestinal epithelial cells isolated at day 2 p.i. from wildtype neonates infected at day 1 after birth with wt (n=6-7) or Δ*sopB* (n=6-9) *S.* Typhimurium or non-infected controls (n=3-5). Median. One data point represents one animal. **(d-f)** CFU of *sopB*-deficient (Δ*sopB*) *S.* Typhimurium in total isolated gentamicin-treated intestinal epithelial cells **(d)**, mesenteric lymph node (MLN) **(e)**, and liver tissue homogenates **(f)** of wildtype (n=3), *Mlkl*$^{-/-}$ (n=3), *Asc*$^{-/-}$ (n=4), *Casp1*$^{-/-}$ (n=5), and *Tnfrsf1a*$^{-/-}$ (n=6) mice at day 2 p.i. Median. One data point represents one animal. **(g)** Quantitative RT-PCR analysis for *Cxcl2* mRNA expression in total intestinal epithelial cells isolated at day 2 p.i. from wildtype (n=12), *Mlkl*$^{-/-}$ (n=3), *Asc*$^{-/-}$ (n=4), and *Casp1*$^{-/-}$ (n=5) neonates infected at day 1 after birth with Δ*sopB S.* Typhimurium. Kruskal Wallis test with Dunn's posttest (**b-g**). ns, non-significant.

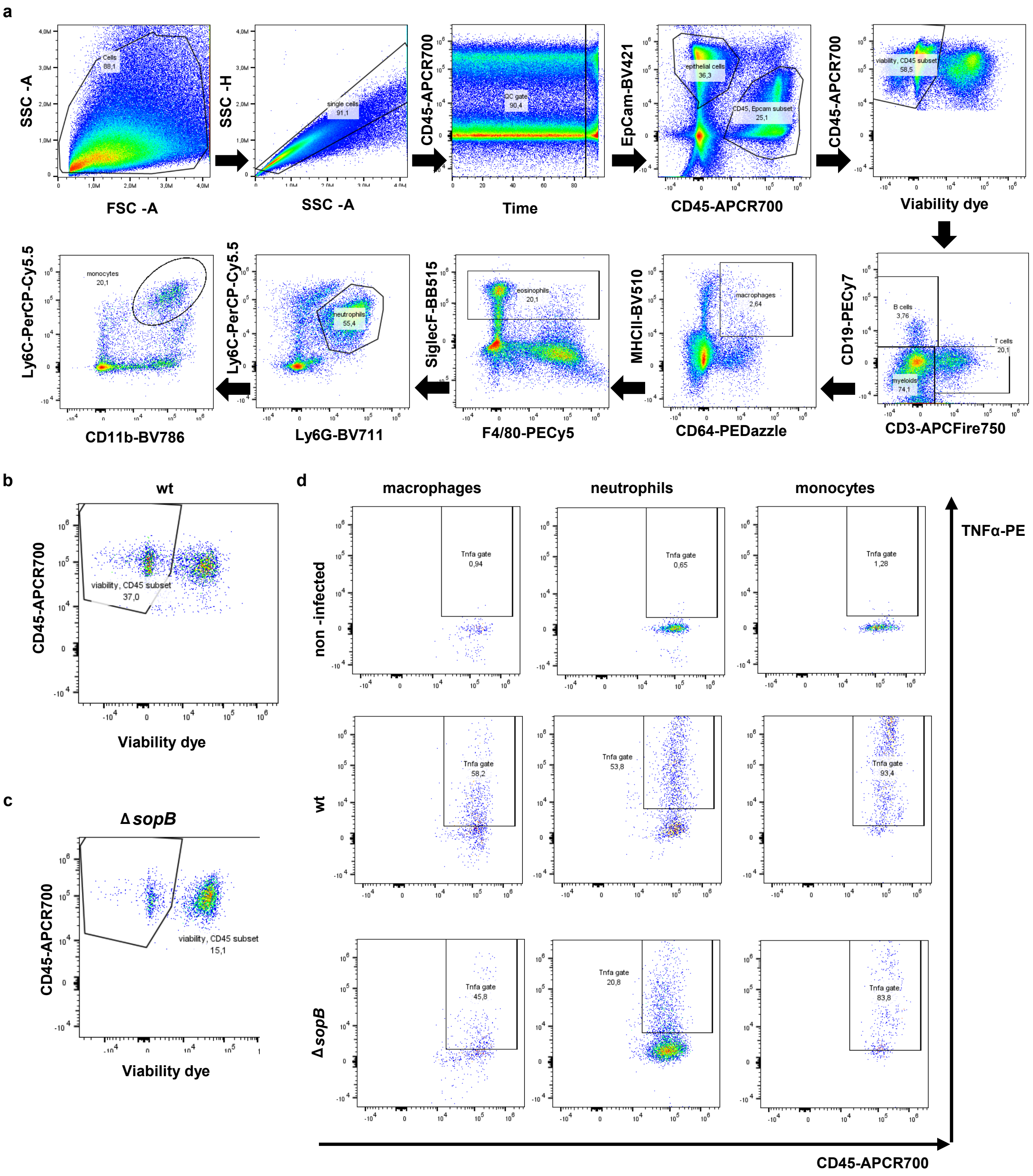
a
SSC -A
FSC -A
Cells
88,1
SSC -H
SSC -A
single cells
91,1
CD45-APCR700
Time
QC gate
90,4
EpCam-BV421
CD45-APCR700
epithelial cells
36,3
CD45, Epcam subset
25,1
CD45-APCR700
Viability dye
viability, CD45 subset
58,5
CD19-PECy7
CD3-APCFire750
B cells
3,76
T cells
20,1
myeloids
74,1
MHCII-BV510
CD64-PEDazzle
macrophages
2,64
SiglecF-BB515
F4/80-PECy5
eosinophils
20,1
Ly6C-PerCP-Cy5.5
Ly6G-BV711
neutrophils
55,4
Ly6C-PerCP-Cy5.5
CD11b-BV786
monocytes
20,1
b
wt
CD45-APCR700
Viability dye
viability, CD45 subset
37,0
c
Δ sopB
CD45-APCR700
Viability dye
viability, CD45 subset
15,1
d
macrophages
neutrophils
monocytes
non -infected
wt
Δ sopB
Tnfa gate
0,94
0,65
1,28
58,2
53,8
93,4
45,8
20,8
83,8
TNFα-PE
CD45-APCR700

Supplementary Figure 5: Flow cytometric gating strategy for the intracellular cytokine staining. **(a)** Gating strategy to identify *lamina propria* Ly6C^{hi}Ly6G^{-}CD11b^{+} MHCII$^{lo/-}$CD45^{+}DAPI^{-} monocytes, Ly6G^{+}Ly6C^{int}CD11b^{+} MHCII$^{lo/-}$CD45^{+}DAPI^{-} neutrophils and CD64^{+}MHCII^{+}CD45^{+}DAPI^{-} macrophages by flow cytometric analysis. **(b and c)** Flow cytometric analysis of the viability of non-stimulated or PMA/ionomycin stimulated monocytes isolated from the *lamina propria* of wt (n=4) **(b)** or Δ*sopB* (n=4) **(c)** *S.* Typhimurium-infected neonates at day 2 p.i. For this analysis, the upstream gating step on viable cells (see Fig. S4a) was omitted. **(d)** Flow cytometric staining of intracellular TNFα in Ly6C^{hi}Ly6G^{-}CD11b^{+} MHCII$^{lo/-}$CD45^{+}DAPI^{-} monocytes, Ly6G^{+}Ly6C^{int}CD11b^{+} MHCII$^{lo/-}$CD45^{+}DAPI^{-} neutrophils, and CD64^{+}MHCII^{+}CD45^{+}DAPI^{-} macrophages isolated from wt (n=4) or Δ*sopB* (n=4) *S.* Typhimurium-infected mice at day 2 p.i. after re-stimulation with PMA/ionomycin.

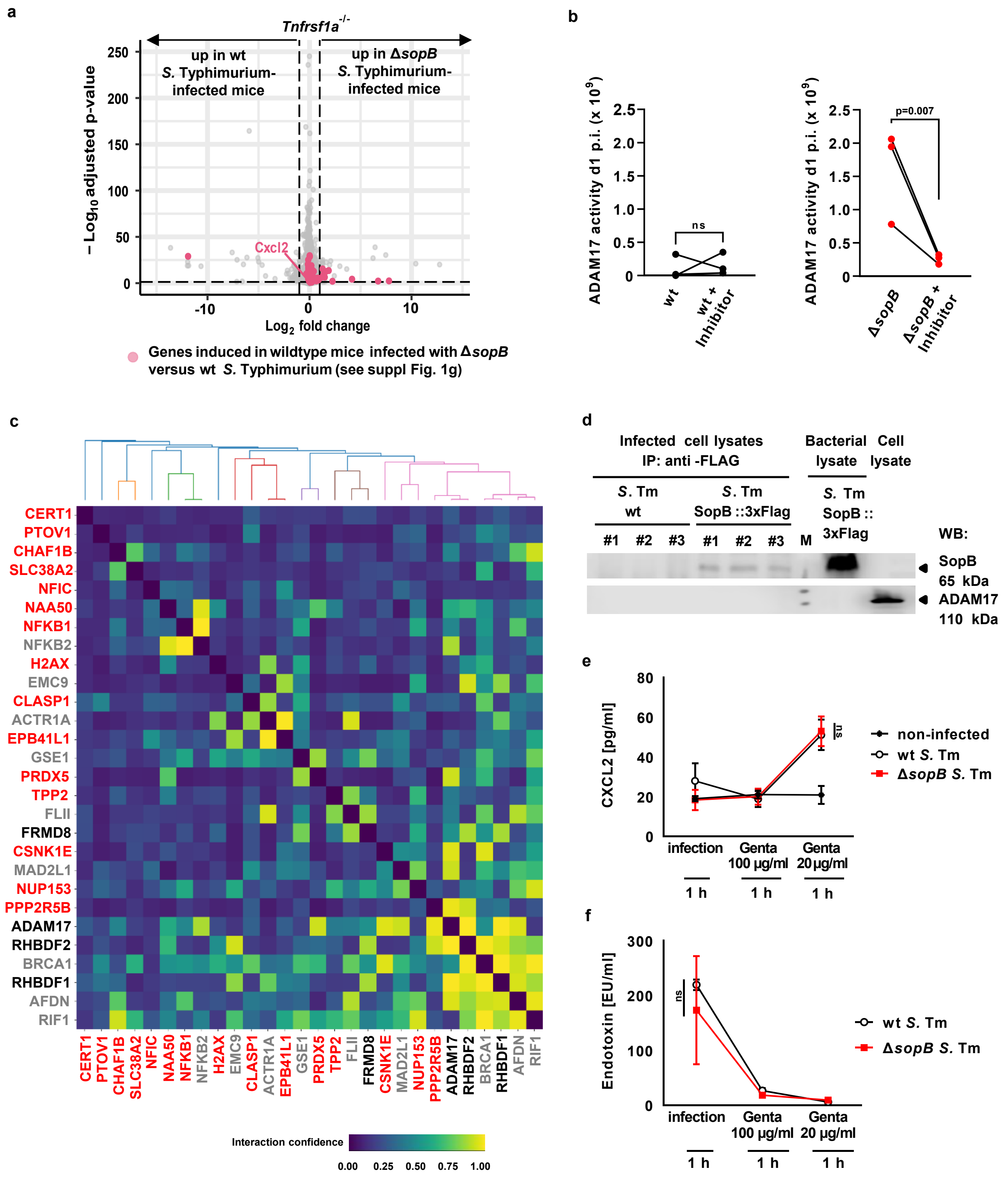
a
Tnfrsf1a-/-
up in wt
S. Typhimurium-
infected mice
up in ΔsopB
S. Typhimurium-
infected mice
Cxcl2
– Log10 adjusted p-value
Log2 fold change
250
200
150
100
50
0
-10
0
10
Genes induced in wildtype mice infected with ΔsopB
versus wt S. Typhimurium (see suppl Fig. 1g)
b
ADAM17 activity d1 p.i. (x 10^9)
2.5
2.0
1.5
1.0
0.5
0
ns
wt
wt + Inhibitor
p=0.007
ΔsopB
ΔsopB + Inhibitor
c
CERT1
PTOV1
CHAF1B
SLC38A2
NFIC
NAA50
NFKB1
NFKB2
H2AX
EMC9
CLASP1
ACTR1A
EPB41L1
GSE1
PRDX5
TPP2
FLII
FRMD8
CSNK1E
MAD2L1
NUP153
PPP2R5B
ADAM17
RHBDF2
BRCA1
RHBDF1
AFDN
RIF1
Interaction confidence
0.00
0.25
0.50
0.75
1.00
d
Infected cell lysates
IP: anti -FLAG
Bacterial lysate
Cell lysate
S. Tm wt
S. Tm SopB ::3xFlag
S. Tm SopB :: 3xFlag
#1
#2
#3
#1
#2
#3
M
WB:
SopB 65 kDa
ADAM17 110 kDa
e
CXCL2 [pg/ml]
80
60
40
20
0
ns
infection
Genta 100 µg/ml
Genta 20 µg/ml
1 h
1 h
1 h
non-infected
wt S. Tm
ΔsopB S. Tm
f
Endotoxin [EU/ml]
300
200
100
0
ns
infection
Genta 100 µg/ml
Genta 20 µg/ml
1 h
1 h
1 h
wt S. Tm
ΔsopB S. Tm

Supplementary Figure 6: Analysis of the interaction of SopB with host cell processes. (a) Volcano plot of genes significantly differentially expressed (p adjusted<0.05 and |log2FC|>1) by intestinal epithelial cells isolated from wt (n=4) *versus* Δ*sopB S.* Typhimurium-infected *Tnfrsf1a*$^{-/-}$ neonates (n=4) at d1 p.i.. Genes with increased expression wildtype mice infected with Δ*sopB S.* Typhimurium (see Suppl. Fig. 1g) are shown in pink. **(b)** ADAM17 activity in intestinal epithelial cells isolated from mice infected with wt (n=3, left panel) or Δ*sopB S.* Typhimurium (n=3, right panel) and incubated with ADAM17 substrate for 30 min. in the presence (+ inhibitor) or absence of an ADAM17-specific inhibitor. For the full kinetic see Fig. 5h. One representative data set of three independent experiments. **(c)** Heatmap of AlphaFold-Multimer–derived interaction confidence for all pairwise combinations analysed between SopB interactome candidates (red, see Fig. 6c), ADAM17 complex proteins (black), and intermediate interacting molecules (grey, see Fig. 6e) deduced by generating an integrated network connecting SopB interactome and ADAM17 complex protein using AFM-augmented PPIs (interaction confidence > 0.75). **(d)** Western blot of material obtained by immunoprecipitation with an anti-FLAG antibody from wt and *sopB*::3xFLAG *S.* Typhimurium infected m-IC$_{cl2}$ cells, as well as the bacterial pellet of *S.* Typhimurium *sopB*::3xFLAG (bacterial lysate) and total cell lysate of infected m-IC$_{cl2}$ cells (cell lysate) using an anti-FLAG antibody (SopB) or an anti-ADAM17 antibody (ADAM17). M, prestained protein ladder. **(e)** CXCL2 secretion into the cell culture supernatant by m-IC$_{cl2}$ cells left untreated (non-infected) or infected with wt or Δ*sopB S.* Typhimurium. One data point represents one technical replicate from at least two independent experiments, median ± SD. **(f)** Endotoxin in the basolateral compartment of m-IC$_{cl2}$ cells grown on Transwell inserts and infected with wt or Δ*sopB S.* Typhimurium and the endotoxin (LPS) concentration was determined at 1, 2, and 3 h of co-culture using the Kinetic-QCL™ Kinetic Chromogenic LAL Assay (Lonza). Median ± SD. Paired t-test (**a**) and Kruskal-Wallis test with Dunn's posttest (**d and e**). ns, non-significant.

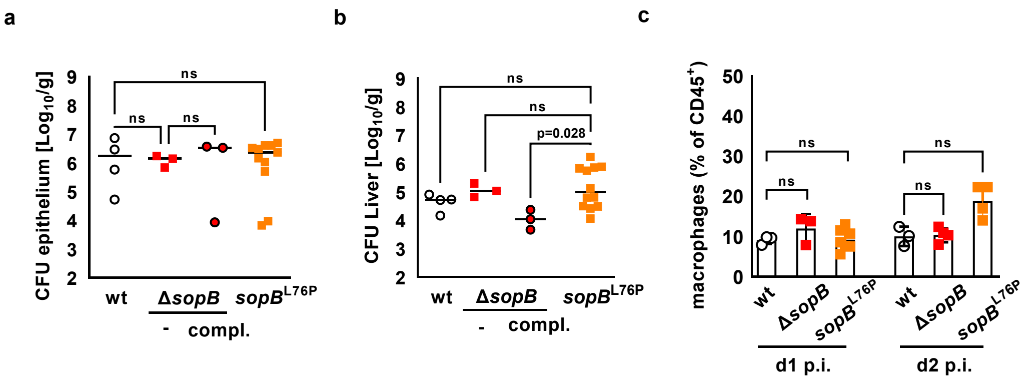
a
CFU epithelium [Log10/g]
9
8
7
6
5
4
3
2
ns
ns
ns
wt
ΔsopB
sopBL76P
-
compl.
b
CFU Liver [Log10/g]
ns
ns
p=0.028
wt
ΔsopB
sopBL76P
-
compl.
c
macrophages (% of CD45+)
50
40
30
20
10
0
ns
ns
ns
ns
wt
ΔsopB
sopBL76P
d1 p.i.
d2 p.i.

Supplementary Figure 7: Functional influence of the C- and N-terminal domain on the immune-suppressive phenotype of SopB. (a and b) Total bacterial count in isolated total epithelial cells **(a)** and liver tissue **(b)** at day 2 p.i. with 100 CFU wt (n=4), *sopB* deficient (Δ*sopB*, n=3), *sopB* deficient in trans *sopB* sigE complemented (Δ*sopB* p*sopBsigE*, n=3) or a *sopB*^{L76P} allele carrying *S.* Typhimurium (n=11-12). (**c**) Flow cytometric analysis of *lamina propria* $CD64^{+}MHCII^{+}CD45^{+}DAPI^{-}$ macrophages in 1-day-old C57BL/6 wild-type neonates orally infected with 100 CFU wt (n=3 at day 1 and 2 p.i.), Δ*sopB* (n=3 and 4 at day 1 and 2 p.i., respectively) or *sopB*^{L76P}-expressing *S.* Typhimurium (*sopB*^{L76P}, n=7 and n=4 at day 1 and 2 p.i., respectively) at day 1 and 2 p.i. Kruskal-Wallis combined with Dunn's multiple comparison test (**a and b**) and two-way ANOVA with Tukey's multiple comparison test (**c**). ns, non-significant.

Supplementary Tables

Supplementary Table 1: Bacterial strains used in this study

strain name	genotype	application	source/reference
ATCC14028	wt	parental to mutant strains	ATCC
MvP1208	sopB::aph	deletion mutant strain KanR	Zhang et al., 2018
MvP1211	sopB::FRT	deletion mutant strain, markerless	this study
MvP2726	ΔsopB::I-SceI	parental to mutant strains	this study
MvP2736	sopB::HA	allelic exchange in MvP2726	this study
MvP2734	sopB::HA C460S	allelic exchange in MvP2726	this study
MvP2735	sopB::HA K528A	allelic exchange in MvP2726	this study
MvP3325	sopB::HA L76P	allelic exchange in MvP2726	this study
MD1163	sopB::3xFlag aph	chromosomal tag in SL1344	Cortes-Avalos et al., 2024
MvP3036	sopB::3xFlag aph	P22 transduction from MD1163	this study

Supplementary Table 2: Oligonucleotides used in this study

**λ Red mutagenesis**	
Designation	Sequence (5’ - 3’)
sopB In717 For	CGTCGGGTTACTCACCGCGTCGAATATTTTCGGCAAAGAGAGGGT TTTCCCAGTCACGAC
sopB In717 Rev2	ACGATTTAATAGACTTTCCATATAGTTACCTCAAGACTCATGCTTC CGGCTCGTATGTTG
sopB TC2 For	CGTCACGGTCTTACTTGTCC
sopB TC2 Rev	TAGACTTTCCATATAGTTACCTCAAGACTCAAGATGTGATCATAG CGTTTTTAATATTCC
sopB-HA RedIn Rev	ACGATTTAATAGACTTTCCATATAGTTACCTCAAGACTCAAGCGT AGTCTGGGACGTCGT
**Control PCR**	
Designation	Sequence (5’ - 3’)
sopB DelCheck For	TACGTATGGACGTCAGGATG
sopB DelCheck Rev	CAATGGCATAAAGGGACAGC
sigE DelCheck Rev	CCATAACGGTGAAGCAGAAG
k1 RedDel	CAGTCATAGCCGAATAGCCT
Seq For	CGCCAGGGTTTTCCCAGTCACGAC
M13 (-29)	CAGGAAACAGCTATGACC
Z1652 Check For	GTAATGGGAAAGGACGCTCATTGC
**Cloning (*E*. coli and *S*. Typhimurium)**	
Vf pWSK-Pn	CAGCTTTTGTTCCCTTTAGTGA
Vr pWSK29	GTGAGTCGTATTACGCGCGCTC
Vr p4042	TTAAGCGTAGTCTGGGACG
1f pWSK29-PsopB	GCGCGTAATACGACTCACTCACGGTCTTACTTGTCC
1f HA-sig	CGTCCCAGACTACGCTTAATTGAGTCTTGAGGTAACTATATG
1r sigE-pWSK	TCACTAAAGGGAACAAAAGCTGTAAAGGGCATACGTATCG
**Site-directed mutagenesis***	
sopB L76P For	GCTGCATAACccgTATAACTTACAGC
sopB L76P Rev	GAGTGGTTAGACGCC
sopB C460S For	CGCCTGGAATTCTAAAAGCGGCA
sopB C460S Rev	GGTACCGCGTCAATTTCATG
sopB K528A For	CAAAGTAATGGCAAATTTATCGCCAGAGGTGCTCAATC
sopB K528A Rev	TTTCCCGCCCCGCCCGTA

*Lowercase, mutated nucleotide positions

Supplementary Table 3: Plasmids used in this study

plasmids	genotype	application	source/reference
pWSK29	low copy number vector, AmpR	cloning	lab stock
pWRG730	Red expression, I-SceI, CmR	mutagenesis	Hoffmann et al., 2017
pWRG717	template for aph I-SceI site	template for mutagenesis	Hoffmann et al., 2017
p4042	pWSK29 sopB::HA	Complementation, generation of MvP2736	Zhang et al., 2018
p5132	pWSK29 sopB::HA sigE	complementation	this study
p5133	pWSK29 sopB sigE	complementation	this study
p4986	pWSK29 sopB::HA C460S	generation of MvP2734	this study
p4988	pWSK29 sopB::HA K528A	generation of MvP2735	this study
p6768	pWSK29 sopB::HA L76P	generation of MvP3325	this study
