# Supplementary material for: Salmonella SopB suppresses post-transcriptionally regulated cytokine release to reduce early tissue inflammation and delay disease progression
Source: Nat Commun. 2026 Jul 6;17:5884. doi: 10.1038/s41467-026-74942-9 (PMC13338139; doi:10.1038/s41467-026-74942-9)
Supplement: Supplementary file 2 — Reporting Summary [file 41467_2026_74942_MOESM2_ESM.pdf]

nature portfolio

Corresponding author(s): Mathias Hornef and Kaiyi Zhang

Last updated by author(s): May 31, 2026

Reporting Summary

Nature Portfolio wishes to improve the reproducibility of the work that we publish. This form provides structure for consistency and transparency in reporting. For further information on Nature Portfolio policies, see our Editorial Policies and the Editorial Policy Checklist.

Statistics

For all statistical analyses, confirm that the following items are present in the figure legend, table legend, main text, or Methods section.

n/a	Confirmed	
☐	☒	The exact sample size (*n*) for each experimental group/condition, given as a discrete number and unit of measurement
☐	☒	A statement on whether measurements were taken from distinct samples or whether the same sample was measured repeatedly
☐	☒	The statistical test(s) used AND whether they are one- or two-sided<br>*Only common tests should be described solely by name; describe more complex techniques in the Methods section.*
☐	☒	A description of all covariates tested
☐	☒	A description of any assumptions or corrections, such as tests of normality and adjustment for multiple comparisons
☐	☒	A full description of the statistical parameters including central tendency (e.g. means) or other basic estimates (e.g. regression coefficient) AND variation (e.g. standard deviation) or associated estimates of uncertainty (e.g. confidence intervals)
☐	☒	For null hypothesis testing, the test statistic (e.g. *F*, *t*, *r*) with confidence intervals, effect sizes, degrees of freedom and *P* value noted<br>*Give P values as exact values whenever suitable.*
☒	☐	For Bayesian analysis, information on the choice of priors and Markov chain Monte Carlo settings
☒	☐	For hierarchical and complex designs, identification of the appropriate level for tests and full reporting of outcomes
☒	☐	Estimates of effect sizes (e.g. Cohen's *d*, Pearson's *r*), indicating how they were calculated

Our web collection on statistics for biologists contains articles on many of the points above.

Software and code

Policy information about availability of computer code

Data collection	Bacterial viable counts in isolated cells, total homogenized tissue or infected stem cell organoids were obtained by serial dilution and plating of homogenised tissue on LB agar plates supplemented with the appropriate antibiotic(s).<br>RT-PCR was performed using Taqman technology with an absolute QPCR ROX mix (Thermo Scientific).<br>RNS seq data: Libraries were prepared with the QuantSeq 3'mRNA-Seq v2 Library Prep Kit FWD with UDIs (Lexogen), using an input of 125 ng, and sequenced in single end mode (read 1: 75 cycles, index 1: 12 cycles, index 2: 12 cycles, read 2: 0 cycles) on a NovaSeq 6000 (Illumina), using a NovaSeq 6000 SP Reagent Kit v1.5 (100 cycles) (Illumina).<br>For cytokine quantification, including the concentrations of TNF and IL-18 in the medium supernatants and serum samples were measured using the LEGENDplexTM Mouse Virus Response Panel (BioLegend, Cat No 740622) and LEGENDplex™ Mouse M1 Macrophage Panel (BioLegend, Cat. Nr 740848) according to the manufacturer’s protocol. Samples were measured using a BD FACS Canto II. CXCL2 secretion by m-ICcl2 cells was quantified using a CXCL2 ELISA from Biosite (Cat. No.: PPE 21335).<br>The endotoxin concentration in cell culture medium was measured using the Kinetic-QCLTM Kinetic chromogenic LAL assay (Lonza, Cat. No.: 50-650). Immune cells were separated using a Percoll gradient by centrifugation at 700 x g for 20 min. at room temperature. Cells were stained using the following antibodies CCD45-FITC (Clone 30-F11), Ly6C-PerCPCy5.5 (Clone HK1.4), Ly6G-PE (Clone 1A8), Ly6G-Spark NIR 685 (Clone 1A8), Ly6C-BV711 (HK1.4), CD11b-APC Cy7 (Clone N418), CD11b-BUV 395 (Clone M1/70), CD64-APC (Clone X54-5/7.1), CD64-PE Dazzle (Clone X54-5/7.1), MHCII-AF488 (Clone M5/114.15.2), MHCII-BV510 (Clone M5/114.15.2), PDL1-PE (Clone 10F.952), SiglecF-APCR700 (Clone 90/CD38; BD), Epcam-BV421 (Clone G8.8), CD3-FITC (Clone 17A2), CD19-FITC (Clone 6D5), (Biolegend) and DAPI (Roth) for subsequent analytical flow cytometry. Data were acquired with a BD FACS Canto II.<br>Intracellular cytokine staining: Cells were then harvested and stained with the following antibodies (Biolegend): CD45-APCR700 (Clone 30-F11; BD Biosciences), CD3-APCFire750 (Clone 17A2), PDL1-APC (Clone 10F.952), SiglecF-BB515 (Clone E50-2440; BD Biosciences), CD11c-BUV737 (Clone N418: BD Biosciences), CD64-PEDazzle (Clone 90/CD38), CD11b-BV786 (Clone M1/70), F480-PECy5 (Clone BM8), Epcam-BV421 (Clone G8.8), MHCII-BV510 (Clone M5/114.15.2), Ly6C-PerCP-Cy5.5 (Clone HK1.4), Ly6G-BV711 (Clone 1A8), CD80-BUV 805 (Clone 16-10A1; BD

Biosciences), CD19-PECy7 (Clone 6D5) for 20 min at 4°C and 30 min with Zombie UV at 4°C (Cat. No.: 423107, BioLegend). Stained cells were fixed and permeabilised (BD Cytofix/Cytoperm™, Cat. No. 554722 according to manufacturer`s instructions) prior to intracellular cytokine staining with a TNFα-PE (Clone MP6-XT22) antibody overnight at 1:500 dilution (Biolegend). Data were acquired on a Cytek Aurora flow cytometer.

For immunostaining, images were taken using a Zeiss ApoTome.2 system microscope connected to an Axiocam 506 digital camera (Zeiss).

ADAM17 activity assay: Cell pellets were incubated at 37°C in a humidified 5% CO2 incubator for 30, 60, 90, 120 min and 180 min in the presence of 10 µM ADAM17/TACE substrate (Sigma, Cat. Nr: 616407), with or without 10 µM ADAM17/TACE inhibitor (Sigma, GW-3333), in a total volume of 50 µl PBS. ADAM17 enzymatic activity was quantified at the indicated time points by measuring fluorescence intensity at Ex/Em=320 nm/420 nm using a fluorescence microplate reader (SpectraMax i3, ROM v1.4 b18). At the end of the incubation period, IEC pellets were lysed using 0.1% Triton X-100 (Cayman, item: 601172) and total protein concentrations of the lysates were determined using the Bradford assay (Bio-Rad) following the manufacturer's instructions.

Phosphoproteomics: m-ICcl2 cells were grown to confluency and polarised for 7 days. Cells were infected at a MOI of 10:1 for 1 h, non-infected cells served as a control. Cells were lysed in 1% Triton X-100, 150 mM NaCl, 50 mM Tris-HCl (pH 7.4), 0.5% sodium deoxycholate, and 0.1% SDS including Roche's complete proteinase and phosphatase inhibitors. Experiments were conducted in 4 biological replicates. For the full proteome, 30 µg protein from each replicate was used and prepared by protein clean up and enzymatic cleavage using a paramagnetic bead approach as described previously 64. Briefly, the volume of protein samples was adjusted to 50 µL with 100 mM TEAB (Tetraethylammonium tetrahydroborate, Sigma-Aldrich, USA), followed by reduction with 5 µL 200 mM TCEP (Tris(2-carboxyethyl)phosphine hydrochloride, Sigma-Aldrich, USA) in 100 mM TEAB for 1 h at 55 °C. Subsequently, 5 µL 375 mM iodoacetamide (Merck KGaA, Germany) in 100 mM TEAB was added and incubated for 30 min at room temperature in the dark. 2 µL SP3 beads per sample were washed with water three times with subsequent addition of the sample. After protein binding to the beads, the supernatant was discarded. Then, the beads were washed twice with 200 µL 70 % (v/v) ethanol, and once with 200 pure ACN. Finally, the proteins were digested with trypsin (Promega, Germany) in a ratio of 1:50 for 16 h at 37 °C. Subsequently, a peptide clean-up was conducted. Therefore, ACN was added to each sample to reach a final organic content higher than 95 % (v/v). After peptide binding to the beads, the samples were washed with pure ACN on the magnetic rack. Peptides were eluted in two fractions, the first one with 87% acetonitrile in 10 mM ammonium formate (pH 10, Sigma Aldrich), and the second one with 2% dimethylsulfoxide (DMSO, Sigma Aldrich). Both fractions were analysed using liquid chromatography (LC) coupled to a mass spectrometer (MS). In detail, the peptides were separated on a nano-UPLC system (Ultimate 3000, Dionex, USA) with a trapping column (flow rate 5 µl/min, Acclaim PepMap 100 C18, 3 µm, nanoViper, 75 µm×5 cm, Thermo Fisher, Germany) and an analytical column (flow rate 0.3 µl/min, Acclaim PepMap 100 C18, 3 µm, nanoViper, 75 µm × 25 cm, Thermo Fisher, Germany) using a 160 min non-linear gradient as described in 64. The nano-UPLC system was coupled to the MS (QExactive HF, Thermo Scientific, USA) via a chip-based ESI source (Nanomate, Advion, USA). The only difference compared to the previously described workflow 64 was that not the top 10 but the top 15 precursor ions were subjected to MS/MS analysis. The obtained raw data were processed against the UniProtKB reference proteome of Mus musculus (March, 18, 2023), using Proteome Discoverer 2.5 and the following parameters: carbamidomethylation as fixed modifications, oxidation of methionine and acetylation of the protein N-terminus as variable modifications. This workflow resulted in information on 4682 proteins. For the phosphoproteome, 600 µg protein were used, followed by protein clean up and enzymatic cleavage using a paramagnetic bead approach as described above and previously 65. Peptides were eluted after the peptide clean-up in water resulting in one fraction. After elution, a two-step enrichment of phosphorylated peptides using the HighSelect™ TiO2 Phosphopeptide Enrichment Kit (Thermo Scientific, USA) and the High-Select™ Fe-NTA Phosphopeptide Enrichment Kit (Thermo Scientific, USA) was performed as described before 65. Enriched phosphorylated peptide samples were analysed using the same LC-MS/MS system as the full proteome samples with a 160 min non-linear gradient and with adjusted MS parameters: precursors between 350 m/z and 1550 m/z were detected at a resolution of 120K. MS1 automatic gain control (AGC) target was set to 3e6, with a maximum injection time of 150 ms. The top 15 precursors were isolated using a window of 0.7 Th, with MS2 AGC target 2e5 and a maximum injection time 150 ms. The normalised collision energy (NCE) was 34, fixed first mass 120 m/z, and MS2 resolution 60K. A dynamic exclusion of 45 s was used.

Affinity enrichment of SopB associated proteins: Polarized and confluent cell layers of m-ICcl2 cells were infected with S. Typhimurium chromosomally carrying a triple Flag-tagged sopB construct at a multiplicity of infection (MOI) of 10:1, or an untagged wildtype strain as a background control. Plates were centrifuged at 1,200 rpm for 5 min to initiate host cell contact. After 1 h incubation at 37 °C, cell monolayers were washed with cold PBS and lysed in Pierce RIPA buffer (Thermo Scientific) supplemented with cOmpleteTM protease inhibitor tablet (Roche) and PhosSTOP (Roche). The cell lysate was harvested by centrifugation at 13,000 rpm for 20 min at 4°C and mixed with 30 µL of washed anti-FLAG® M2 Affinity Gel (Sigma, A2220). The mixture was rotated at 4°C for 4 h to allow binding. Unbound proteins were washed away with 0.01% PBS-Triton X-100 buffer at 5,000 g for 5 min. at 4°C. The bound proteins were eluted using 150µg/mL FLAG peptide (Waters) prepared in 0.05% RapiGest (Waters). The eluted proteins were resuspended in 50 mM HEPES (pH 8) containing 1% SDS, 40 mM 2-chloroacetamide, and 10 mM TCEP, then incubated at 95°C for 5 min to facilitate reduction and alkylation. Nucleic acids were digested with Benzonase (2.8 U/Sample) at 37°C for 30 min. Samples were processed for mass spectrometry using a modified SP3 protocol (Hughes et al., 2019). Proteins were digested with Trypsin and LysC at 37°C for 14 h. Peptides were labeled using 6plex TMT (Thermo Fisher) following Zecha et al. (Creskey et al., 2023). A total of 6 samples (3 test samples and 3 background controls from three biologically independent experiments performed on separate days) were pooled and desalted using a Waters OASIS HLB µElution Plate. LC-MS/MS was performed on an UltiMate 3000 RSLCnano coupled to an Orbitrap Exploris 480 mass spectrometer (Thermo Fisher). Peptides were separated on a C18 analytical column (IonOpticks) over 160 min (1–40% B, 0.25 µl/min). MS1 spectra (400–1,600 m/z) were acquired at 120,000 resolution; the top N precursors (charge 2–5, cycle time 3s) were fragmented (NCE 32) and analyzed at 15,000 resolution.

Immunoprecipitation: Affinity purification of SopB-interacting proteins: For immunoblotting of ADAM17, protein eluted from the anti-FLAG® M2 Affinity Gel, total m-ICcl2 cell lysate, and S. Typhimurium sopB::3xFLAG lysate were incubated at 95°C for 10min with 4x SDS loading dye, loaded on a 10% SDS-PAGE, and run at 120V for 60min. Proteins were transferred to a nitrocellulose membrane at 250mA for 90min. The membrane was blocked with 5% milk-TBS-T for 1h at room temperature and incubated with anti-FLAG® M2 antibody diluted 1:2000 (F1804, Sigma) or anti-ADAM17 antibody diluted 1:2000 (JM10-35, Invitrogen) overnight at 4 °C. After washing three times with TBS-T, the membrane was incubated with the secondary antibody conjugated to HRP for 1h at room temperature in 5% milk-TBS-T. Finally, the washed membrane was incubated with SuperSignal West Femto Maximum Sensitivity Substrate (Thermo Scientific) and scanned using a C-DiGit Blot Scanner (LICORbio).

Data analysis

For RT-PCR, results were calculated by the 2−ΔΔCt method. Values were normalised to the Hprt housekeeping gene and are presented as fold induction over age-matched healthy controls.

For RNS Seq, raw sequencing reads were trimmed using Cutadapt v4.9 with the following parameters: -j 16 -a "poly A=A(20)" --quality-cutoff 20 -m 20 -u 12, to remove poly-A tails, low-quality bases, and adapter sequences. A genome index was generated using STAR v2.7.11b in genome generation mode (--sjdbOverhang 63) based on the GRCm39.112 mouse reference genome. Trimmed reads were then aligned to the genome index using STAR with parameters --outSAMtype BAM SortedByCoordinate and --quantMode GeneCounts to produce BAM files. Gene-level count matrices were generated using the featureCounts function from the Rsubread package (v2.14.2). Differential gene expression analysis was performed using DESeq2 v1.44.0, including only genes with a total count of at least 60 across all samples. GO

enrichment analysis was conducted using the clusterProfiler package (v4.12.6) with the org.Mm.eg.db database. Multiple testing correction was applied using the Benjamini-Hochberg method. GSEA was performed with the python gseapy package (v1.1.9) using genes from the mmu04668 KEGG pathway.

Cytokine and chemokine dresults were analysed with the LEGENDplex™ Data Analysis Software Suite (Qonit). Results are expressed as picograms per milliliter. The heat map was generated using Heatmapper (http://www.heatmapper.ca).

FACS data including the intracelluzlar cytokine stainings were analysed with FlowJo X.

The invasion rate was calculated as (number of intracellular bacteria/number of administered bacteria) X 100[%].

Phosphoproteomics: The obtained raw data were processed against the same UniProtKB reference proteome as the proteome, using Proteome Discoverer 2.5 and the following parameters: phosphorylation on serine, threonine, or tyrosine, oxidation of methionine, and acetylation of the protein N-terminus as variable modifications. This workflow resulted in information on 5242 proteins, 44225 peptide isoforms, and 15018 phosphosites. For the identification of regulated proteins/phosphosites and enrichment analysis, the data were first filtered for proteins and phosphosites identified at least in three replicates, followed by log2-transformation and median-normalisation. The average Log2(FCs) were calculated, and regulated proteins and phosphosites were determined using the student's t-test conducted in R studio 3.6.1. Obtained p-values were adjusted for multiple testing, according to Benjamini & Hochberg. Proteins and sites were considered significantly regulated with FDR≤0.05. Enrichment analyses were conducted with regulated proteins or regulated phosphosites (FDR≤0.05) using Ingenuity Pathway Analysis (IPA, Qiagen). Enrichment p-values were calculated with the right-tailored Fisher's exact test and adjusted for multiple testing, according to Benjamin & Hochberg. Pathways were considered significantly enriched with FDR≤0.05.

Affinity enrichment of SopB associated proteins: Raw files were converted to mzML (MSConvert v3.0.23129) format prior to database searching. Peptide and protein identification was performed using the TMT10 workflow implemented in MSFragger (v3.8) through FragPipe (v20.0). Spectra were searched against the UniProt Mus musculus reference proteome (UP000000589, downloaded April 8, 2025) and Salmonella enterica serovar Typhimurium strain 14028S proteome (UP000002695, downloaded April 8, 2025), supplemented with common contaminant proteins and reverse decoy sequences. Searches were performed using strict trypsin specificity, allowing up to two missed cleavages and a minimum peptide length of seven amino acids. Precursor and fragment mass tolerances were set to −20 to +20 ppm and 20 ppm, respectively. Carbamidomethylation of cysteine and TMT labeling of lysine residues were specified as fixed modifications. Oxidation of methionine (maximum three occurrences per peptide), protein N-terminal acetylation, and TMT labeling of peptide N-termini were included as variable modifications. Peptide-spectrum matches, peptides, and proteins were filtered at a 1% false discovery rate. Proteins identified by at least one unique peptide were retained for downstream analysis. Downstream analyses, including normalization (VSN) and differential expression, were conducted in R (RStudio v2021.09.2) using limma (v3.54.2), vsn (v3.66.0), and the tidyverse suite (dplyr v1.1.1, ggplot2 v3.4.2). Differential abundance was assessed using linear modeling with empirical Bayes moderation implemented in limma. Statistical significance was determined using two-sided moderated t-tests, and p-values were adjusted for multiple testing using the method of Benjamini–Hochberg.

Network analysis: To identify potential interactions between SopB affinity enriched host proteins and the ADAM17 complex consisting of ADAM17, iRhom1/RHBDF1, iRhom2/RHBDF2, and FRMD8, the curated interactomics database BioGRID was queried. To infer candidate–complex connectivity via direct interactions and shared interactors, we performed high-throughput AlphaFold-Multimer (AFM) prediction and interaction confidence scoring 68,69. Protein sequences were retrieved via the UniProt API 70 using custom Python scripts (Python v3.11.0). Proteins larger than 650 residues were segmented into prediction units using AlphaFold DB–derived pLDDT and PAE profiles to place boundaries in low-confidence regions between structural domains. All pairwise combinations were subjected to AFM prediction 71 using MSAs generated with MMseqs2 72. For each protein pair, AFM produced five models, which were ranked using the actifpTM score 73. A custom pipeline extracted Cα coordinates from both chains and defined interface residue pairs as positions within 10 Å. For each model, the mean interface PAE (iPAE) across all interface residues was computed and transformed to a normalised score by mapping iPAE ≤ 5 Å to 1, iPAE > 15 Å to 0, and linearly scaling intermediate values (5–15 Å). The model interaction score was calculated as the arithmetic mean of actifpTM and iPAE. Pairwise confidence was reported as the mean across the five AFM models (reflecting consensus across AFM weight sets), and high-confidence PPIs for network inclusion were defined as mean interaction confidence > 0.75. In addition, connectivity was augmented using a directed kinase-to-substrate reference database assembled by integrating curated resources: OmniPath 74, HPRD 75, PhosphoSitePlus 76, Phospho.ELM 77, Reactome 78 and DEPOP 79. Network construction and visualisation were performed with custom Python scripts; scripts are available upon reasonable request or, after publication, via https://github.com/Clusterbiology.

Statistics: Measurements were taken from distinct samples. Survival was analysed by log-rank (Mantel -Cox) test. The non-parametric Mann Whitney test was used for the comparative analysis of two groups. The Kruskal-Wallis combined with Dunn's multiple comparison test was employed for the statistical analysis of more than two groups. If data were normally distributed as confirmed using the Shapiro-Wilk test, the student's t-test (two groups) or the one-way ANOVA test with Tukey's posttest (more than two groups) was used. Two-way ANOVA with Sidak or Tukey's multiple comparison test was employed for the statistical analysis of two groups that have been split on two independent variables. Graphpad Prism Software 10 was used for statistical evaluation. Differences were considered significant at p <0.05, *; p <0.01, **; p <0.001, ***; and p <0.0001, ****.

For manuscripts utilizing custom algorithms or software that are central to the research but not yet described in published literature, software must be made available to editors and reviewers. We strongly encourage code deposition in a community repository (e.g. GitHub). See the Nature Portfolio guidelines for submitting code & software for further information.

Data

Policy information about availability of data

All manuscripts must include a data availability statement. This statement should provide the following information, where applicable:

- Accession codes, unique identifiers, or web links for publicly available datasets
- A description of any restrictions on data availability
- For clinical datasets or third party data, please ensure that the statement adheres to our policy

Data availability: The mass spectrometry proteomics data have been deposited to the ProteomeXchange Consortium (https://www.ebi.ac.uk/pride/archive) via the PRIDE partner repository with the dataset identifier PXD046081 (phosphoproteome approach) and the dataset identifier PXD079152 (affinity enrichment approach) 80. Transcriptomic data have been deposited at GEO (https://www.ncbi.nlm.nih.gov/geo/) with the accession number GSE300992 (GSM9073322-GSM9073332) and GSE326693. Both datasets are openly accessible.

Research involving human participants, their data, or biological material

Policy information about studies with human participants or human data. See also policy information about sex, gender (identity/presentation), and sexual orientation and race, ethnicity and racism.

Reporting on sex and gender	n.a.
Reporting on race, ethnicity, or other socially relevant groupings	n.a.
Population characteristics	n.a.
Recruitment	n.a.
Ethics oversight	n.a.

Note that full information on the approval of the study protocol must also be provided in the manuscript.

Field-specific reporting

Please select the one below that is the best fit for your research. If you are not sure, read the appropriate sections before making your selection.

- [x] Life sciences
- [ ] Behavioural & social sciences
- [ ] Ecological, evolutionary & environmental sciences

For a reference copy of the document with all sections, see nature.com/documents/nr-reporting-summary-flat.pdf

Life sciences study design

All studies must disclose on these points even when the disclosure is negative.

Sample size	The group size of animals in experimental groups was based on the assumed variation and difference using the online tool: http://www.biomath.info/power/ttest.htm to achieve a probability of a type I error of 0.05 and a probability of a type II error of 0.2
Data exclusions	No data were excluded from the analysis.
Replication	All experiments were conducted at least three times (independent biological replicates). The precise number of replicates is indicated in the legend text. Neonatal mice were analysed for each group from at least two separate litters.
Randomization	Mice were randomly assigned to experimental groups containing mice of different litters. For infection experiments, litters could not be devided between groups infected or non-infected or infected with two different bacterial strains.
Blinding	The evaluation and quantification of the histological stainings was done in a blinded fashion (sample type unknown to the person analysing the stainings).

Reporting for specific materials, systems and methods

We require information from authors about some types of materials, experimental systems and methods used in many studies. Here, indicate whether each material, system or method listed is relevant to your study. If you are not sure if a list item applies to your research, read the appropriate section before selecting a response.

Materials & experimental systems

n/a	Involved in the study	
[ ]	[x]	Antibodies
[ ]	[x]	Eukaryotic cell lines
[ ]	[ ]	Palaeontology and archaeology
[ ]	[x]	Animals and other organisms
[ ]	[ ]	Clinical data
[ ]	[ ]	Dual use research of concern
[ ]	[ ]	Plants

Methods

n/a	Involved in the study	
[ ]	[ ]	ChIP-seq
[ ]	[x]	Flow cytometry
[ ]	[ ]	MRI-based neuroimaging

Antibodies

Antibodies used	Immune cell analysis: CCD45-FITC (Clone 30-F11), Ly6C-PerCPCy5.5 (Clone HK1.4), Ly6G-PE (Clone 1A8), Ly6G-Spark NIR 685 (Clone 1A8), Ly6C-BV711 (HK1.4), CD11b-APC Cy7 (Clone N418), CD11b-BUV 395 (Clone M1/70), CD64-APC (Clone X54-5/7.1), CD64-PE Dazzle (Clone X54-5/7.1), MHCII-AF488 (Clone M5/114.15.2), MHCII-BV510 (Clone M5/114.15.2), PDL1-PE (Clone 10F.952), SiglecF-APCR700 (Clone 90/CD38; BD), Epcam-BV421 (Clone G8.8), CD3-FITC (Clone 17A2), CD19-FITC (Clone 6D5), (Biolegend)

	Intracellular cytokine staining: (Biolegend): CD45-APCR700 (Clone 30-F11; BD Biosciences), CD3-APCFire750 (Clone 17A2), PDL1-APC (Clone 10F.952), SiglecF-BB515 (Clone E50-2440; BD Biosciences), CD11c-BUV737 (Clone N418: BD Biosciences), CD64-PEDazzle (Clone 90/CD38), CD11b-BV786 (Clone M1/70), F480-PECy5 (Clone BM8), Epcam-BV421 (Clone G8.8), MHCII-BV510 (Clone M5/114.15.2), Ly6C-PerCP-Cy5.5 (Clone HK1.4), Ly6G-BV711 (Clone 1A8), CD80-BUV 805 (Clone 16-10A1; BD Biosciences), CD19-PECy7 (Clone 6D5), TNFα-PE (Clone MP6-XT22) antibody
Validation	All cytokine panels and indiviudal antibodies were tested and the staining optimised prior to the analysis using known immune cells/ immune cells with a known composition.

Eukaryotic cell lines

Policy information about cell lines and Sex and Gender in Research

Cell line source(s)	m-ICcl2 cells (Bens et al., Am J Physiol. 1996, PMID: 8764149 DOI: 10.1152/ajpcell.1996.270.6.C1666
Authentication	The cells were obtained from the original source (Alain Vandewalle, MD PhD) but not further authenticated, however, the phenotype (polarization, confluent growth, appearance) is very typical.
Mycoplasma contamination	ELISA for mycoplasma were conducted regularly (approx. every 6 months).
Commonly misidentified lines (See ICLAC register)	n.a.

Palaeontology and Archaeology

Specimen provenance	n.a.
Specimen deposition	n.a.
Dating methods	n.a.

☐ Tick this box to confirm that the raw and calibrated dates are available in the paper or in Supplementary Information.

Ethics oversight	n.a.

Note that full information on the approval of the study protocol must also be provided in the manuscript.

Animals and other research organisms

Policy information about studies involving animals; ARRIVE guidelines recommended for reporting animal research, and Sex and Gender in Research

Laboratory animals	Mus musculus C57BL/6J and N
Wild animals	none
Reporting on sex	Most experiments were conducted with neonatal mice at the age of 1-3 days. Prior to puberty a sex-dependent influence is not expected and the sex was not recorded. Experiments with adult were performed on female mice to be able to cohouse animals from different litters in one cage.
Field-collected samples	none
Ethics oversight	All animal experiments were performed in compliance with the German animal protection law (TierSchG) and approved by the local animal welfare committee (Niedersachsische Landesamt für Verbraucherschutz und Lebensmittelsicherheit Oldenburg, Germany; Landesamt für Natur, Umwelt und Verbraucherschutz, North Rhine Westfalia) under the code 84-02.04.2017.A397 and 84–02.04.2021.A043 including all approved changes.

Note that full information on the approval of the study protocol must also be provided in the manuscript.

Clinical data

Policy information about clinical studies

All manuscripts should comply with the ICMJE guidelines for publication of clinical research and a completed CONSORT checklist must be included with all submissions.

Clinical trial registration	n.a.
Study protocol	n.a.
Data collection	n.a.
Outcomes	n.a.

Dual use research of concern

Policy information about dual use research of concern

Hazards

Could the accidental, deliberate or reckless misuse of agents or technologies generated in the work, or the application of information presented in the manuscript, pose a threat to:

No	Yes	
☒	☐	Public health
☒	☐	National security
☒	☐	Crops and/or livestock
☒	☐	Ecosystems
☒	☐	Any other significant area

Experiments of concern

Does the work involve any of these experiments of concern:

No	Yes	
☒	☐	Demonstrate how to render a vaccine ineffective
☒	☐	Confer resistance to therapeutically useful antibiotics or antiviral agents
☒	☐	Enhance the virulence of a pathogen or render a nonpathogen virulent
☒	☐	Increase transmissibility of a pathogen
☒	☐	Alter the host range of a pathogen
☒	☐	Enable evasion of diagnostic/detection modalities
☒	☐	Enable the weaponization of a biological agent or toxin
☒	☐	Any other potentially harmful combination of experiments and agents

Plants

Seed stocks	n.a.
Novel plant genotypes	n.a.
Authentication	n.a.

ChIP-seq

Data deposition

☐ Confirm that both raw and final processed data have been deposited in a public database such as GEO.

☐ Confirm that you have deposited or provided access to graph files (e.g. BED files) for the called peaks.

Data access links<br>*May remain private before publication.*	n.a.
Files in database submission	n.a.
Genome browser session<br>(e.g. UCSC)	n.a.

Methodology

Replicates	n.a.

Sequencing depth: n.a.

Antibodies: n.a.

Peak calling parameters: n.a.

Data quality: n.a.

Software: n.a.

Flow Cytometry

Plots

Confirm that:

- [x] The axis labels state the marker and fluorochrome used (e.g. CD4-FITC).
- [x] The axis scales are clearly visible. Include numbers along axes only for bottom left plot of group (a 'group' is an analysis of identical markers).
- [x] All plots are contour plots with outliers or pseudocolor plots.
- [x] A numerical value for number of cells or percentage (with statistics) is provided.

Methodology

Sample preparation: Peyers patches and feces were removed, the intestine was opened longitudinally and transferred into 20 ml of HBSS/3% FBS with 2mM EDTA. The intestine was shaken twice at 150 rpm at 37°C for 20 min. After the second incubation the intestine was rinsed with PBS to remove the EDTA. The remaining intestine was enzymatically digested in RPMI (Gibco) containing 30 µg/ml LiberaseTM (Roche) and 100 µg/ml DNase (Roche) for 45min shaking at 37°C. Tissue pieces were filtered through a 100 µm nylon cell strainer (BD) to obtain a single cell suspension. Immune cells were separated using a Percoll gradient by centrifugation at 700 x g for 20 min. at room temperature. Cells were stained using the following antibodies CD45-BV510 (Clone 30-F11), CD45-PE Cy7 (Clone 30-F11), Ly6C-PerCPCy5.5 (Clone HK1.4), Ly6G-PE (Clone 1A8), Ly6G-Spark NIR 685 (Clone 1A8), Ly6C-BV711 (HK1.4), CD11b-APC Cy7 (Clone N418), CD11b-BUV 395 (Clone M1/70), CD64-APC (Clone X54-5/7.1), CD64-PE Dazzle (Clone X54-5/7.1), MHCII-AF488 (Clone M5/114.15.2), MHCII-BV510 (Clone M5/114.15.2), PDL1-PE (Clone 10F.952), SiglecF-APCR700 (Clone 90/CD38; BD), Epcam-BV421 (Clone G8.8), CD3-FITC (Clone 17A2), CD19-FITC (Clone 6D5), (Biolegend) and DAPI (Roth) for subsequent analytical flow cytometry.

Instrument: BD FACS Canto II

Software: FlowJo X

Cell population abundance: 1-30 % for immune cell type (60-80% including viability).

Gating strategy: As visualized in Suppl. Figure 2 and 5.

- [x] Tick this box to confirm that a figure exemplifying the gating strategy is provided in the Supplementary Information.

Magnetic resonance imaging

Experimental design

Design type: n.a.

Design specifications: n.a.

Behavioral performance measures: n.a.

Acquisition

Imaging type(s): n.a.

Field strength: n.a.

Sequence & imaging parameters: n.a.

Area of acquisition: n.a.

Diffusion MRI: - [ ] Used - [ ] Not used

Preprocessing

Preprocessing software: n.a.

Normalization	n.a.
Normalization template	n.a.
Noise and artifact removal	n.a.
Volume censoring	n.a.

Statistical modeling & inference

Model type and settings	n.a.
Effect(s) tested	n.a.

Specify type of analysis: ☐ Whole brain ☐ ROI-based ☐ Both

Statistic type for inference (See Eklund et al. 2016)	n.a.
Correction	n.a.

Models & analysis

n/a	Involved in the study
☐	☐ Functional and/or effective connectivity
☐	☐ Graph analysis
☐	☐ Multivariate modeling or predictive analysis

Functional and/or effective connectivity	n.a.
Graph analysis	n.a.
Multivariate modeling and predictive analysis	n.a.
