# Supplementary material for: Salmonella SopB suppresses post-transcriptionally regulated cytokine release to reduce early tissue inflammation and delay disease progression
Source: Nat Commun. 2026 Jul 6;17:5884. doi: 10.1038/s41467-026-74942-9 (PMC13338139; doi:10.1038/s41467-026-74942-9)
Supplement: Supplementary file 3 — Transparent Peer Review file [file 41467_2026_74942_MOESM3_ESM.pdf]

nature portfolio

Peer Review File

Salmonella SopB suppresses post-transcriptionally regulated cytokine release to reduce early tissue inflammation and delay disease progression

Corresponding Author: Professor Mathias Hornef

This file contains all reviewer reports in order by version, followed by all author rebuttals in order by version.

Version 0:

Reviewer comments:

Reviewer #1

(Remarks to the Author)
Salmonella bacteria invade the intestinal mucosa through the action of SPI-1-encoded effectors. In previous work, several of the present authors developed a neonate mouse model of per-oral Salmonella infection and used it to dissect the impact of specific SPI-1-effectors. They noted that Salmonella SopB did not appear to matter for the invasion process itself. In the current manuscript, Diab et al follow up on those findings and instead pinpoint a role for SopB in preventing excessive mucosal inflammation and delaying host death.

The manuscript demonstrates in multiple ways (survival analysis, mRNA and protein cytokine measurements, immune cell quantifications, pathology assessment) that a ΔsopB mutant strain causes “accelerated disease progression” and more pronounced inflammation than Salmonella wt in neonate mice. This fundamental finding is well documented and interesting and the complementation experiments ensure specificity of these phenotypes.

The authors have also conducted a large number of follow-up experiments in neonate mice, a mouse cell line, and a neonate murine intestinal epithelial organoid model. The results, presented in the second half of the figure material, contain several notable findings, such as the involvement of TNF-signaling, and the relevance of the SopB N-terminus for the SopB-linked suppression of excessive disease. However, some more detailed mechanistic claims are also being made, which are not always supported by data in a clear-cut way, and the manuscript would benefit from further clarifications and distillation.

Main comments:
1) As stated in the discussion (lines 626-633), the available literature is contradictory with regards to SopB impact on mucosal inflammation in in vivo and ex vivo models. Here, the authors use exclusively the neonate mouse model to make broad in vivo claims. It seems vital for the implications of this study to know if hyperinflammation and accelerated disease progression for the ΔsopB strain is also observed in adult mice, which the authors have experience from working with. Do they reproduce the findings in adult mice, in analogy to results in Hu et al 2019 (doi: 10.3389/fcimb.2019.00087)? If yes, this would strengthen the case for broadly relevant mechanisms. If no, then the conclusions in this manuscript should be narrowed to neonates.

2) In Fig 4 and Fig S3 and the corresponding results text, the authors home in on specific underlying mechanisms. They state that the results favor a relevance for Mlkl and necroptosis, while excluding other possibilities. However, the refutation of other signaling pathways relies on blunt survival analyses (Fig 4A-C, Fig 3A) with small differences between groups, and where both Casp-1 -/- and Asc -/- mice show a trend towards delayed disease kinetics (either for wt Salmonella, or for both strains).
It is also stated in this section that the “…iNOS (Nos2) remained unaltered in ΔsopB S. Typhimurium infected wildtype animals (Fig S3B and C) in contrast to previous reports (…”. Even if the significance testing in Fig S3C came out non-significant due to data spread, the trend and effect size when comparing mean values in Fig S3B actually looks similar to for example tnf transcript quantifications in Fig 4I that the authors deemed important.
In summary, this passage makes claims of a highly specific mechanism involving necroptosis and excluding other options, while the data suggest a more nuanced picture. I suggest to moderate the conclusions accordingly or provide more conclusive support for the claims.

3) For some datasets key to the claims, the experimental group sizes differ widely and some groups are too small (3 data points with large spread) to permit conclusive interpretation. There is a need to add additional data points and statistical reanalysis to support those claims. This applies for example to the following panels, but similar issues can be found elsewhere in the figure material:
i) Fig 1B-C: The claim here is that no difference in epithelial colonization can be seen between the strains., but the trend for day 1 looks clearly higher for ΔsopB, and no conclusions can be drawn for the complemented strain due to a small group size and large spread.
ii) Fig S1B: The claim here is that in epithelial cell culture a SopB invasion phenotype can be seen, but the small group sizes result in non-significant differences in the statistical testing.
iii) Fig 7D-F: The involvement of the SopB N-terminus in the main phenotype of the manuscript gains support from several different experimental types in Fig 7. Still, the four data points in the sopBL76P group in Fig 7D-F cluster in a bimodal way with large spread, where significance testing against the wt group would likely also reveal no difference. Considering the potential importance of the findings in Fig 7, it seems a shame to leave this ambiguous.

Additional comments:
4) Figure 1F and Fig S 1H: These panels present a z-score heat map (1F) based on absolute quantifications (S1H) for relevant cytokines and chemokines. For many of these, the absolute values are really low, so it seems doubtful that they land above the assay detection limit. Could the authors please clarify the detection limits (either from the assay supplier's documentation, or based on own experimental validation) to ensure that all those panels present meaningful data?

5) Fig 5H-I: It is hard to understand the TACE data presented in these panels. Considering the authors conclusion, would one not expect the TACE activity to be dramatically higher in the ΔsopB group (Fig 5I) compared to the wt group (Fig 5H)? Currently, comparisons are only made to the respective inhibitor-treated groups.

6) The methods section would at places benefit from further specifications to allow reproduction. For example: i) (lines 150-152) How were the bacteria grown for infection (LB, shaking or not)? ii) (lines 266-273) What specific media and supplements and in what concentrations were used for the neonatal intestinal epithelial organoid cultures? iii) (lines 232-234) How was the immune cell isolation done? Was an RBC lysis step included?

7) (Lines 446-447) The authors state "…goblet cell depletion and reduced MUC2+ signal in goblet cells indicating enhanced mucus secretion…". Considering the elevated inflammatory status, this phenotype appears more likely to be caused by the loss of goblet cells and incomplete differentiation of new replacements from progenitors in the ΔsopB-infected mice.

8) Please check for adherence to contemporary mouse cytokine and chemokine gene and protein names. For example, on the protein level: MCP-1 -> CCL2, KC -> CXCL1, IP-10 -> CXCL10.

Reviewer #2

(Remarks to the Author)
I co-reviewed this manuscript with one of the reviewers who provided the listed reports. This is part of the Nature Communications initiative to facilitate training in peer review and to provide appropriate recognition for Early Career Researchers who co-review manuscripts.

Reviewer #3

(Remarks to the Author)
In this manuscript, Diab et al describe a novel mechanism of how Salmonella interferes with immune responses at early stages of infection in a neonatal mouse infection model. They report that a novel biochemical activity for the type three secretion system effector SopB, a known inositol phosphatase. They find that in newborn mice, a mutant lacking SopB causes more pathology and mortality compared to the wild-type strain, and transcriptomics show an increased abundance of mRNAs encoding for pro-inflammatory cytokines/chemokines. Consistent with this data, recruitment of inflammatory cells, including inflammatory monocytes and neutrophils is increased in mice infected with the sopB mutant. TUNEL staining suggests that SopB decreases cell death, in particular in the epithelium. These phenotypes were abolished in mice lacking TNFR1 and MLKL, suggesting that SopB interferes with induction of necroptosis. SopB prevented Cxcl2 expression in a TNFR1 dependent manner. In cell culture experiments, SopB altered the TORC1 signaling pathway, and experiments inhibiting secretory autophagy suggest that SopB inhibits IL-18 secretion by secretory autophagy. This was independent of the known phosphatidylinositol phosphatase and phosphotransferase activity of SopB.
Overall, this study reports a previously unknown activity of the Salmonella effector protein SopB. The main conceptual advance is the discovery that of a pro-inflammatory pathway that is critical during early Salmonella infection, i.e. release of TNF and secretory autophagy-dependent secretion of IL-18 (and that SopB interferes with this pathway). The experiments are well designed and the conclusions supported by the data. The writing is clear and concise. I have no major concerns.

Minor concerns:
- It is unclear how Salmonella mutants were generated, or if these mutants had been reported before. This information should be provided in the materials and methods section.
- In the statistics section, the authors state that data (more than two groups) were analyzed using a One-way ANOVA or Kruskal-Wallis test; these are fundamentally different tests (parametric vs non-parametric) and it is not clear when one was chosen vs. the other test. Presumably the authors did test for normality of the underlying data, and if so, that should be stated

(what test?).
- Lines 454-473: It might be worth mentioning which pathways are defective in caspase1, caspase-8, and mlkl-deficient animals, to expand on the idea that different cell death pathways are being explored. This would particularly help readers which are not experts in the field of immunology.

Reviewer #4

(Remarks to the Author)
The manuscript by Diab et al. investigates how the Salmonella SPI-1 effector SopB modulates early intestinal innate responses. Using a neonatal oral infection model together with polarized epithelial cells, intestinal organoids, phosphoproteomics, transcriptomics and functional assays, the authors report that SopB suppresses early mucosal inflammation and delays disease progression by: (1) preventing activation of the TNFα-converting enzyme TACE/ADAM17 and thereby reducing TNFα shedding from enterocytes; and (2) inhibiting mTOR-dependent secretory autophagy to limit epithelial IL-18 release. They further report that this immunosuppressive activity requires an intact N-terminal domain of SopB but is independent of its canonical C-terminal phosphatase activity. The study is potentially high impact and addresses an important question, but several key mechanistic gaps and control experiments must be addressed to further improve the manuscript's rigor and overall quality.

Major comments
1. Lack of specificity control for SopB (i.e. strains with unrelated effector deletions as negative controls).
2. There is a lack of direct evidence linking SopB to TACE/ADAM17 regulation. The data presented are correlative. The authors should probe whether SopB physically or functionally interacts with TACE/ADAM17 or known regulators using co-immunoprecipitation or proximity labelling. If no direct interaction exists, experiments should clarify how SopB alters upstream signaling that controls TACE/ADAM17 activation.
4. It is unclear whether the described SopB-dependent immunosuppression is specific to neonates.

Version 1:

Reviewer comments:

Reviewer #1

(Remarks to the Author)
The authors have done an impressive job at tackling the reviewer critique. The added datasets, filling up of experimental groups, and further analyses, have significantly strengthened the study. Moreover, the comparisons to adult mice and clarifications in writing provide important additional context. No major issues remain.

Since the SopB-related inflammatory phenotype was not observed in adult mice, and considering the contradicting prior data in the literature on that point, it would be wise to make a final clarification in the article title. For example:

"Salmonella SopB suppresses post-transcriptionally regulated cytokine release to reduce early tissue inflammation and delay disease progression during neonatal infection"

Will leave it to the editor and the authors to discuss the appropriateness of that suggestion.

Reviewer #2

(Remarks to the Author)
I co-reviewed this manuscript with one of the reviewers who provided the listed reports. This is part of the Nature Communications initiative to facilitate training in peer review and to provide appropriate recognition for Early Career Researchers who co-review manuscripts.

Reviewer #4

(Remarks to the Author)
The authors have adequately and thoughtfully addressed my concerns and I have no further comments.

Open Access This Peer Review File is licensed under a Creative Commons Attribution 4.0 International License, which permits use, sharing, adaptation, distribution and reproduction in any medium or format, as long as you give appropriate credit to the original author(s) and the source, provide a link to the Creative Commons license, and indicate if changes were made.
In cases where reviewers are anonymous, credit should be given to 'Anonymous Referee' and the source.
The images or other third party material in this Peer Review File are included in the article’s Creative Commons license, unless indicated otherwise in a credit line to the material. If material is not included in the article’s Creative Commons license and your intended use is not permitted by statutory regulation or exceeds the permitted use, you will need to obtain permission directly from the copyright holder.
To view a copy of this license, visit https://creativecommons.org/licenses/by/4.0/

Reviewer's comments

Reviewer #1:

Salmonella bacteria invade the intestinal mucosa through the action of SPI-1-encoded effectors. In previous work, several of the present authors developed a neonate mouse model of per-oral Salmonella infection and used it to dissect the impact of specific SPI-1-effectors. They noted that Salmonella SopB did not appear to matter for the invasion process itself. In the current manuscript, Diab et al follow up on those findings and instead pinpoint a role for SopB in preventing excessive mucosal inflammation and delaying host death.

The manuscript demonstrates in multiple ways (survival analysis, mRNA and protein cytokine measurements, immune cell quantifications, pathology assessment) that a ΔsopB mutant strain causes "accelerated disease progression" and more pronounced inflammation than Salmonella wt in neonate mice. This fundamental finding is well documented and interesting and the complementation experiments ensure specificity of these phenotypes.

The authors have also conducted a large number of follow-up experiments in neonate mice, a mouse cell line, and a neonate murine intestinal epithelial organoid model. The results, presented in the second half of the figure material, contain several notable findings, such as the involvement of TNF-signaling, and the relevance of the SopB N-terminus for the SopB-linked suppression of excessive disease. However, some more detailed mechanistic claims are also being made, which are not always supported by data in a clear-cut way, and the manuscript would benefit from further clarifications and distillation.

*We are grateful for the overall positive judgement of the reviewer. As requested, we provide more mechanistic insight to support our claims (e.g. by demonstrating in vivo evidence for the plasma membrane translocation of ADAM17/TACE in mice infected with ΔsopB but not wildtype S. Typhimurium (**new Fig. 5i and j**), by the analysis of a direct interaction between SopB and ADAM17 (**new Suppl. Fig. 6d**, page 25, line 5-6, requested by reviewer #4, point 2), by an AlphaFold Multimer-augmented interactome analysis between proteins identified in our SopB affinity co-purification assay and the known ADAM17/TACE-associated proteins (**new Fig. 6d** for selected very high confidence interactions **and Suppl. Fig. 6b** for all pairwise comparisons) and by an integrated network connecting proteins of the SopB interactome (shown in **new Fig. 6c**) and kinases identified in the phosphoproteome approach) together with the ADAM17 complex members using AFM-augmented protein-protein interaction (PPIs) analysis with an interaction confidence of > 0.75 and curated kinase-substrate links from public databases. Moreover, we added a comparative RNA Seq analysis of intestinal epithelial cells after wt versus ΔsopB S. Typhimurium infection in Tnfrsf1a$^{-/-}$ mice that lack the TNFR1 (**new Suppl. Fig. 6a**). This additional analysis revealed that most genes induced in the absence of SopB in wildtype mice (Suppl. Fig. 1g, red dots) were no longer induced*

*in the absence of TNF signalling (pink dots in **new Suppl. Fig. 6a**) illustrating the important role of TNF for the SopB-mediated disease progression. Finally, we have modified the manuscript text to balance the statements made and enhance the clarity of our study.*

Main comments:
1) As stated in the discussion (lines 626-633), the available literature is contradictory with regards to SopB impact on mucosal inflammation in in vivo and ex vivo models. Here, the authors use exclusively the neonate mouse model to make broad in vivo claims. It seems vital for the implications of this study to know if hyperinflammation and accelerated disease progression for the ΔsopB strain is also observed in adult mice, which the authors have experience from working with. Do they reproduce the findings in adult mice, in analogy to results in Hu et al 2019 (doi: 10.3389/fcimb.2019.00087)? If yes, this would strengthen the case for broadly relevant mechanisms. If no, then the conclusions in this manuscript should be narrowed to neonates.

*As suggested, we have analyzed the phenotype of the ΔsopB mutant ATCC14028 S. Typhimurium strain in the adult infection model with streptomycin pretreatment (see also point 4, reviewer #4). We did not observed differences in the number of intraepithelial bacteria or the total spleen, liver, MLN or colon tissue or isolated small intestinal epithelial cells (SIEC) (**new Suppl. Fig. 3b**). In contrast to the findings by Hu et al. 2019 but consistent with the data by Hapfelmeier et al., 2004 in adult mice (Front Cell Infect Microbiol. 2019 and Infect Immun 2004, respectively), we did not observe enhanced expression of proinflammatory cytokines in isolated small intestinal epithelial cells (SIEC) (**new Suppl. Fig. 3c**) or total colon tissue (**new Suppl. Fig. 3d**) or increased numbers of tissue-infiltrating monocytes, neutrophils or macrophages at early time points after infection (**new Suppl. Fig. 3f**) or an increased tissue destruction (**new Suppl. Fig. 3e**) or body weight loss (**new Suppl. Fig. 3a**).*
We would like to stress that Hu et al. used the SL1344 strain of S. Typhimurium that exhibits a somewhat more virulent phenotype mainly due to an additional, more potent SopE effector molecule and stronger SPI1 expression (Clark et al., Microbiology 2011). Also, whereas S. Typhimurium in the neonatal mouse model mainly affects the small intestine, the adult mouse infection model is characterized mainly by an inflammation of the large intestine. As recommended, we describe and discuss our findings in the revised version of the manuscript and narrow our conclusions to neonates (page 21, line 24-page 22, line 2; page 31, line 3-8).

2) In Fig 4 and Fig S3 and the corresponding results text, the authors home in on specific underlying mechanisms. They state that the results favor a relevance for Mlkl and necroptosis, while excluding other possibilities. However, the refutation of other signaling pathways relies on blunt survival analyses (Fig 4A-C, Fig 3A) with small differences between groups, and where both Casp-1 -/- and Asc -/- mice show a trend towards delayed disease kinetics (either for wt Salmonella, or for both strains).
It is also stated in this section that the "...iNOS (Nos2) remained unaltered in ΔsopB S. Typhimurium infected wildtype animals (Fig S3B and C) in contrast to previous reports (...". Even if the significance testing in Fig S3C came out non-significant due to data spread, the trend and effect size when comparing mean values in Fig S3B actually looks similar to for example tnf transcript quantifications in Fig 4I that the authors deemed

important. In summary, this passage makes claims of a highly specific mechanism involving necroptosis and excluding other options, while the data suggest a more nuanced picture. I suggest to moderate the conclusions accordingly or provide more conclusive support for the claims.

*We agree with the reviewer's comment that although only Mlkl knockout mice (Fig. 4c) show a significantly prolonged survival after ΔsopB S. Typhimurium infection in our experiments, a trend towards a prolonged survival is also observed in Casp1 (Fig. 4a) and ASC (Suppl. Fig. 3a) knockout mice suggesting a more mixed mechanism of cell death following ΔsopB S. Typhimurium infection. As requested, we have added the organ load and intestinal epithelial Cxcl2 mRNA expression for Casp1, ASC and Mlkl knockout mice (**new Suppl. Fig. 4d-g**). No significant difference in the organ load and gene expression was noted suggesting that the immunosuppressive effect of SopB on TNF and chemokine expression is upstream of the epithelial cell death observed. A tendency of elevated Nos2 (Suppl. Fig. 3c) mRNA expression was noted again consistent with some contribution of pyroptosis to the observed phenotype. We have modified the text to account for this and in the revised version of the manuscript depict a more nuanced picture as suggested (page 22, line 10-14 and 15-21; page 23, line 1-4).*

3) For some datasets key to the claims, the experimental group sizes differ widely and some groups are too small (3 data points with large spread) to permit conclusive interpretation. There is a need to add additional data points and statistical reanalysis to support those claims. This applies for example to the following panels, but similar issues can be found elsewhere in the figure material:
i) Fig 1B-C: The claim here is that no difference in epithelial colonization can be seen between the strains., but the trend for day 1 looks clearly higher for ΔsopB, and no conclusions can be drawn for the complemented strain due to a small group size and large spread.
ii) Fig S1B: The claim here is that in epithelial cell culture a SopB invasion phenotype can be seen, but the small group sizes result in non-significant differences in the statistical testing.
iii) Fig 7D-F: The involvement of the SopB N-terminus in the main phenotype of the manuscript gains support from several different experimental types in Fig 7. Still, the four data points in the sopBL76P group in Fig 7D-F cluster in a bimodal way with large spread, where significance testing against the wt group would likely also reveal no difference. Considering the potential importance of the findings in Fig 7, it seems a shame to leave this ambiguous.

We agree with the reviewer's comment and have performed additional animal experiments to reach larger group sizes, re-analysed the data and now provide more robust evidence and statistical power.
*For example, we agree with the reviewer's comment and performed additional in vivo experiments and added more data points to the **modified Fig. 1b and c**. No statistically significant difference in the bacterial organ load was noted between mice infected with wt or ΔsopB S. Typhimurium. In addition, the respective data for the $\Delta sopE_2$ mutant was added for comparison and again showed no significant difference to wt S. Typhimurium (see point #1, reviewer 3).*

Also, we performed new in vitro experiments with more data points to the ***modified Suppl. Fig. 1b.*** *The results now reveal a significantly reduced epithelial invasion in the absence of SopB in vitro (and a reversed phenotype using a ΔsopB psopB complemented strain).*
Finally, we performed additional in vivo experiments and added more data points to the ***modified Fig. 7d-f and Fig. 7 g and h.*** *Epithelial gene expression in mice infected with the SopB N-terminal point mutation L76P was similar to mice infected with ΔsopB S. Typhimurium and also immune cell infiltration was clearly enhanced in mice infected with the SopB N-terminal point mutation L76P similar to ΔsopB S. Typhimurium-infected animals although some spread of the results was observed.*
In addition, we added additional data points in ***Fig. 1d and e*** *as well as* ***Fig. 5b****.*

Additional comments:
4) Figure 1F and Fig S 1H: These panels present a z-score heat map (1F) based on absolute quantifications (S1H) for relevant cytokines and chemokines. For many of these, the absolute values are really low, so it seems doubtful that they land above the assay detection limit. Could the authors please clarify the detection limits (either from the assay supplier's documentation, or based on own experimental validation) to ensure that all those panels present meaningful data?

We thank the reviewer for his comment. As suggested, we added dashed lines in the ***modified Suppl. Fig. 1h*** *to indicate the detection limit of the assay. Also, we revised the data shown in the* ***modified Fig. 1f*** *and* ***Suppl. Fig. 1h,*** *removing the rows and panels (IFNβ and IL-12 p70) showing values below the detection limit.*

5) Fig 5H-I: It is hard to understand the TACE data presented in these panels. Considering the authors conclusion, would one not expect the TACE activity to be dramatically higher in the ΔsopB group (Fig 5I) compared to the wt group (Fig 5H)? Currently, comparisons are only made to the respective inhibitor-treated groups.

*We thank the reviewer for this comment. We have repeated the measurement of the ADAM17/TACE activity with isolated intestinal epithelial cells. In the revised version of the manuscript, we show the raw data of the kinetic assay of the ADAM17 activity in the absence and presence of the highly specific ADAM17 inhibitor during the detection time (****new Fig. 5h)*** *and the ratio of the activity in the absence and presence of the ADAM17 inhibitor in* ***the modified Suppl. Fig. 6b****. The data show a higher overall enzymatic ADAM17 activity in epithelial cells from mice infected with ΔsopB as compared to wt S. Typhimurium and an inhibition by the ADAM17 activity only after ΔsopB S. Typhimurium infection. Importantly, please note that the substrate of this assay is not considered very specific by experts in the field, but the inhibitor activity is. We teamed up with two ADAM17 experts (Andreas Ludwig and Stefan Düsterhöft) to discuss this and performed additional analyses (see* ***new Fig. 5i-j, new Fig. 6d-e, and new Suppl. Fig. 6c****) to confirm the influence of SopB on the modulation of ADAM17 activity.*

6) The methods section would at places benefit from further specifications to allow reproduction. For example: i) (lines 150-152) How were the bacteria grown for infection (LB, shaking or not)? ii) (lines 266-273) What specific media and supplements and in what concentrations were used for the neonatal intestinal epithelial organoid cultures?

iii) (lines 232-234) How was the immune cell isolation done? Was an RBC lysis step included?

We thank the reviewer for his comment. As suggested, we added the requested information on bacterial culture for the infection experiments (page 6, line 22-25), intestinal epithelial stem cell organoid culture conditions (page 11, line 25-page 12, line 17), and immune cell isolation (page 9, line 14-18) in the material & methods section.

7) (Lines 446-447) The authors state "...goblet cell depletion and reduced MUC2+ signal in goblet cells indicating enhanced mucus secretion...". Considering the elevated inflammatory status, this phenotype appears more likely to be caused by the loss of goblet cells and incomplete differentiation of new replacements from progenitors in the ΔsopB-infected mice.

We agree with the reviewer that goblet cell loss might be the most likely reason for the reduced Muc2 staining. In fact, we had observed significantly increased IFNγ levels in ΔsopB S. Typhimurium-infected animals, a previously reported cause of infection-induced "goblet cell depletion" (e.g. Chan et al., Infect. Immun., 2013, PMID 24101690). We modified the text accordingly (page 21, line 16-18).

8) Please check for adherence to contemporary mouse cytokine and chemokine gene and protein names. For example, on the protein level: MCP-1 -> CCL2, KC -> CXCL1, IP-10 -> CXCL10.

We thank the reviewer for the comment and changed the cytokine and chemokine names as suggested in all figures, supplemental figures, and throughout the manuscript text (CCL2 for MCP1 in ***Fig. 1d-f, Fig. 7e*** *and* ***Suppl. Fig. 1h****; CXCL1 for KC and CXCL10 for IP10 in* ***Fig. 1f*** *and* ***Suppl. Fig. 1h****).*

Reviewer #2:
I co-reviewed this manuscript with one of the reviewers who provided the listed reports. This is part of the Nature Communications initiative to facilitate training in peer review and to provide appropriate recognition for Early Career Researchers who co-review manuscripts.

We thank the reviewer for the input to the evaluation of our work.

Reviewer #3:
In this manuscript, Diab et al describe a novel mechanism of how Salmonella interferes with immune responses at early stages of infection in a neonatal mouse infection model. They report that a novel biochemical activity for the type three secretion system effector SopB, a known inositol phosphatase. They find that in newborn mice, a mutant lacking SopB causes more pathology and mortality compared to the wild-type strain, and transcriptomics show an increased abundance of mRNAs encoding for pro-inflammatory cytokines/chemokines. Consistent with this data, recruitment of inflammatory cells, including inflammatory monocytes and neutrophils is increased in mice infected with the sopB mutant. TUNEL staining suggests that SopB decreases cell

death, in particular in the epithelium. These phenotypes were abolished in mice lacking TNFR1 and MLKL, suggesting that SopB interferes with induction of necroptosis. SopB prevented Cxcl2 expression in a TNFR1 dependent manner. In cell culture experiments, SopB altered the TORC1 signaling pathway, and experiments inhibiting secretory autophagy suggest that SopB inhibits IL-18 secretion by secretory autophagy. This was independent of the known phosphatidylinositol phosphatase and phosphotransferase activity of SopB.
Overall, this study reports a previously unknown activity of the Salmonella effector protein SopB. The main conceptual advance is the discovery that of a pro-inflammatory pathway that is critical during early Salmonella infection, i.e. release of TNF and secretory autophagy-dependent secretion of IL-18 (and that SopB interferes with this pathway). The experiments are well designed and the conclusions supported by the data. The writing is clear and concise. I have no major concerns.

We are grateful for the overall positive judgement of the reviewer.

Minor concerns:
1. It is unclear how Salmonella mutants were generated, or if these mutants had been reported before. This information should be provided in the materials and methods section.

We apologize for this mistake, a detailed description how the bacterial mutants used in this study were generated was added to the material & methods section (page 5, line 11-page 6, line 5) and the strains, oligos and plasmids used are listed and described in ***new Suppl. Table 1-3****.*

2. In the statistics section, the authors state that data (more than two groups) were analyzed using a One-way ANOVA or Kruskal-Wallis test; these are fundamentally different tests (parametric vs non-parametric) and it is not clear when one was chosen vs. the other test. Presumably the authors did test for normality of the underlying data, and if so, that should be stated (what test?).

Again, we apologize for not accurately describing the statistical tests used. We tested the larger datasets for normal distribution using the Shapiro-Wilk test and employed the one-way ANOVA test or student's t-test for normally distributed datasets (only Fig. 3e, Fig. 4i, Fig. 5l) and the Kruskal Wallis test or Mann-Whitney test for the majority of non-normally distributed datasets. This information is provided in the material and method section (page 19, line 7-10) and the statistical tests used are indicated for each panel in the respective figure and supplemental figure legends.

2. Lines 454-473: It might be worth mentioning which pathways are defective in caspase1, caspase-8, and mlkl-deficient animals, to expand on the idea that different cell death pathways are being explored. This would particularly help readers which are not experts in the field of immunology.

We thank the reviewer for this suggestion and have added this information in the manuscript text to better explain the different cell death pathways investigated (page 22, line 10-13; page 22, line 14-15, and page 22, line 20-23).

Reviewer #4:
The manuscript by Diab et al. investigates how the Salmonella SPI-1 effector SopB modulates early intestinal innate responses. Using a neonatal oral infection model together with polarized epithelial cells, intestinal organoids, phosphoproteomics, transcriptomics and functional assays, the authors report that SopB suppresses early mucosal inflammation and delays disease progression by: (1) preventing activation of the TNFα-converting enzyme TACE/ADAM17 and thereby reducing TNFα shedding from enterocytes; and (2) inhibiting mTOR-dependent secretory autophagy to limit epithelial IL-18 release. They further report that this immunosuppressive activity requires an intact N-terminal domain of SopB but is independent of its canonical C-terminal phosphatase activity. The study is potentially high impact and addresses an important question, but several key mechanistic gaps and control experiments must be addressed to further improve the manuscript's rigor and overall quality.

We are grateful for the overall positive judgement of the reviewer and have addressed the points raised.

Major comments
1. Lack of specificity control for SopB (i.e. strains with unrelated effector deletions as negative controls).

*As suggested, we have added a bacterial strain with an unrelated effector protein deletion (ΔsopE$_2$) to the analysis (**modified Fig. 1b-e and modified Suppl Fig.1c-f**). As expected, ΔsopE$_2$ S. Typhimurium do not show a phenotype similar to ΔsopB S. Typhimurium.*

2. There is a lack of direct evidence linking SopB to TACE/ADAM17 regulation. The data presented are correlative. The authors should probe whether SopB physically or functionally interacts with TACE/ADAM17 or known regulators using co-immunoprecipitation or proximity labelling. If no direct interaction exists, experiments should clarify how SopB alters upstream signaling that controls TACE/ADAM17 activation.

*As suggested, we performed an immunoprecipitation of SopB (FLAG-tagged) and stained for ADAM17/TACE. This analysis did not reveal a direct interaction (**new Suppl. Fig. 6d**). Conversely, an AlphaFold Multimer-augmented interactome analysis between the proteins identified in our SopB affinity purification assay (Fig. 6c) and the known ADAM17/TACE-associated proteins ADAM17, iRhom1/ RHBDF1, iRhom2/RHBDF2, and FRMD8 revealed multiple indirect high-confidence interactions, for example between the SopB co-purified protease cofactor PPP2R5B or the kinase CSNK1E and ADAM17/TACE itself (50:50 ipTM iPAE average score of over 0.9). The results of the selected interactions are shown in **new Fig. 6d** and the results of all tested pairwise combinations are shown **new Suppl. Fig. 6c** (page 27, line 3-16). In addition, we generated an integrated network connecting proteins of the SopB interactome (shown in Fig. 6c) and kinases identified in the phosphoproteome approach (shown in Fig. 6a) together with the ADAM17 complex members using AFM-augmented protein-protein*

*interaction (PPIs) analysis with an interaction confidence of > 0.75 and curated kinase-substrate links from public databases. This network visualised many indirect interactions, consistent with the observed influence of SopB on ADAM17 activity (**new Fig. 6e**). Finally, we added a comparative RNA Seq analysis of intestinal epithelial cells after wt versus ΔsopB S. Typhimurium infection in Tnfrsf1a$^{-/-}$ mice that lack the TNFR1 (**new Suppl. Fig. 6a**). This additional analysis revealed that most genes induced in the absence of SopB in wildtype mice (Suppl. Fig. 1g, red dots) were no longer induced in the absence of TNF signalling (pink dots in Suppl. Fig. 6a) illustrating the important role of TNF for the SopB-mediated disease progression.*

4. It is unclear whether the described SopB-dependent immunosuppression is specific to neonates.

*We thank the reviewer for this comment (see also point 1, reviewer #1). As requested we performed additional experiments to analyze the phenotype of a ΔsopB mutant S. Typhimurium ATCC14028 in the adult oral infection model (using the model published by Barthel et al., Infect Immun, 2003). Similar to the neonatal infection model, we did not observe differences in the number of intraepithelial bacteria or the total organ load in liver, spleen, MLN, and colon tissue as well as small intestinal epithelial cells (SIEC) (**new Suppl. Fig. 3b**). In contrast to our results in the neonatal mouse model, however, we did not observe enhanced expression of proinflammatory cytokines in SIEC (**new Suppl. Fig. 3c**), or total colon tissue (**new Suppl. Fig. 3d**) or increased numbers of tissue-infiltrating monocytes, neutrophils or macrophages (**new Suppl. Fig. 3f**) at early time points after infection or an increased tissue destruction (**new Suppl. Fig. 3Ee**) or an increased weight loss after infection of adult mice with ΔsopB S. Typhimurium (**new Suppl. Fig. 3a**).*
Notably, Hu et al. (Front Cell Infect Microbiol. 2019) recently described a more severe disease phenotype for the ΔsopB mutant SL1344 S. Typhimurium strain reminiscent of the phenotype of ΔsopB mutant ATCC14028 S. Typhimurium in our neonatal infection. However, Hapfelmeier et al. (2004) did not detect a significant phenotype of ΔsopB mutant S. Typhimurium also using the SL1344 strain and a previous analysis of a ΔsopB S. Dublin strain in combination with a bovine intestinal loop model resulted in a reduced rather than enhanced immune cell infiltration (Galyov et al., 1997). The discrepancy of the results is currently not clear. The accelerated disease phenotype might require a stronger proinflammatory context (exhibited by the more virulent SL1344 strain in adults [Clark et al., Microbiology 2011] or in our neonatal infection model). To account for the reviewer's point, we discuss these findings in the context of the publication by Hu et a. and narrow our conclusions to neonates (page 30, line 22-page 31, line 8).
